# In-situ chlorine radical generation from captured Cl^-^ for redox synergy in electrocatalytic hydrodehalogenation

**DOI:** 10.1038/s41467-026-77253-1

**Published:** 2026-09-04

**Authors:** Chuyan Deng, Yujun Zhou, Xin Cai, Xuan Zhang, Yunlong Wang, Junwen Qi, Zhigao Zhu, Yue Yang, Jiansheng Li

**Affiliations:** 1 School of Environmental and Biological Engineering, Nanjing University of Science and Technology, Nanjing, China; 2 IIHR-Hydroscience & Engineering, University of Iowa, Iowa City, IA USA; 3 Department of Mechanical Engineering, University of Iowa, Iowa City, IA USA

**Keywords:** Pollution remediation, Environmental chemistry

## Abstract

Electrocatalytic dehalogenation can effectively detoxify chlorinated organic pollutants (COPs) by cleaving C–Cl bonds in wastewater treatment, however, it often fails to achieve complete mineralization. In particular, the fate of the released Cl^-^ has often been overlooked and whether they can be recycled and reutilized to facilitate pollutant degradation has remained unclear. Herein, a reduction–oxidation cascade system is constructed, in which Cl^-^ liberated during electrocatalytic reduction is directly converted into reactive chlorine species (RCS) during the subsequent oxidation step, enabling complete mineralization of organic pollutants. Quenching experiments and electron paramagnetic resonance spectroscopy (EPR) confirm the in situ reutilization of detached Cl^-^. Laser scanning confocal microscopy (LSCM) visualizes the adsorption and transformation of Cl^-^ on the electrode surface, while ab initio molecular dynamics (AIMD) calculations reveal the interfacial concentration distribution of Cl^-^ and the energy profiles of its migration. This work establishes a cascade electrocatalytic strategy that couples reduction and oxidation, enabling chlorine reutilization and providing a sustainable pathway for the removal and resource recovery of COPs.

## Abstract (web summary)

A redox cascade strategy converts waste chloride into reactive chlorine species during electrocatalytic treatment, achieving complete mineralization of toxic chlorinated pollutants.

## Introduction

Chlorinated organic compounds are widely used as crucial raw materials in the production of dye intermediates, disinfectants, preservatives, and other products^1,2^. Due to their highly toxic and bioaccumulative nature, these compounds can pose significant ecological risks and adversely affect human health upon release into aquatic environment, even at trace concentrations as low as parts per billion (ppb)^3–5^. Since the Cl atom on the benzene ring exerts an electron-withdrawing effect, it lowers the energy of the highest occupied molecular orbital (HOMO) of chlorinated organic pollutants (COPs), making them resistant to complete oxidative degradation^6^. Electrocatalytic hydrogenation dehalogenation (ECH) is a green chemical technology that utilizes electrical energy to drive catalytic reactions^7^. In this process, reactive hydrogen (H*) can replace Cl in COPs with H, enabling achieving pollutant degradation or functional group conversion of organic compounds^8^. However, simply removing Cl from pollutants hardly achieves complete mineralization and the released Cl^-^ in the solution may poison the catalyst, compromising the process efficiency^9,10^. Therefore, how to achieve the further complete degradation and mineralization of pollutants has attracted extensive attention. A promising strategy to address this challenge is the integrated reduction and oxidation processes^1,9^. This synergistic strategy first transforms COPs into more readily oxidizable intermediates via cathodic reductive dechlorination mediated by H*, which are subsequently mineralized at the anode^5,6,9^. It overcomes the issue of incomplete mineralization in mere reduction methods and enhances the degradation of persistent COPs that are recalcitrant to oxidation alone. However, current research has focused almost exclusively on oxidation via ·OH generation, while the influence of Cl^-^ as a cathodic reduction product and its potential reutilization pathways have received scant attention.

In saline wastewater, the chlorine evolution reaction (CER) is commonly empolyed to degrade organic pollutants by converting chloride ions (Cl^-^) into reactive chlorine species (RCS) for advanced oxidation^11,12^. RCS refers to a series of highly reactive and strongly oxidizing chlorine radicals and chlorine-related species, including chlorine radicals (Cl·), chlorine oxide radicals (ClO·), and chlorine radical anions (Cl_2_^-^), etc. They play an important role in various advanced oxidation technologies, especially in the degradation of organic pollutants and disinfection processes^13,14^. Compared to ·OH, RCS have a lower redox potential and longer half-life times, which endows them with greater selectivity, activity, and substrate-specific oxidation ability^15,16^. Notably, ·OH generation requires oxygen participation, which is detrimental to the ECH process. Therefore, if the Cl^-^ released during electro-reduction process could be directly converted into RCS to oxidize the dechlorination products further, it would promote the pollutant degradation and enable the green, secondary utilization of by-products. More importantly, in conventional EHDC systems, the released Cl^-^ is commonly regarded as a detached byproduct and a potential source of active-site poisoning, and previous studies have mainly addressed this issue by improving the chloride tolerance of Pd-based cathodes through catalyst design or additives. In contrast, the present tandem strategy prevents persistent chloride accumulation by further converting the released Cl^-^ in situ at the anode, thus offering a process-level advantage in mitigating electrode poisoning beyond conventional cathode-only optimization^10,17^. However, this interfacial effect is often overlooked, and whether the released chlorine can truly be reused remains understudied. This is primarily because the low concentration of Cl^-^ released from contaminant is easily disregarded. Besides, the mismatch of the anode and cathode electrode potentials as well as the irrationality of the electrochemical structure which exhibit low mass-transfer efficiency make it challenging to utilize the detached chloride ions and precisely control the reaction sequence.

Therefore, we aimed to construct a cascade electrochemical reactor to achieve the in situ utilization of chloride ions released during dehalogenation. The pollutant first undergoes dehalogenation at the cathode through ECH process and then further oxidized at anode by RCS generated from Cl^-^. To enable tight synergy between the cathode and anode, two key challenges must be addressed. First is the selection of appropriate electrode materials. In our previous study, PdCo bimetallic electrode demonstrated excellent dehalogenation performance and capable of removing most of the Cl^-^^18^. For the anode material, Ru-based dimensionally stable anodes (DSAs), represented by RuO_2_/Ti electrodes, have been extensively studied for their superior catalytic activity and stability in accelerating the anodic CER^19^. Since the electrochemical reaction occurs mainly on the electrode surface, enhancing the mass-transfer efficiency is the another essential problem^20^. To expose more electrochemically active sites and intensify mass transfer, flow-through configuration was employed. This design reduces the thickness of the diffusion boundary layer for key species (such as H*), thereby improving mass transport across the system^21^. After cathodic dehalogenation, the released Cl^-^ was supposed to migrate to anode by both electrostatic attraction and the forced convective flow field of the reactor, which enhances the possibility of its conversion to RCS for pollutant oxidation. By precisely tuning the reaction potentials, the synergistic efficiency of ECH and CER was expected to be maximized. To validate this strategy, 2,4,6-trichlorophenol (2,4,6-TCP), which is classified as a persistent organic pollutant, was chosen as the target contaminant. It contains three chlorine atoms and its high post-dechlorination chloride concentration facilitates direct verification of reaction feasibility and mechanistic investigation.

In this study, we fabricated a cascade chlorine cycling electrode reactor using PdCo/CP as cathode and RuO_2_/Ti as anode. We explored an equilibrium potential that promoted simultaneous H* generation and CER, while suppressing side reactions such as the oxygen evolution reaction (OER). Through electron paramagnetic resonance (EPR) and laser scanning confocal microscopy (LSCM) analyses, we confirmed that the detached Cl^-^ could be in situ excited into RCS. The respective contributions of the reduction and oxidation processes to the degradation of 2,4,6-TCP was further determined. Through in situ experiments, liquid chromatography-mass spectrometry (UPLC-MS), and ab initio molecular dynamics (AIMD) simulations, the pathway and mechanism for highly efficient dechlorination and deep mineralization under the combined action of electric and fluid fields were elucidated. This study offers a strategy that utilizes dechlorinated products in situ by transforming waste chloride into a self-generated oxidant. It initiates a self-sustaining degradation cycle and significantly promoting the deep mineralization of COPs in environment.

## Results

### Structural characterization and H* generation ability of PdCo/CP cathode

**Fig. 1:**
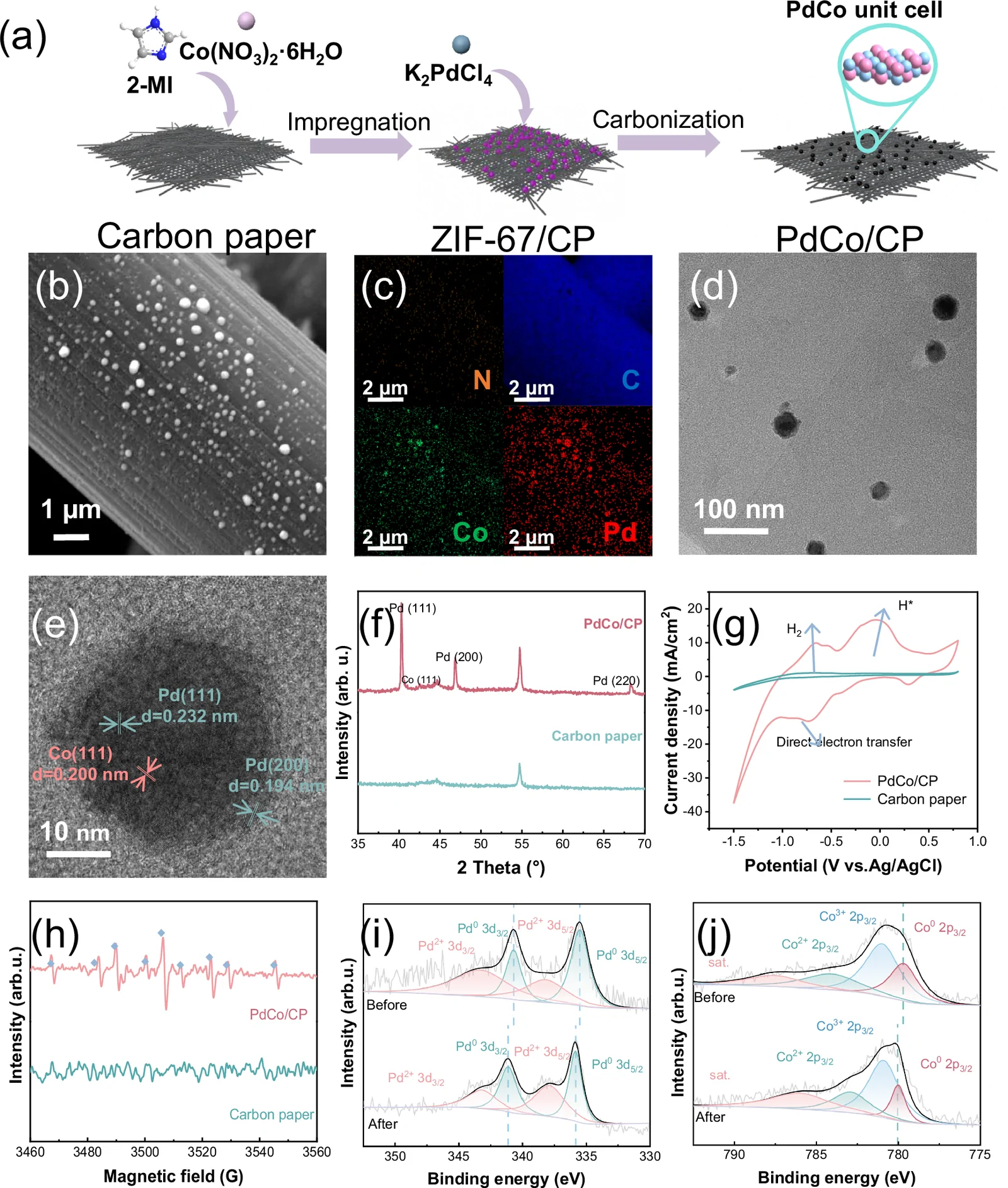
Synthesis and characterization of PdCo/CP. **a** Schematic illustration of the synthetic procedures of PdCo/CP. **b** SEM images of PdCo/CP. **c** EDS image of PdCo/CP. **d**,** e** (**d**) TEM and (**e**) HRTEM images of the PdCo/CP. **f** The XRD images of the PdCo/CP and CP electrode. **g** The CV curves of PdCo/CP and CP electrodes. **h** EPR spectra of H* trapped by DMPO. **i**,** j** (**i**) Pd 3d XPS spectra and (**j**) Co 2p XPS spectra of PdCo/CP before and after degradation. Representative SEM, TEM, and HRTEM images showing the surface morphology of PdCo/CP. The experiment was independently repeated 3 times with similar results.

The cathode PdCo/CP bimetallic electrode were prepared via a modified co-impregnation/reduction strategy reported in our previous work (Fig. 1a)^18^. The surface structure and morphology were further characterized. Compared to the bare carbon fibers (~10 μm diameter, Supplementary Fig. 2), the electrode after co-impregnation/reduction showed a uniform, in situ growth of PdCo particles (Fig. 1b). EDS analysis identified Pd, Co, C, O, and N as the sole components with no detectable impurities (Supplementary Fig. 3). Corresponding elemental mapping revealed a homogeneous distribution of Pd and Co within the PdCo particles, indicating the successful synthesis of the bimetallic material (Fig. 1c). The PdCo/CP was further characterized by TEM and HRTEM. As shown in Fig. 1d, the well-defined spherical particles were uniformly dispersed on the carbon support. HRTEM (Fig. 1e) showed lattice fringes with spacing of 0.232 nm, 0.194 nm, and 0.200 nm, corresponding to Pd(111), Pd(200), and Co(111) planes, respectively^22–24^. It confirms the good crystallinity and integration of Pd and Co, and reveals the exposure of the highly active Pd(111) facet. The identified Pd(111) is recognized as an ideal surface for H* generation due to its optimal d-band centre, and the increase in d-spacing is likely to enhance the material’s capacity for adsorbing pollutants^25–27^. The XRD patterns of the PdCo/CP and CP were shown in Fig. 1f, Compared to CP, PdCo/CP exhibited distinct diffraction peaks corresponding to the crystal planes of Pd and Co, with sharp peak shapes, indicating that the particles possessed excellent crystallinity. The diffraction peaks at 40.28°, 46.85°, and 68.27° corresponded to Pd(111), Pd(200), and Pd(220), respectively, while the diffraction peak corresponding to Co(111) appeared at 44.34°, which was consistent with the HRTEM results in Fig. 1e^28^. Through the co-impregnation/reduction method, the bimetallic Pd-Co was successfully prepared and exhibited excellent dispersion.

The performance of PdCo/CP as a cathode for H* generation was evaluated by CV test. In the potential range of −0.5 to 0.0 V (vs. Ag/AgCl), the PdCo/CP electrode exhibited a characteristic H* generation peak (Fig. 1g). When scanned to −1.0 V (vs. Ag/AgCl), an additional reduction peak emerged, corresponding to the direct reduction of 2,4,6-TCP. In contrast, no peaks were observed in the CV curves of carbon paper. These results indicate that the reduction of 2,4,6-TCP at the PdCo/CP cathode involves the concerted action of an H*-mediated indirect pathway and direct electron transfer^8,24^. To directly confirm H* generation in the removal of 2,4,6-TCP, EPR with DMPO as a spin trap was conducted in a 50 mM Na₂SO₄ solution^28^. As shown in Fig. 1h, the PdCo/CP cathode produced a characteristic H* nine-peak signal, whereas carbon paper yielded no significant peak. The direct evidence of H* generation from EPR is consistent with the CV data, thus jointly demonstrating the occurrence of an H*-dominated ECH process on the cathode^27^.

The surface chemical composition and electronic structure of the electrodes were characterized by XPS. The survey scan of PdCo/CP in Supplementary Fig. 4 revealed the presence of Pd, Co, O, N, and C, consistent with the elemental composition identified by EDS in Fig. 1c. High-resolution XPS spectra of the Pd 3d and Co 2p orbitals of PdCo/CP before and after the reaction are shown in Fig. 1i and j, respectively. In Fig. 1i, the Pd 3d spectrum were split into 3d_5/2_ (335.6 eV) and 3d_3/2_ (340.8 eV) states due to spin-orbit splitting^29^. The Pd 3d_5/2_ and Pd 3d_3/2_ peaks can both be deconvoluted into two peaks, corresponding to Pd^0^ and Pd²⁺, respectively^30^. After the reaction, a slight shift toward higher binding energy was observed, accompanied by a decrease in the Pd^0^ peak area and an increase in the Pd^2+^ contribution. This change was attributed to the oxidation of Pd^0^, which served as the primary active species during the reaction^31^. Similarly, in the high-resolution XPS spectra of the Co 2p orbital, the area of Co^0^ slightly decreased (Fig. 1j). Previous studies have demonstrated that Co may serve as an excellent active site for HER, and the aforementioned results confirmed that Co possessed the capacity to generate H*^32,33^. The incorporation of Co into ZIF-67 enhances the dispersion of Pd, exposing more active sites to increase hydrogen production capacity. Co itself also possesses hydrogen-producing capability, thereby enabling a reduction in the consumption of precious metals^34^. The obtained PdCo/CP exhibits an exceptional capacity for atomic hydrogen production and demonstrates considerable stability.

### Dechlorination-oxidation tandem reaction for complete degradation of 2,4,6-TCP

**Fig. 2:**
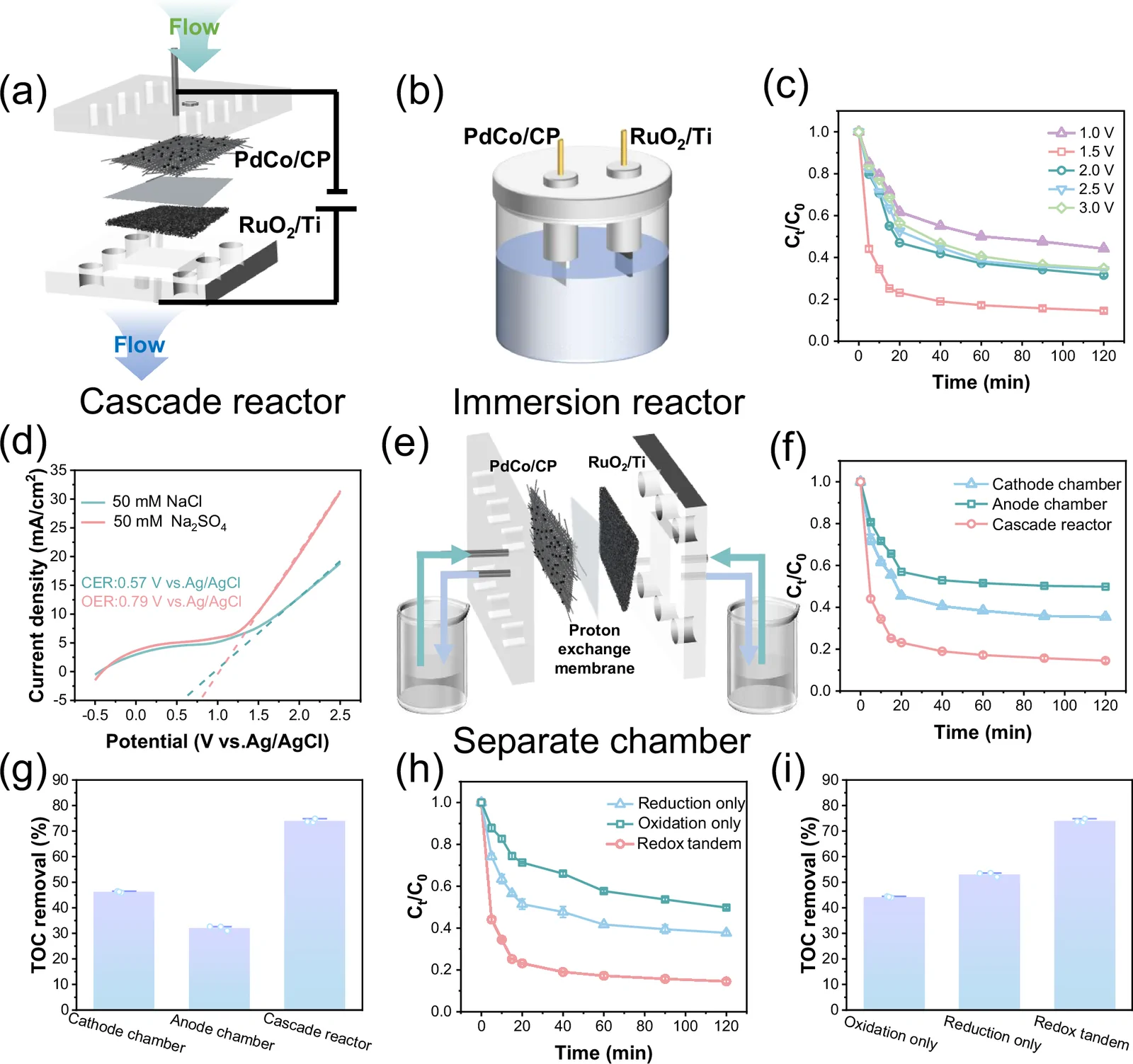
Electrocatalytic degradation performance of the cascade reactor. **a**,** b** Schematic diagram of **a** a cascade reactor and **b** an immersion reactor. **c** 2,4,6-TCP dechlorination performance on PdCo/CP electrodes under defferent applied potential. **d** LSV curves in different electrolytes. **e** Schematic diagram of a membrane-separated dual-compartment cell. **f**,** g** (**f**) Degradation and (**g**) TOC removal of 2,4,6-TCP in anode and cathode chambers. **h**,** i** (**h**) Performance and (**i**) TOC removal in oxidation only, reduction only, and redox tandem system. Conditions: 50 mM Na_2_SO_4_ solution; pH 4; initial 2,4,6-TCP concentration 20 mg/L; applied potential: 1.5 V). Data are presented as mean ± s.d. from *n* = 3 independent experiments (reactions were independently prepared and run on separate days). *n* represents the number of independent experiments, not technical replicates. Source data are provided as a Source data file.

In order to investigate whether the released Cl^-^ from 2,4,6-TCP could be transformed into strongly oxidizing species for further utilization, a homemade cascade chlorine cycling electrode reactor was fabricated. As shown in Fig. 2a, the as-prepared PdCo/CP electrode served as the cathode and RuO_2_/Ti was functioned as anode. Since the solution was forced to flow from cathode to anode, it imposes a spatially ordered reaction sequence in this flow-through reactor and the released Cl^-^ was mandatory migrated to the anode surface and likely to converted into RCS, thereby in turn facilitate further oxidation of 2,4,6-TCP and its intermediates. By contrast, conventional immersed electrode reactors (Fig. 2b) suffer from severely limited mass transfer efficiency, which fails to ensure close contact between pollutants and active sites and prevents the reaction from proceeding stepwise^35^. As shown in Supplementary Fig. 5, degradation experiments were conducted in both immersion reactor and cascade reactor. The degradation efficiency of the cascade reactor reached 85.5%, which was 14% higher than that of the immersion reactor, demonstrating the significantly superior performance of the cascade reactor. The extremely short electrode spacing and high surface-to-volume ratio of the electrodes reduced the mass transfer distance, thus greatly enhanced mass transfer efficiency and made full use of active sites. Furthermore, the sequential reduction-oxidation process facilitates more effective degradation and removal of pollutants^36,37^.

To ensure proper cathode-anode matching by enabling the H* generation and CER reactions to proceed concurrently, the applied voltage was carefully adjusted. The CV curves in Supplementary Fig. 6 reveal no oxidation peaks in the presence or absence of 2,4,6-TCP, indicating that direct electron transfer plays a negligible role and the primary effect of oxidation stems from RCS. As shown in Fig. 2c, the degradation efficiency in 120 min react time was only 55.8% when the applied voltage was 1.0 V. When the voltage increased to 1.5 V, the degradation efficiency significantly improved to 85.5%, and the initial rate constant (k_obs_) rose from 0.012 min^−1^ to 0.056 min^−1^. However, further increasing the voltage to 2.0 V caused a substantial 17% decrease in degradation efficiency. As the voltage was further elevated to 3.0 V, the efficiency gradually declined from 68.5% to 65.3%. Voltage and removal efficiency were not a simple linear relationship. LSV tests were conducted in both 50 mM NaCl and Na_2_SO_4_ electrolytes. As shown in Fig. 2d, the potential required to initiate CER was lower than that for OER, suggesting that CER was more readily generated compared to other reactive oxygen species (ROS)^38^. During the electrocatalytic degradation process, the applied voltage must reach a certain value to initiate specific electrode reactions. For example, the oxidation potential of ·OH is 2.7 V, while those of Cl·, Cl₂^·-^, and ClO· are lower, at 2.4, 2.0, and 1.5–1.8 V, respectively^13,39^. Herein, the lower voltage such as 1.0 V in the experiment could not trigger the generation of H* or RCS and lead to low pollutants removal efficiency. In practical applications, higher voltages are required to overcome the overpotential of the reaction. The magnitude and polarity of the applied voltage also influence the occurrence of side reactions. If the voltage is too high, it may trigger side reactions unrelated to the target reaction, consuming electrical energy and producing unnecessary by-products^7,40^. This was why degradation efficiency actually decreased when the voltage exceeded 1.5 V. By matching the applied voltage to the H* and RCS production, specific oxidation or reduction reactions can be selectively promoted while suppressing others, thereby optimizing the synergistic effect between the two electrodes. Subsequently, experiments were carried out without applied potential. Under this condition, the removal efficiency of 2,4,6-TCP was only 6.8%, and the corresponding TOC removal efficiency was merely 0.54%, both of which were negligible compared with the performance obtained at 1.5 V (Supplementary Figs. 7 and 8). These results indicate that the degradation observed in the tandem system mainly originated from the electrochemical process rather than from adsorption or other non-electrochemical effects.

To further evaluate the stability of the electrodes, cycling experiments were performed using the same electrode. As shown in Supplementary Fig. 9, after ten consecutive cycles, the degradation efficiency still remained at approximately 85%, while the leaching concentrations of Co, Pd, and Ru were only 0.02, 0.01, and 0.01 mg/L, respectively, demonstrating that the system can operate stably.

To further elucidate the functions of the tandem reaction, control experiments were carried out in a reactor whose chamber was divided into two sections by a proton exchange membrane, with electrolyte circulating independently in each compartment (Fig. 2e). The proton exchange membrane can minimize the crossover of reactive intermediates between the two electrodes, so that the individual contribution of the RuO_2_/Ti anode and PdCo/CP cathode could be evaluated separately. As shown in Fig. 2f, the removal efficiencies of 2,4,6-TCP at the anode and cathode were 50.1% and 64.7%, respectively. Both electro-reduction and electro-oxidation alone yield suboptimal removal efficiency, whereas sequential treatment involving cathodic reduction followed by anodic oxidation significantly enhances the degradation performance. Combined with TOC measurements (Fig. 2g), the anode alone achieved only 32.07% TOC removal, substantially lower than the 46.20% removal attained by the cathode alone. This confirms that cathodic degradation of 2,4,6-TCP proceeds mainly via reductive dechlorination, with limited direct mineralization into smaller molecules^41,42^. Although oxidation can directly mineralize pollutants, its efficiency is constrained by the high resistance of COPs to direct oxidation^5^.

Furthermore, since the initial electrolyte did not contain Cl^-^, it proves that the RCS generated in the series reaction does indeed enhance the oxidation capacity. The sequential cascade of reduction-oxidation enables efficient two-step processing: reductive dehalogenation first converts 2,4,6-TCP into more oxidizable and less toxic phenolic intermediates, which are then mineralized via subsequent anodic oxidation. This integrated redox cascade optimizes the overall degradation efficiency^39,43^.

Subsequently, the cathode and anode within the cascade reactor were replaced with carbon paper to investigate the specific contribution of oxidation-only and reduction-only. As shown in Fig. 2h, the degradation efficiencies of oxidation-only, reduction-only, and redox synergy were 50.2%, 62.3%, and 85.5%, respectively. These results indicated that oxidation had a poor degradation effect on 2,4,6-TCP. It can be attributed to the presence of three electronegative chlorine atoms on the benzene ring, which lower the ring’s electron density and enhance its chemical stability, thereby hindering direct oxidize^7^. Moreover, the high bond-dissociation energies and short bond lengths of C–Cl bonds contribute to the exceptional thermal and chemical stability of the aromatic structure, making it resistant to electrochemical cleavage^44^. Intermediate products generated during the oxidation of 2,4,6-TCP, such as catechol, hydroquinone, and benzoquinone (BQ), possess high redox potential and would resist further oxidation, which limits complete mineralization^1^. In contrast, reduction promoted faster and more effective dechlorination, and the redox synergy demonstrated superior degradation performance. In addition to the low efficiency of electrooxidation, another reason is that electrooxidation may not fully dechlorinate 2,4,6-TCP, leaving chlorinated organic products that are recalcitrant to further degradation^1,45^. This explains the relatively low TOC removal (44.19%) observed in the oxidation-only experiment (Fig. 2i). As shown in Fig. 2i, the TOC removal efficiency of reduction was 52.99%, which was significantly lower than the 73.97% achieved by redox tandem. This confirms that redox tandem enables deep mineralization of pollutants following dechlorination, thereby leading to their complete removal. However, a minor fraction of the Cl^-^ released during dechlorination may undergo anodic conversion to RCS, oxidizing the dechlorination products or participate in direct electron transfer pathways. Consequently, it exhibits higher TOC removal compared to the pure cathode chamber. The energy consumption per mg of TOC removed in our system was calculated to be 0.022 J/mg TOC, this indicates that the system is significantly more energy-efficient than conventional electro-oxidation processes^46^. Here, a cascade reactor with matched cathode and anode voltages were fabricated. It achieved highly efficient removal by first dechlorinating 2,4,6-TCP into an intermediate product readily oxidized for removal, followed by the complete oxidation of the removed chloride ions at the anode into RCS to eliminate the contaminant through a tandem reaction.

### Determination of RCS generated from released Cl^-^

**Fig. 3:**
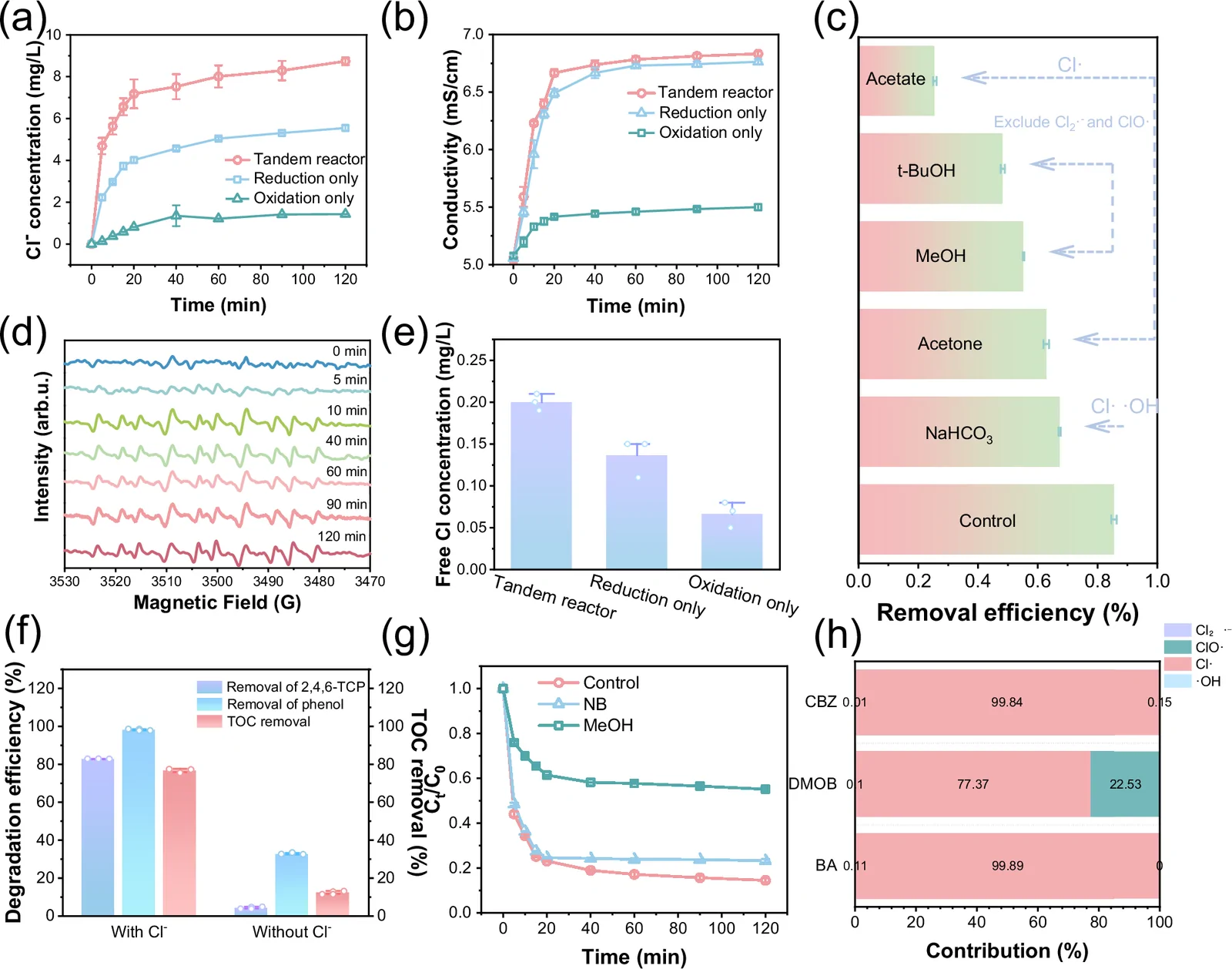
Evidence of the conversion of free chlorine ions to RCS. **a**, **b** (**a**) Cl^-^ concentration and (**b**) time-dependent variation in electrical conductivity in oxidation only, reduction only, and tandem reactor. **c** 2,4,6-TCP quenching experiment in tandem reactor. **d** EPR spectra of RCS trapped by PBN. **e** Free Cl concentration in oxidation only, reduction only, and tandem reactor. **f** The degradation efficiency and the TOC removal of simulated electrolyte after reduction-only reaction in the presence or absence of Cl⁻. **g** Quenching of ·OH: [MeOH] = [NB] = 5 mM. **h** Relative radical contributions to the probe decay of BA, DMOB, and CBZ derived from probe-kinetic analysis (From left to right, these numbers represent ·OH, Cl·, ClO·, and Cl_2_·⁻). Data are presented as mean±s.d. from *n* = 3 independent experiments (reactions were independently prepared and run on separate days). *n* represents the number of independent experiments, not technical replicates. Source data are provided as a Source data file.

Under the comparative treatments of only oxidation reaction, only reduction reaction, and tandem redox experiments, the concentration of Cl^-^ in the solution was measured. As shown in Fig. 3a, the free Cl^-^ concentration was 1.43 mg/L for oxidation only, significantly lower than that for reduction only (5.55 mg/L) and tandem reaction (8.74 mg/L). As shown in Fig. 2h, the degradation efficiency under reduction-only conditions was 62.3%, with a theoretical dechlorination value of 6.7 mg/L that closely approximated the experimental data. As shown in Supplementary Fig. 10, after 10 min of reaction, a clear Cl· signal was detected only in the system containing 2,4,6-TCP, while no obvious signal was observed in the absence of the pollutant. This comparison suggests that the Cl^-^ in solution originated from the dechlorination of 2,4,6-TCP and was subsequently transformed into Cl· during the reaction. This indicates that only oxidation can’t achieve complete removal of Cl from 2,4,6-TCP, suggesting incomplete degradation during the oxidation process and the presence of chlorinated intermediates. In contrast, the reduction process can remove as much Cl as possible from 2,4,6-TCP, which will significantly reduce its toxicity and promote its further degradation. As shown in Fig. 3b, the conductivity exhibited an increasing trend with reaction progression, rising rapidly within the initial 20 min before gradually stabilizing. The conductivity variation in the tandem redox reaction closely parallels the trend in Cl^-^ concentration depicted in Fig. 3a. Within the only reduction system, conductivity similarly demonstrated a marked increase. This behavior is mainly attributed to the continuous release of Cl⁻ during the cleavage of C–Cl bonds in 2,4,6-TCP, since Cl⁻ possesses a relatively high molar conductivity and therefore contributes significantly to the increase in solution conductivity^47^. Therefore, the conductivity increase can be regarded as an indirect indicator of Cl⁻ accumulation originating from pollutant dechlorination rather than merely a bulk solution property change. Under the combined effects of electrostatic adsorption and fluid flow, Cl⁻ released from the tandem redox system migrate more readily towards the anode for conversion. Previous studies suggest that anodic chloride activation proceeds through interfacial enrichment/adsorption of Cl⁻ followed by the formation of adsorbed chlorine intermediates (e.g., Cl or OCl). Depending on the interfacial binding strength and local reaction environment, these intermediates may either evolve toward Cl_2_/HOCl or further generate chlorine radicals. Therefore, in the present system, the released Cl⁻ is proposed to be activated on the RuO_2_/Ti anode through adsorbed chlorine intermediates, with a radical-dominated RCS pathway favored under the present conditions^48,49^.

To further verify the generation of RCS and confirm its species type, degradation experiments were conducted using different quenchers (Fig. 3c)^15,39^. The addition of NaHCO_3_ resulted in a slight decrease in degradation efficiency, indicating the presence of Cl· and possibly ·OH in the system^18^. t-BuOH acted as a quencher for H*, Cl·, and ·OH, while its reactions with ClO· and Cl₂·⁻ were negligible^15,50,51^. MeOH quenched Cl·, Cl₂·⁻, and ClO·, exhibiting particularly high quenching efficiency for Cl· and ClO·^52,53^. t-BuOH demonstrated slightly stronger quenching effects than methanol, indicating that ClO· and Cl₂·⁻ do not play a primary role. In addition, the reaction rate constant of acetate with Cl· is significantly higher than that of acetone with Cl·, whereas both compounds exhibit comparable reactivity toward ·OH^39^. As shown in Fig. 3c, acetate caused a much stronger inhibition of degradation than acetone, highlighting the important role of Cl· in the oxidation process. These results indicate that released Cl^-^ in the solution were converted into RCS for oxidation, with Cl· being the primary contributor throughout the oxidation process.

EPR provided a more direct confirmation of the presence of Cl·. ɑ-Phenyl-N-tert-butylnitrone (PBN), as the capture agent for Cl·, can simultaneously produce distinctly different signals of ·OH and Cl·^39,54^. As shown in Fig. 3d, an eight-peak signal with an intensity ratio of 1:1:2:2:2:1:1 could be found which was attributed to Cl· radical (aN = 12.11 G, aCl = 6.15 G)^39,55^. The peak intensity of the Cl· radicals showed a obviously enhancement with the reaction proceeds from 10 min to 120 min. In the tandem reaction, more Cl^-^ was released into the solution, increasing the concentration of Cl^-^. As a result, more Cl· could be excited. The quenching experiments and EPR results clearly confirmed the involvement of Cl· in the tandem redox reaction for 2,4,6-TCP degradation. Further analysis of free chlorine in the electrolyte was around 0.2 mg/L in the cascade reactor (Fig. 3e). This suggests that a small amount of non-radical active chlorine species also coexisted in the reaction system. Moreover, this low free chlorine concentration was due to the rapid consumption of Cl· and other RCS during the degradation process, where they were quickly involved in reactions with intermediates. It indicates that the chlorine species were continuously recycled and play a dominant role in the oxidation process.

In this cascade chlorine cycling electrode reactor, a tandem redox reaction was implemented to achieve the removal of 2,4,6-TCP. Firstly, an H*-dominated ECH process was occurred on the PdCo/CP cathode, converting it into more easily degradable organic compounds. The released Cl⁻ was subsequently converted at the RuO_2_/Ti anode into RCS, which dominated by Cl· and contained minor amounts of ClO·, Cl₂·, and free chlorine. These RCS then further oxidized the dehalogenated intermediates, enabling the complete removal of 2,4,6-TCP.

To further evaluate the applicability of the tandem system, several representative non-phenolic chlorinated organic pollutants and an aliphatic chlorinated compound, including trichloroethylene (TCE), atrazine (ATZ), 4-chloroaniline (4-CA), chloramphenicol (CAP), and diclofenac sodium (DCF-Na), were selected for investigation. The results showed that the system maintained high degradation efficiencies toward these substrates, whereas the TOC removal efficiencies varied considerably (Supplementary Fig. 11). This difference suggests that pollutant degradation and deep mineralization were not fully synchronous, and that mineralization depends not only on parent-compound conversion, but also on the amount of releasable chloride and the structural complexity of the target molecules. For substrates capable of releasing more Cl⁻ during cathodic dechlorination, more precursors could be supplied for subsequent anodic generation of RCS, thereby promoting deeper oxidation and mineralization. These results further support the tandem mechanism in which cathodic dechlorination-released Cl⁻ is reutilized at the anode to generate RCS for subsequent oxidative mineralization.

To semi-quantitatively evaluate the contribution of the RCS-mediated oxidation process to the overall performance enhancement of the system, the composition of the electrolyte after the reduction-only reaction was first analyzed, which mainly contained Cl⁻, 2,4,6-TCP, and its representative dechlorinated intermediate, phenol. Based on this composition, a simulated electrolyte was further prepared, and oxidation-only experiments were conducted in the presence and absence of Cl⁻ to investigate the role of RCS-mediated oxidation in the overall system performance. As shown in Fig. 3f, in the presence of Cl⁻, the removal efficiencies of 2,4,6-TCP and phenol reached 82.8% and 98.3%, respectively, and the TOC removal efficiency reached 76.72%, which was comparable to the mineralization performance of the redox tandem system. In contrast, in the absence of Cl⁻, the TOC removal efficiency was only 12.46%. This result indicates that the presence of Cl⁻ provided an additional 60.4 percentage points of TOC removal enhancement during the subsequent oxidation stage, strongly suggesting that Cl⁻-derived RCS play a dominant role in deep oxidation and mineralization. Combined with the reduction-only results, it can be inferred that sequential dechlorination mainly contributes to C–Cl bond cleavage, precursor transformation, and detoxification, whereas RCS-mediated oxidation is primarily responsible for the subsequent ring opening and deep mineralization. Therefore, the performance enhancement of the tandem system arises from the sequential synergy between these two processes, with RCS-mediated oxidation being the key contributor to TOC removal improvement. With the addition of external Cl⁻ to the electrolyte, the TOC removal efficiency further increased from 73.97% to 92.45% as the chloride concentration increased (Supplementary Fig. 12). This result suggests that a higher Cl⁻ concentration provides more available precursors for RCS generation, thereby promoting subsequent deep oxidation and enhancing the mineralization efficiency.

To further clarify the relative contributions of different reactive species during the oxidation process, both scavenging experiments and probe-based kinetic analysis were employed. As shown in Fig. 3g, the addition of nitrobenzene (NB), a quencher that is highly reactive toward ·OH but has negligible effect on RCS, resulted in only an 8.7% decrease in degradation efficiency^56^. In contrast, the addition of MeOH, which can quench both ·OH and Cl·, caused a much larger decrease of 40.6%. This result indicates that RCS plays a more dominant role than ·OH in the oxidation step. Furthermore, NB, BA, CBZ, and DMOB were used as probe compounds to semi-quantitatively evaluate the apparent steady-state concentrations of ·OH, Cl·, ClO·, and Cl₂·⁻, respectively, as summarized in Table 1^39^. In the oxidation system without Cl⁻ and 2,4,6-TCP, no obvious RCS signal was detected. In contrast, in the Cl⁻-containing system, the apparent steady-state concentration of Cl· reached 6.37 × 10^−13^ M, which was markedly higher than that of ·OH, indicating the dominant role of Cl· in this system. The relative contribution analysis derived from probe kinetics (Fig. 3h) further showed that Cl· accounted for the major fraction of the oxidation process, while ClO· made a secondary contribution, and the roles of Cl₂·⁻ and ·OH were comparatively minor. Taken together, these results demonstrate that the oxidation process in the tandem system was mainly driven by RCS, among which Cl· was the dominant reactive species.

**Table 1:** The calculated steady-state concentrations of ·OH, Cl·, ClO·, and Cl_2_·⁻

	·OH (10^−16^ M)	Cl· (10^−13^ M)	ClO· (10^−13^ M)	Cl_2_·^-^ (10^−14^ M)
Without Cl^-^	8.92	0.00	0.00	0.00
With Cl^-^	2.11	6.37	1.59	4.88

### Visualization of the degradation performance

To visually track the migration of Cl⁻ released from cathode to the anode and its subsequent conversion into RCS, LSCM was performed using a customized electrochemical imaging platform^57^. By analyzing the fluorescence signals from electrolytes containing different Cl⁻ concentrations (including 0.05 mM fluorescent dye MQAE), the relationship between Cl⁻ concentration and fluorescence intensity was determined. As shown in Supplementary Fig. 13, fluorescence intensity gradually decreased with increasing Cl⁻ concentration. To further illustrate the correlation between Cl⁻ concentration and fluorescence intensity, a standard curve was fitted using Python (Supplementary Fig. 14). Fluorescence intensity exhibited a linear relationship with Cl⁻ concentration, with a correlation coefficient R² = 0.99. This standard curve enables visualization of fluorescence images over time, facilitating observation of reactions on the electrode surface.

**Fig. 4:**
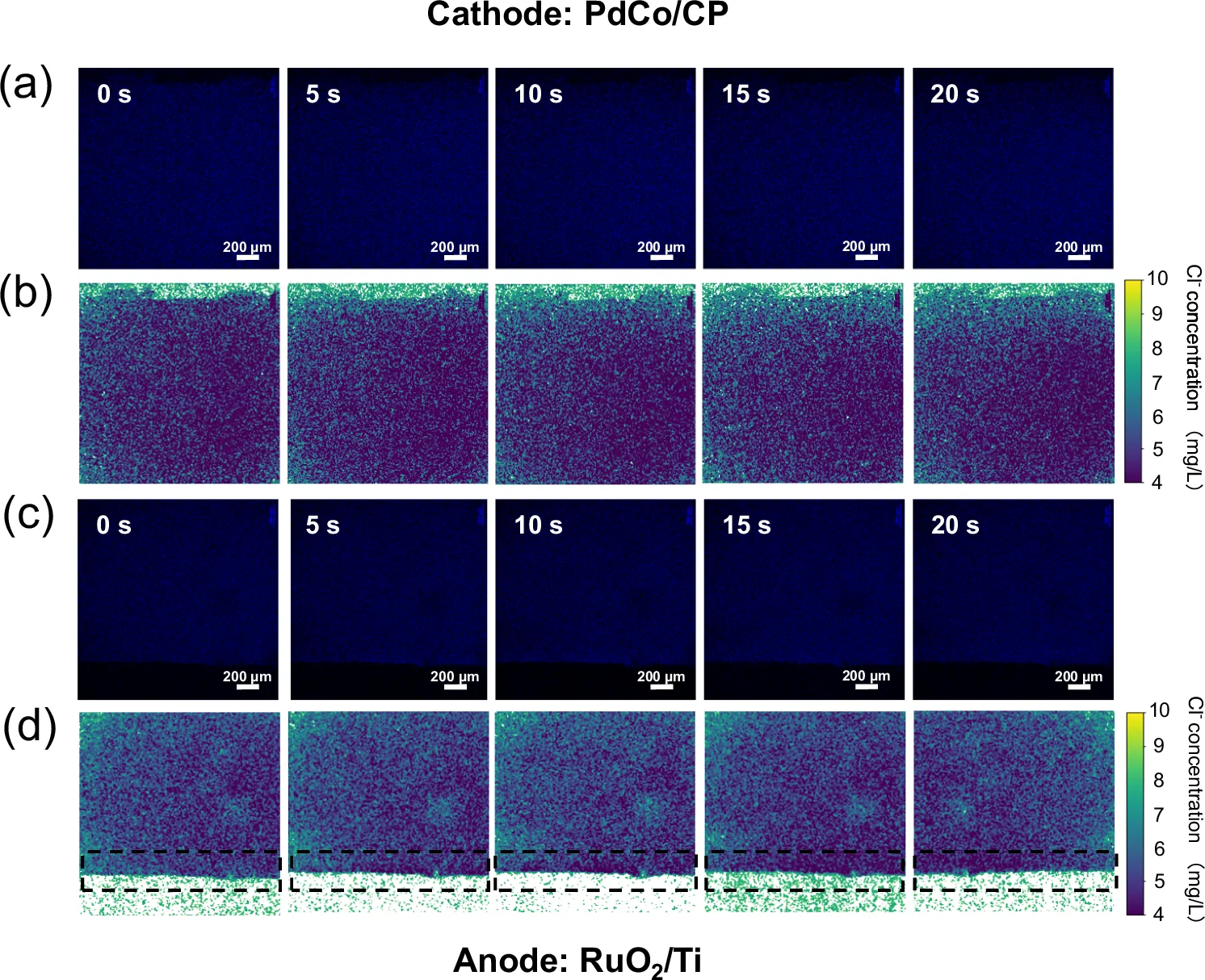
Chloride ion concentration imaging using LSCM. **a**, **c** LSCM images at the interface between **a** the PdCo/CP cathode and **c** the RuO_2_/Ti anode. **b**, **d** Visualization of chloride ion concentration of **b** the PdCo/CP cathode and **d** the RuO_2_/Ti anode (the black dashed boxes highlight the surface of anode to show the change clearly).

For better visualization and based on the data shown in Fig. 4a, the electrolyte collected 30 min into reaction was tested for Cl⁻ concentration. After adding 0.05 mM MQAE to the electrolyte, voltage was applied to observe fluorescence changes. As shown in Fig. 4a, b, on the PdCo/CP cathode surface, the fluorescence intensity was low at the onset of the reaction, indicating a high concentration of Cl⁻. This confirms that dechlorination reaction was occurring at the cathode. After 10 s, the fluorescence intensity showed little regional variation, indicating continuous migration of Cl⁻ toward the anode under the applied electric field and thus confirming active Cl⁻ transport. Between 0 and 20 s, an overall decline in fluorescence intensity was observed, reflecting the accumulation of Cl⁻ due to the ongoing dechlorination process at the cathode. This trend also explains why the RCS spectrum becomes more pronounced over time in EPR testing as the increased generation of Cl⁻ leads to heightened production of RCS (Fig. 3d).

In Fig. 4c, d, the fluorescence intensity near the RuO₂/Ti anode was initially low and remained generally weaker than that near the cathode, indicating migration and enrichment of Cl⁻ toward the anode under the applied electric field. This interfacial accumulation also implied that Cl⁻ was not merely transported to the anode surface, but was further electrochemically consumed there. Previous studies have shown that under anodic polarization, interfacial Cl⁻ can dynamically adsorb on active sites and be converted into adsorbed chlorine intermediates (e.g., M–Cl, Cl, or OCl), which subsequently evolve into RCS. Therefore, the local decrease in Cl⁻ concentration observed at the anode surface during 5–15 s was reasonably attributed to its interfacial oxidation and conversion into RCS, rather than simple redistribution in the electrolyte^58^. In the 5–15 s, the fluorescence intensity at the anode surface increased markedly corresponding to the local conversion of Cl⁻ into RCS, which in turn lowered the nearby Cl⁻ concentration. Over time, the continuous supply of Cl⁻ to the anode and the sustained RCS generation enabled effective oxidative degradation of contaminants, leading to complete mineralization. Importantly, Cl⁻ can be continuously recycled within this process, supporting a green process that eliminates the need for external oxidants and reduces energy consumption. LSCM visualization of Cl⁻ distribution provides direct evidence for RCS formation and confirms that the chlorine originates from H*-mediated abstraction from 2,4,6-TCP.

### Process simulation of redox reaction system

**Fig. 5:**
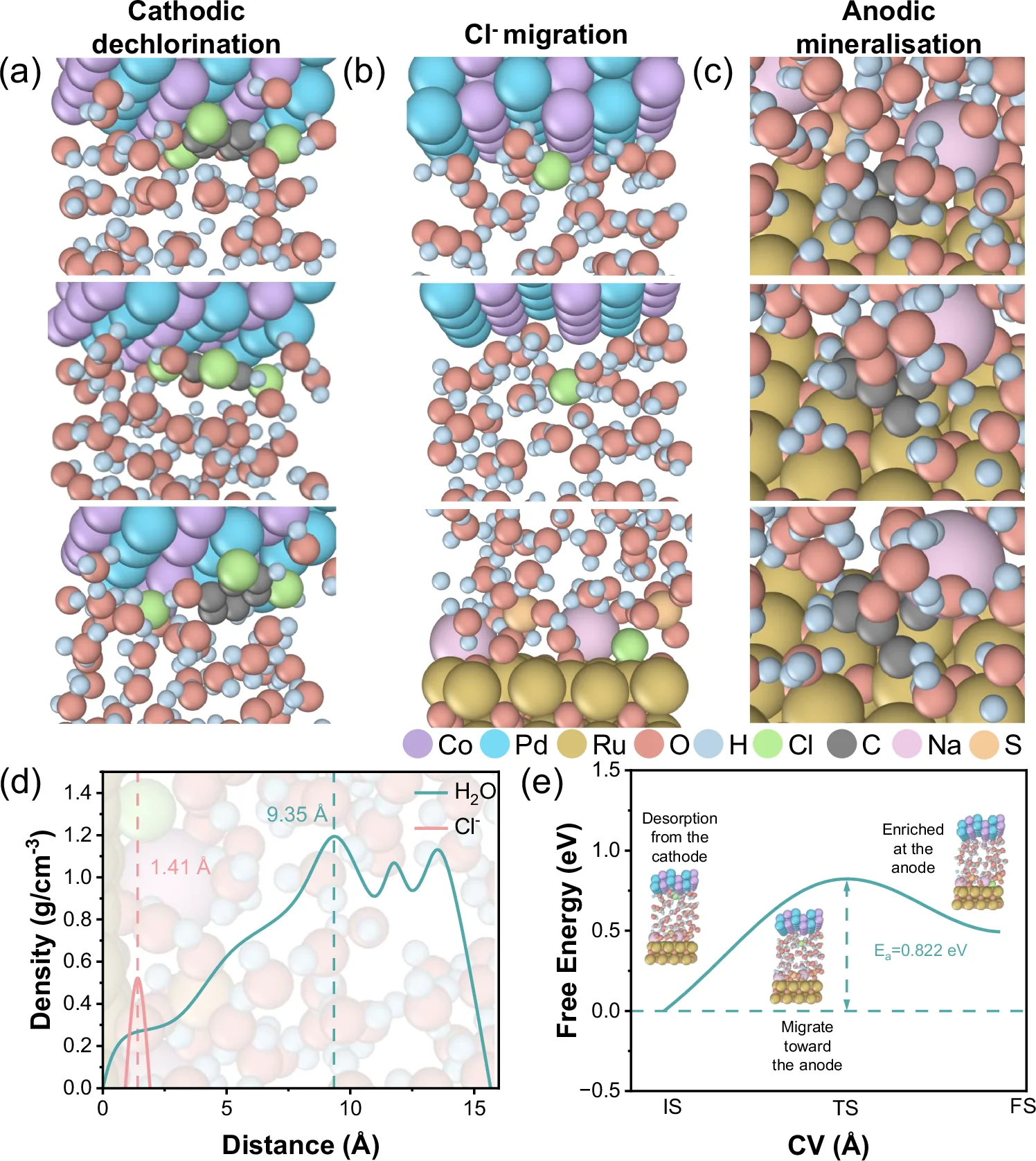
AIMD simulation of the reaction process within the system. **a**–**c** Models of the **a** dechlorination process, **b** Cl^-^ migration, and **c** oxidative ring-opening process through AIMD. **d** Concentration distribution profiles of Cl^-^ and H_2_O along the surface normal direction. **e** The energy curve of Cl^-^ migration from the cathode surface to the anode surface, along with structural changes in the initial, intermediate, and final states.

Ab-initio molecular dynamics (AIMD) simulations were conducted to elucidate the molecular behavior and dynamic reaction pathways at the electrode–electrolyte interface^59^. Figure 5a presents successive snapshots of the 2,4,6-TCP dechlorination process occurring at the PdCo/CP interface. Initially, 2,4,6-TCP molecules adsorb onto the active sites of the PdCo/CP cathode. The highly hydrophilic cathode surface facilitates water electrolysis, generating adsorbed H*. These H* then attack the C–Cl bond, leading to its cleavage. Following dechlorination, the resulting products desorb from the electrode surface, releasing Cl⁻ ions that diffuse into the bulk electrolyte and regenerating the active sites for subsequent catalytic cycles. Figure 5b illustrates the migration trajectory of Cl⁻ from the cathode to the anode under the combined influence of the electric field and fluid flow. Consistent with the LSCM results in Fig. 4a, b, Cl⁻ generated at the cathode accumulates and migrates toward the anode, where it is progressively converted into RCS. This process not only drives further oxidation reactions but also helps mitigates cathode poisoning by Cl⁻. As shown in Fig. 5c, the dehalogenated intermediates are transported to the anode via fluid flow and adsorbed onto RuO₂/Ti surface, highlighting the role of the cascade reactor in coordinating the reaction sequence and accelerating subsequent steps. The enrichment and conversion of Cl⁻ to RCS at the anode align with the sharp decrease in Cl⁻ concentration near the cathode observed by LSCM (Fig. 4c, d). Figure 5c depicts the RCS-mediated oxidative ring-opening, where RCS attacks electron-rich sites on the aromatic ring, breaking it down into small organic molecules.

Figure 5d illustrates the distance of Cl⁻ and H_2_O from the anode surface along the normal direction. As a specifically adsorbable reactant, Cl⁻ is located much closer to the anode, with a preferred distance of approximately 1.41 Å, indicating its enrichment within the inner Helmholtz layer. In contrast, H_2_O molecules are distributed farther from the surface, mainly in the outer Helmholtz layer and diffusion layer, with distances predominantly exceeding 9.35 Å. This distinct interfacial conFiguration suggests that Cl⁻ has a greater probability of directly interacting with anodic active sites than H_2_O, thereby favoring the CER over the competing OER. As a result, the interfacial enrichment of Cl⁻ in the Helmholtz layer facilitates the generation of RCS for subsequent oxidation reactions. Figure 5e depicts the energy changes during Cl⁻ migration under electric field. The energy barrier from the initial state to the transition state is 0.822 eV, whereas no additional energy input is required from the transition state to the final state. This indicates that Cl⁻ migrates smoothly toward the anode and accumulates under the combined influence of the flow field and electric field, thereby establishing favorable conditions for RCS generation.

The AIMD simulations corroborate the LSCM observations, validate the operational efficacy of the cascade reactor, and confirm the mechanistic feasibility of the redox tandem strategy, providing atomistic-level insights into the interfacial reaction process.

### The degradation pathways and mechanisms

The degradation pathways and mechanisms were further analyzed by UPLC-MS. As shown in Supplementary Figs. 15–17, the UPLC-MS analysis were conducted during the reaction of reduction only, oxidation only, and redox synergy processes (Supplementary Figs. 15–17). After 5 min of reduction alone, the products included dichlorophenol (2,4-DCP, 2,6-DCP) formed by the removal of one Cl atom, chlorophenol (2-CP, 4-CP) resulting from the removal of two Cl atoms, and phenol following dechlorination (Supplementary Fig. 15). After 5 min of oxidation only, the main products were 2,4-DCP and 2,6-DCP, chlorinated BQ compounds, and chlorinated small-molecule carboxylic acids produced by oxidative ring-opening (Supplementary Fig. 16). In contrast, the intermediate products generated during the redox synergy process were different. Along with phenol detected after complete dechlorination, BQs and small-molecule carboxylic acids were also identified (Supplementary Fig. 17). These results indicate that oxidation-only and redox tandem follow distinct transformation sequences: oxidation-only directly oxidizes highly chlorinated substrates, whereas the tandem process first removes chlorine substituents through cathodic hydrodechlorination and then oxidizes the dechlorinated intermediates.

**Fig. 6:**
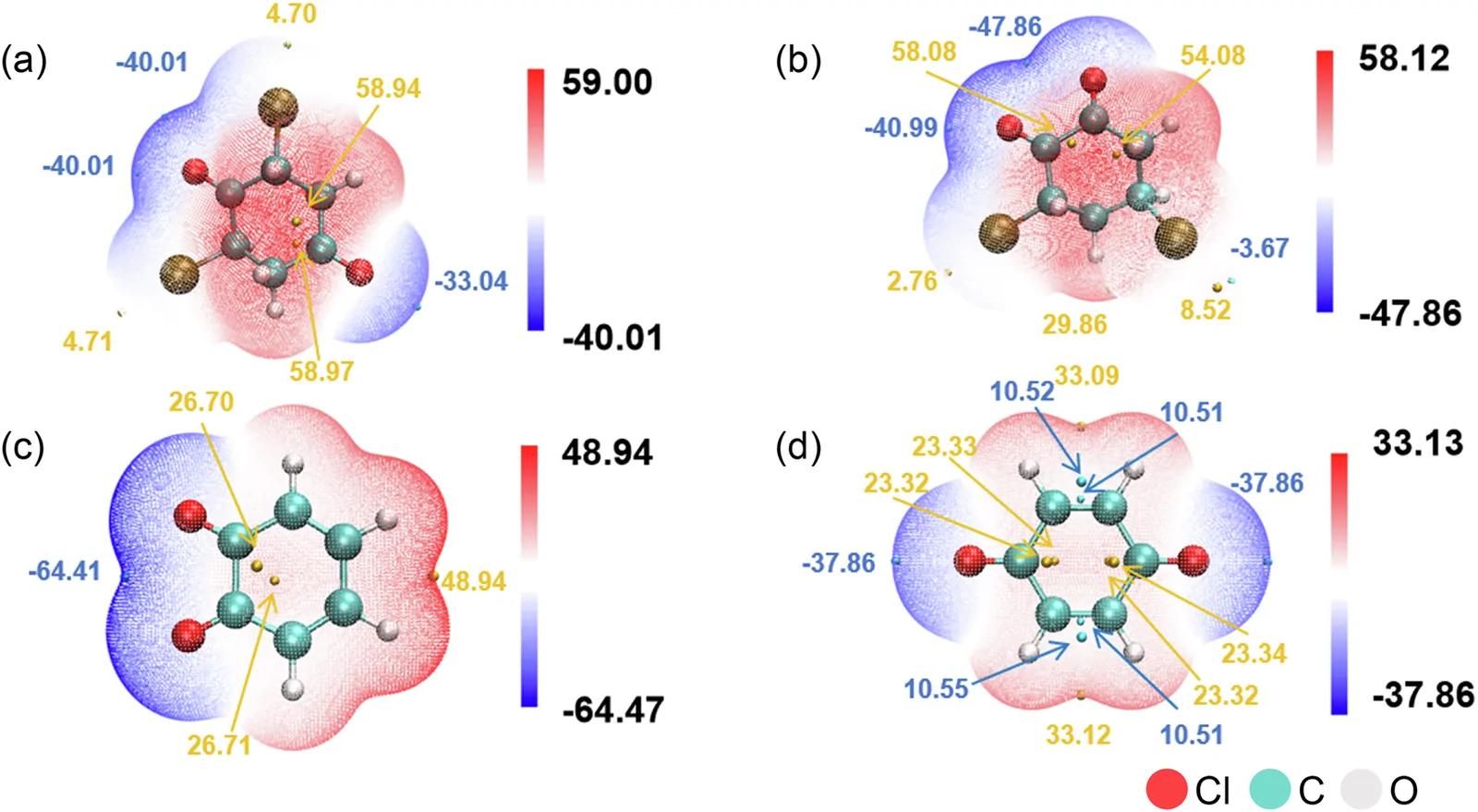
ESP analysis of the intermediate product. **a**–**d** The 3D ESP mapping for **a** dichloro-p-BQ, **b** dichloro-p-BQ, **c** o-BQ, and **d** p-BQ.

The electrostatic potential (ESP) of the chlorinated BQ intermediate generated from oxidation only and the BQ intermediate derived from the redox tandem process was analyzed to evaluate the role of chlorine substitution in influencing the oxidative ring-opening pathway. As shown in Fig. 6a, b, the ESP profiles of dichloro-BQ span a wide range, exhibiting localized positive regions alongside weakly negative areas. This distribution reflects the strong electron-withdrawing effect (−I) of chlorine atoms, which significantly depletes the π-electron density of the aromatic ring, rendering the overall molecular framework electron-deficient. In contrast, the ESP maps of p-BQ and o-BQ (Fig. 6c, d) exhibit uniformly moderate to highly negative values, indicating the presence of electron-rich systems. Since RCS act as mild electrophiles that preferentially target electron-rich sites during oxidation, p-BQ and o-BQ provide favorable locations for electrophilic attack, facilitating efficient ring cleavage. As a result, the redox tandem strategy achieves enhanced degradation and TOC removal efficiency, enabling complete mineralization of organic pollutants and further promoting reaction progression.

**Fig. 7:**
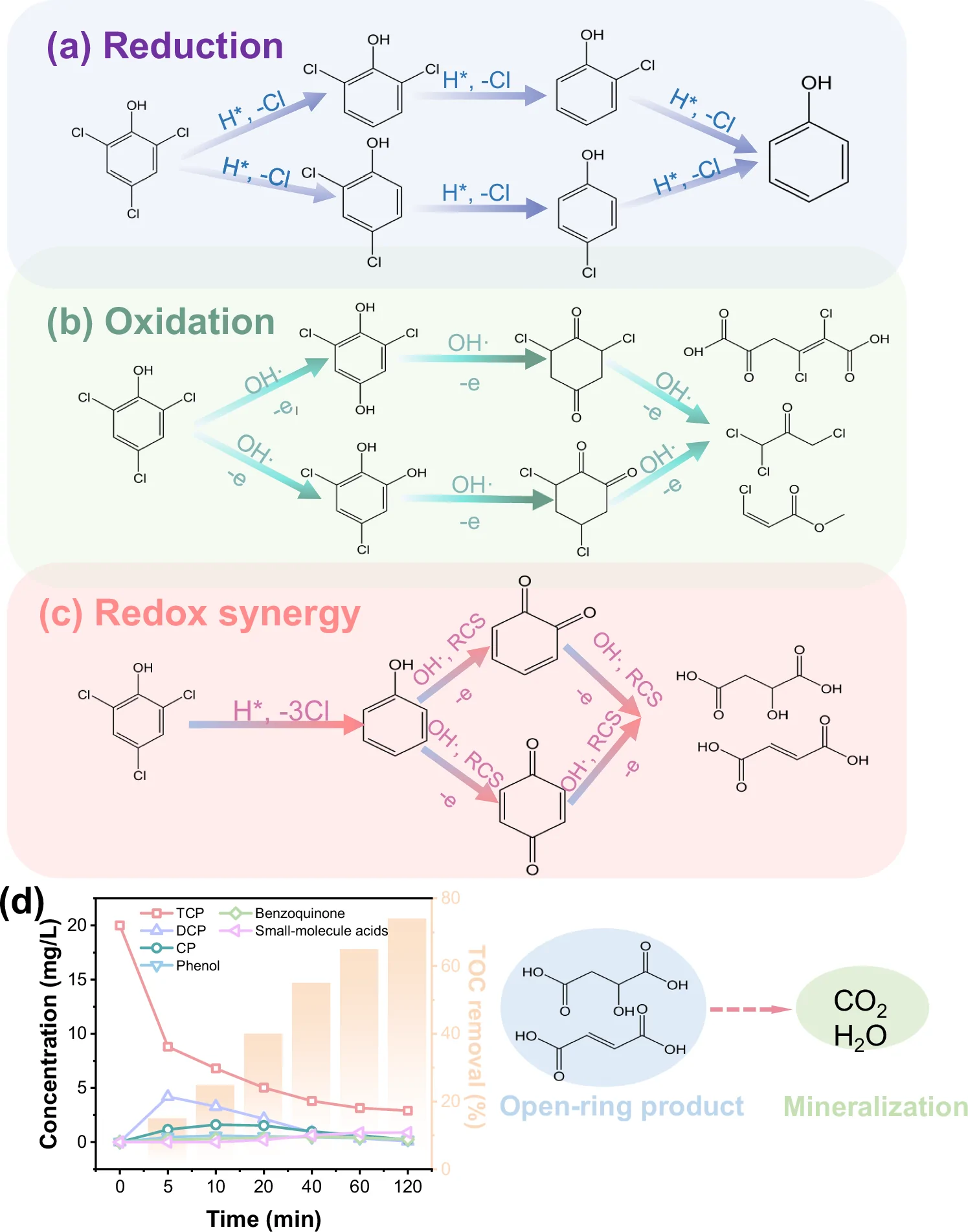
Degradation pathways of 2,4,6-TCP in different systems. **a**–**c** The schematic diagram of reaction pathways of PdCo/CP cathode-RuO_2_/Ti anode for 2,4,6-TCP degradation via **a** reduction only, **b** oxidation only, and **c** redox tandem. **d** Concentration changes of intermediate products and TOC removal in the redox tandem system.

Based on the above discussion, the degradation pathways of 2,4,6-TCP for reduction only, oxidation only, and redox synergy processes were inferred. Figure 7a illustrates the reaction pathway under reduction only, where 2,4,6-TCP first loses one Cl atom through the action of H*. It proceeds along two pathways—one involving initial Cl removed from the ortho position and another from the para position. Subsequently, further dechlorination formed 4-CP and 2-CP, respectively, until complete dechlorination produces phoenol as the final product^22^. Because this pathway mainly removes chlorine substituents but does not efficiently cleave the aromatic ring, mineralization remains incomplete, and the reaction terminates predominantly at phenolic intermediates. Figure 7b presents oxidation only, in which OH· radicals first attacks the chlorine atom at the ortho or para position. Then the intermediate is oxidized to form chlorinated BQ, which undergoes further oxidative ring-opened to form chlorinated carboxylic acid-containing organic compounds. Because oxidation occurs before sufficient dechlorination in the oxidation-only pathway, many intermediates still retain chlorinated aromatic structures, which are less favorable for deep oxidation and therefore more likely to accumulate during the reaction. Figure 7c depicts the reaction pathway of the redox synergy process. Here, 2,4,6-TCP is completely dechlorinated to phenol via electrocatalytic hydrogenation to form phenol. Phenol is then oxidized by ·OH and RCS to ortho- and para-benzoquinone. These quinoes are further oxidized to maleic acid and fumaric acid, and finally completely mineralized to H₂O and CO₂. In contrast, the tandem process reconstructs the pathway into a dechlorination-first route, thereby suppressing the formation and persistence of toxic chlorinated oxidation intermediates.

To further clarify the relationship between TOC removal and the oxidation process, the temporal evolution of TOC and the concentrations of the major intermediates were investigated. As shown in Fig. 7d, at the early stage of the reaction, the concentrations of dechlorination products, including DCP, CP, and phenol, initially increased, indicating that the cathodic reduction process first promoted the stepwise dechlorination of 2,4,6-TCP to form lower-chlorinated products and phenol. Subsequently, with the continuous generation of RCS, these dechlorinated intermediates were further oxidized, leading to the gradual formation of more oxidized products such as BQ and low-molecular-weight organic acids. As the reaction proceeded further, the concentrations of these oxidation intermediates also gradually decreased, accompanied by a continuous decline in TOC, indicating that the organics were further oxidized and eventually driven toward mineralization to form CO_2_ and H_2_O.

### The toxic simulation of intermediate product

The toxicity of intermediates generated from reduction-only, oxidation-only, and redox tandem processes in 2,4,6-TCP degradation were evaluated toward fish, Daphnia, and green algae by toxicity analysis software ECOSAR.

As depicted in Supplementary Fig. 18a, the LC_50_ values progressively increased with the sequential removal of chlorine atoms, indicating a stepwise reduction in toxicity across all three test organisms. In the oxidation-only process, a considerable amount of highly toxic intermediates was identified, including chlorinated quinones and chlorinated aldehydes. Owing to the presence of chlorine on the aromatic ring, the ring-opening products exhibited even higher toxicity than their precursors prior to ring cleavage (Supplementary Fig. 18b). In contrast, as shown in Supplementary Fig. 18c, intermediates produced via sequential dechlorination followed by ring-opening exhibited a toxicity reduction by several orders of magnitude, with values continuing to decline without any observable toxicity peak. These findings underscore the synergistic effect of the redox tandem strategy. The initial dechlorination of 2,4,6-TCP effectively diminishes the primary toxicity, while subsequent electrooxidation thoroughly mineralizes organic constituents, thereby minimizing the accumulation of hazardous byproducts. Importantly, the chlorine-free oxidation products demonstrate significantly lower toxicity compared to their chlorinated counterparts, ensuring the ecological safety of the treated effluent.

## Discussion

In summary, this study demonstrates a cascade electrochemical reaction system based on a redox tandem mechanism, in which Cl removed via ECH is effectively converted into RCS for secondary oxidation, enabling the complete degradation and mineralization of 2,4,6-TCP. By matching a RuO₂/Ti anode with a PdCo/CP cathode under optimized applied voltage, 2,4,6-TCP is first dechlorinated to phenol at the cathode, which is more susceptible to further oxidation. The released Cl⁻ migrates to the anode, where it is electrochemically transformed into RCS, facilitating deep oxidation of the dechlorinated intermediates. This strategy not only overcomes the inherent limitations of conventional electrochemical processes, such as incomplete mineralization in ECH and low efficiency in electro-oxidation of chlorinated organics, but also enables the secondary utilization of Cl while minimizing cathode poisoning. The proposed system offers a sustainable model for the efficient degradation of chlorinated organic pollutants, highlighting the potential of resource-recycling electrochemical technologies for environmental remediation.

## Methods

### Chemicals and materials

Potassium tetrachloropalladate (K_2_PdCl_4_), sodium chloride (NaCl), sodium sulfate (Na_2_SO_4_), trichloroethylene (TCE), atrazine (ATZ), 4-chloroaniline (4-CA), chloramphenicol (CAP), diclofenac sodium (DCF-Na), sodium bicarbonate (NaHCO_3_), acetate, acetone, sulfuric acid (H_2_SO_4_) and sodium hydroxide (NaOH) were purchased from Aladdin Reagent (Shanghai, China). Cobalt (II) nitrate hexahydrate (Co(NO_3_)_2_·6H_2_O), tert-butanol (t-BuOH), 2-methylimidazole (2-MI), and nitric acid (HNO_3_) were purchased from Sigma-Aldrich (Shanghai, China). Methanol (MeOH), acetic acid (CH_3_COOH), 5,5′-dimethyl-1-pirroline-N-oxide (DMPO), and N-tert-Butyl-α-phenylnitrone (PBN) were purchased from Sinopharm Chemical (Beijing, China). Methanol (MeOH, HPLC grade) was purchased from Merck KGaA (Germany). Carbon paper (CP, HCP030N) was supplied by Hesen Co. Ltd. (Shanghai, China). In order to enhance its hydrophilicity, the carbon paper (2 × 2 cm) was immersed in HNO_3_ for 24 h and rinsed with deionized water, then dried in a vacuum oven at 60 °C overnight before use. The Ti/ RuO_2_ electrode was supplied by Suzhou Shuer Tai Industrial Technology Co, Ltd. (Suzhou, China). The proton exchange membrane was Nafion 115 and was purchased from Chemours (USA). 2,4,6-Trichlorophenol (2,4,6-TCP, purity>98%) was procured from Meryer Co., Ltd. (Shanghai, China). All chemicals used in the experiments were analytical grade and solutions were prepared using ultrapure water (18.2 MΩ/cm) (Miaozhiyi, China).

### Preparation of PdCo/CP cathode

The synthesis of PdCo/CP cathode was through previous method^18^. Firstly, immerse the pretreated CP in 40 ml methanol solution of Co(NO_3_)_2_·6H_2_O (1.16 g), and then stirred for 1 h. 2-methylimidazole (2-MI, 3.3 g) was dissolved in methanol (40 mL) and poured into the above solution with stirring for 12 h. Thereafter, K_2_PdCl_4_ was added into solution in which the molar ratio of Pd to Co was 1:1 and stirred for 12 h. 20 ml NaBH_4_ aqueous solution (1 mol/L) was then added and the mixed solution stirred for 15 min. The obtained CP was washed three times with ethanol and dried at 60 °C for 12 h, recorded as Pd/ZIF-67/CP. The Pd/ZIF-67/CP was placed at 800 °C for 3 h under flowing N_2_ gas, and then naturally cooled to room temperature. The obtained electrode was denoted as PdCo/CP.

### Characterization

The scanning electron microscopy (SEM, ZEISS GeminiSEM 360, Germany) with energy dispersive X-ray spectroscopy (EDS) was used to observe the morphology and element distribution of PdCo/CP and CP. Transmission electron microscopy and high-resolution transmission electron microscopy (TEM and HRTEM, JEM ARM-200F, Japan) were employed to characterize the structure. X-ray diffraction (XRD) patterns were acquired by a BRUKER D8 advance powder X-ray with a Cu-Kα radiation diffractometer (λ = 1.5418 Å) at 40 kV and 40 mA to elucidate the structural and phase information. The chemical compositions and chemical states of the electrodes were probed by X-ray photoelectron spectroscopy (XPS, Thermo Scientific K-Alpha with Al Kα radiation at 1486.6 V, USA).

The concentrations of 2,4,6-TCP was quantified by a high-performance liquid chromatography (HPLC, e2695, Waters, USA) equipped with a C18 liquid chromatography column at a constant temperature of 25 °C. The flow rate of the mobile phase was 1 mL/min and the injection volume was 50 μL. The specific detection conditions for 2,4,6-TCP, TCE, 4-CA, CAP, DFC-Na, and ATZ are as follows.

2,4,6-TCP: The mobile phase consisted of methanol and ultrapure water with a ratio of 80:20, and a UV-vis detector at a wavelength of 245 nm.

TCE: The mobile phase consisted of acetonitrile and ultrapure water with a ratio of 50:50, and a UV-vis detector at a wavelength of 215 nm.

CA: The mobile phase consisted of methanol and ultrapure water with a ratio of 55:45, and a UV-vis detector at a wavelength of 240 nm.

CAP: The mobile phase consisted of sodium acetate and methanol with a ratio of 65:35, and a UV-vis detector at a wavelength of 445 nm.

DFC-Na: The mobile phase consisted of methanol and 0.1% acetic acid solution with a ratio of 60:40, and a UV-vis detector at a wavelength of 276 nm.

ATZ: The mobile phase consisted of methanol and ultrapure water with a ratio of 70:30, and a UV-vis detector at a wavelength of 221 nm.

The intermediates of 2,4,6-TCP reduction were analyzed by an Ultra-High Performance Liquid Chromatography-Quadrupole Time-of-Flight Mass Spectrometry (UPLC-MS, SCIEX Exion LC, USA). The samples were analyzed using the SCIEX Exion LC ultra-high performance liquid chromatography (UPLC-MS) system, with chromatographic separation performed on a Phenomenex Kinetex® C18 column (100 × 2.1 mm, 2.6 μm) equipped with a security guard column (Security Guard UHPLC C18 column), maintained at a column temperature of 40 °C. The mobile phase system consisted of 0.1% formic acid in water (v/v, solvent A) and acetonitrile (solvent B), with a flow rate of 0.4 mL/min and an injection volume of 10 μL. The temperature of the autosampler was maintained at 4 °C throughout the analysis. The detailed gradients are shown in Table S1.

Ecological structure activity relationships (ECOSAR) was used to analyze the toxicity of intermediate products after degradation through different pathways. The total organic carbon (TOC) analyzer (Elementer Vario-TOC Cube, Germany) was utilized to quantify the reduction of TOC within the reaction mixture. To calculate the energy consumption per mg of TOC removed, the following step was performed:

1 $${{{\rm{Energy}}}}\,{{{\rm{per}}}}\,{{{\rm{mg}}}}\,{{{\rm{TOC}}}}\,{{{\rm{removed}}}}\,=\frac{{{{\rm{V}}}}\times {{{\rm{I}}}}\times {{{\rm{t}}}}}{{{{\rm{TOC}}}}\,{{{\rm{removed}}}}}$$

Where E is the energy consumed (J), V is the applied voltage (V), I is the current displayed at this voltage (A), TOC removed is the difference between the initial TOC and the final TOC (mg) and t is the duration of the reaction (s).

The concentration of chloride ions after the reaction was determined by ion chromatography (IC, AQUION, Thermo Fisher, USA). Conductivity was measured using a conductivity meter (DDSJ-308F, China). Residual chlorine concentration was detected using a residual chlorine analyzer (ERUN-ST7-K6, China). The inductively coupled plasma emission spectrometer (ICP, ICAP Pro, Thermo Fisher, USA) was used to measure metal leaching.

The electrochemical measurements were carried out in a three-electrode system with an electrochemical workstation (CHI 760E, CH Instruments, China). 50 mM Na₂SO₄ and NaCl were used as electrolyte. The counter electrode was the 2 × 2 cm platinum foil and the saturated Ag/AgCl electrode was used as the reference electrode. The electrochemical performance was evaluated using linear sweep voltammetry (LSV), with a scanning range of 0.8 to 2.5 V vs. RHE and a scanning rate of 10 mV/s. Cyclic voltammetry (CV) was conducted at a scanning rate of 50 mV/s from −1.5 to 0.8 V vs. Ag/AgCl. EPR spectra were recorded on a Bruker EMXplus-9/12 spectrometer. PBN (100 mM) and DMPO (100 mM) were employed as spin-trapping agents for reactive chlorine species (RCS) and H*, respectively. The trapping agent was first introduced into the electrolyte before electrolysis. For H* analysis, the sample was taken after 5 min of reaction and immediately subjected to EPR measurement. For Cl· analysis, samples were collected at predetermined reaction intervals for EPR detection.

### Cascade chlorine cycling electrode reactor and 2,4,6-TCP degradation experiments

The degradation experiment of 2,4,6-TCP was carried out in a cascade chlorine cycling electrode reactor (Fig. S1a). The as-prepared PdCo/CP electrode was used as the cathode and the RuO_2_/Ti was utilized as anode. The two electrodes were separated by an aqueous filter membrane and the outer layer of the device was fixed with an acrylic glass plate. The reactor was connected to an adjustable DC-regulated power supply (HSPY-120-01, China). In the degradation experiment, the individual degradation effects of reduction and oxidation were elucidated by replacing the anode and cathode with carbon paper (CP), respectively. The aqueous filter membrane was substituted with a proton exchange membrane to investigate the individual degradation contributions of the anode and the cathode chambers. The detailed assembly of cascade chlorine cycling electrode reactor was illustrated in Fig. S1a. In the degradation experiment, 100 mL solution, which contained 2,4,6-TCP (initial concentration 20 mg/L) and Na_2_SO_4_ electrolyte (50 mM), was continuously upflow through the reactor from cathode to anode, and then returned to the beaker (Fig. S1b). The initial pH was adjusted by 0.1 mM H_2_SO_4_ and 0.1 mM NaOH. Based on previous studies of the cathodic reduction process, pH 4 was selected as the reaction condition in this study^18^. The electrolyte was aerated with N_2_ for 30 min before the experiment. During the experiment, 1 mL liquid sample was collected from the beaker at certain intervals for further quantification. The initial concentration of 2,4,6-TCP was 20 mg/L in all degradation experiments unless otherwise specified. All degradation experiments were repeated three times to ensure the reliability of the experimental results.

### Visualization monitoring Cl^-^ concentration during the dechlorination conversion process

To explore the generation and migration of Cl^-^ during the dechlorination process occurred at cathode and its conversion near anode. A custom-made electrochemical cell was fabricated with a 20.0 mm diameter confocal culture dish. This electrolyte solution was reacted for 30 min within the cascade reactor system, with 0.05 mM N-(Ethoxycarbonylmethyl)-6-methoxyquinoline bromide (MQAE) added prior to testing. Cell morphology and viability were monitored using a Nikon A1 HD25 confocal microscope (equipped with an HP Apochromat TIRF 10XC objective lens, Nikon Corporation, Japan). Fluorescence images of the cathode surface were acquired using a confocal laser-scanning microscope (λ = 488 nm, λ = 520 nm) and saved as 16-bit grayscale PNG files with a 200 µm embedded scale bar. Raw images were denoised with a 3 × 3 Gaussian filter (σ = 1 pixel) and an Otsu automatic threshold was applied to mask the electrode region; pixels below the threshold were set to NaN. Images were captured using NIS-Elements Ar software (Nikon Corporation, Japan).

All operations were automated in Python 3.13 using scikit-image. A five-point linear calibration curve (2, 4, 6, 8, and 10 mg/L) was constructed from standard solutions imaged under identical conditions. Electrode-region intensities were linearly mapped to the concentration range 6.0–6.5 mg/L using a fitted equation (R² > 0.99). Concentration at each pixel was calculated. Each Figure includes a 200 µm white scale bar and a color bar.

Based on previous studies, probe-based kinetic experiments were carried out under oxidation-only conditions to semi-quantitatively evaluate the relative contributions of different reactive species during the oxidation step^39^. A Cl^-^-free blank system and a Cl^-^-containing target system were established using 50 mM Na_2_SO_4_ as the supporting electrolyte, with the Cl^-^ concentration in the target system set to that measured in the post-reaction electrolyte of the reduction-only system. All other reaction conditions were kept identical to those used in the corresponding oxidation experiments. Terephthalic acid (TA, 0.5 mM) was used as the probe for ·OH, while nitrobenzene (NB), benzoic acid (BA), 1,4-dimethoxybenzene (DMOB), and carbamazepine (CBZ) were employed as stepwise discriminative probes for ·OH, Cl·, ClO·, and Cl_2_·⁻, respectively, each at an initial concentration of 50 μM. Each probe was tested individually and was not co-present with the target pollutant. Their concentrations were determined by liquid chromatography, and the specific analytical method was adopted with reference to previous studies^60^. Aliquots were collected at predetermined time intervals, filtered, and then subjected to quantitative analysis. The apparent first-order rate constant, k_obs_, for each probe was obtained by linear fitting according to:

2 $${{\mathrm{ln}}}\left(\frac{{{{{\rm{C}}}}}_{{{{\rm{t}}}}}}{{{{{\rm{C}}}}}_{0}}\right){=-{{{\rm{k}}}}}_{{{{\rm{obs}}}}}{{{\rm{t}}}}$$

t denoted the reaction time, C_t_ denoted the concentration at that time, and C_0_ denoted the initial concentration of the probe.

In the Cl⁻-free blank system, the apparent steady-state concentration of ·OH was first estimated from the apparent rate constant of TA:

3 $${[\cdot {{{\rm{OH}}}}]}_{{{{\rm{SS}}}}}=\frac{{{{{\rm{k}}}}}_{{{{\rm{TA}}}}}}{{{{{\rm{k}}}}}_{{{{\rm{TA}}}},\cdot {{{\rm{OH}}}}}}$$

where k_TA,·OH_ was the second-order reaction rate constant between TA and ·OH (4.4 × 10^9 ^M^−1^ s^−1^)^61^ and k_TA_ was the measured rate constant for TA oxidation in the blank system.

Subsequently, the decay rates of NB, BA, DMOB, and CBZ measured in the blank system were used to subtract the non-radical loss term, k_non-radical_, for each probe (Tables S4 and S5). In the Cl⁻-containing target system, the apparent rate constants of NB, BA, DMOB, and CBZ were further used in a stepwise subtraction manner to derive [·OH]_SS_, [Cl·]_SS_, [ClO·]_SS_, and[ClO_2_·^-^]_SS_. Specifically, NB was used to calculate [·OH]_SS_. BA was used to determine [Cl·]_SS_ after subtracting the contribution of ·OH. DMOB was used to calculate [ClO·]_SS_ after further subtracting the contributions of ·OH and Cl·, and CBZ was finally used to determine [ClO_2_·^-^]_SS_ after subtracting the contributions of the first three species. The results are summarized in Table 1. Since this method is based on apparent rate constants with background correction using the blank system, the obtained values are more appropriately interpreted as semi-quantitative descriptors of the relative roles of reactive species rather than strict absolute concentrations. The contributions of each free radical are calculated by the following formula:

4 $${{\mbox{Contribution}}}_{{\mbox{i}}}=\frac{{{\mbox{k}}}_{{\mbox{i}}}}{\sum {{\mbox{k}}}_{{\mbox{radicals}}}}\times 100\%$$

i represented each free radical, and “contribution” indicated its relative contribution.

### Prediction of molecular charge structure and reaction

Investigating the molecular structure of degradation intermediates via Electrostatic Potential (ESP). Simulating the interaction effects and migration throughout the degradation process using Ab Initio Molecular Dynamics (AIMD). CP2K was employed to carry out the theoretical calculations in the framework of density functional theory (DFT). The CP2K program was used in the DFT mode. CP2K employed two representations of the electron density: localized Gaussian and plane wave basis sets. For the Gaussian-based (localized) expansion of the Kohn–Sham orbitals, we used a library of contracted molecularly optimized valence double-zeta plus polarization basis sets^62^, and the complementary plane wave basis set had a cutoff of 400 Rydberg for the electron density. The generalized gradient corrected approximation of Perdew, Burke, and Ernzerhof (PBE)^63^ was adopted to relax the geometric structures. For AIMD, the sampling of the structures of bulk solution, the canonical ensemble2 (NVT) conditions were imposed by a Nose-Hoover thermostat with a temperature of 298.15 K. The MD time step is set to 1.2 fs.

## Supplementary information

Supplementary Information

Transparent Peer Review file

## Source data

Source Data

## Data Availability

All data generated in this study have been deposited in the Zenodo database under accession code 10.5281/zenodo.20815407. The data generated in this study are provided in the Supplementary Information and Source data file (https://doi.org/10.5281/zenodo.20815407)^64^. All the raw data relevant to the study are available from the corresponding author upon request. Source data are provided with this paper.

## Code availability

The code for visualization is provided in the Zenodo database (https://doi.org/10.5281/zenodo.20815407)^64^.
