# Supplementary material for: In-situ chlorine radical generation from captured Cl- for redox synergy in electrocatalytic hydrodehalogenation
Source: Nat Commun. 2026 Sep 4;17:10543. doi: 10.1038/s41467-026-77253-1 (PMC13642356; doi:10.1038/s41467-026-77253-1)
Supplement: Supplementary file 1 [file 41467_2026_77253_MOESM1_ESM.pdf]

# In-Situ Chlorine Radical Generation from Captured $Cl^-$ for Redox Synergy in Electrocatalytic Hydrodehalogenation

Chuyan Deng[1], Yujun Zhou[1]*, Xin Cai[1], Xuan Zhang[2,3], Yunlong Wang[1], Junwen Qi[1], Zhigao Zhu[1], Yue Yang[1], Jiansheng Li[1]*

[1]School of Environmental and Biological Engineering, Nanjing University of Science and Technology, Nanjing 210094, China.

[2]IIHR-Hydroscience & Engineering, University of Iowa, Iowa City, Iowa, USA

[3]Department of Mechanical Engineering, University of Iowa, Iowa City, Iowa, USA

*Corresponding authors.

E-mail addresses: yjzhou@njust.edu.cn; lijsh@njust.edu.cn

**Table S1.** Specific parameters of UPLC-MS.

| Time (min) | A | B |
|---|---|---|
| 0 | 99 | 1 |
| 1.0 | 99 | 1 |
| 10.0 | 1 | 99 |
| 13.0 | 1 | 99 |
| 14.0 | 99 | 1 |
| 17.0 | 99 | 1 |

Phase A refers to 0.1% formic acid in deionized water and phase B refers to acetonitrile.

**Table S2.** Table of Intermediate Product Concentrations.

| Time (min) | TCP (mg/L) | DCP (mg/L) | CP (mg/L) | Phenol (mg/L) | BQ (mg/L) | Acids (mg/L) |
|---|---|---|---|---|---|---|
| 0 | 20.00 | 0.00 | 0.00 | 0.00 | 0.00 | 0.00 |
| 5 | 8.8 | 4.19 | 1.16 | 0.46 | 0.21 | 0.00 |
| 10 | 6.8 | 3.28 | 1.61 | 0.59 | 0.30 | 0.00 |
| 20 | 5.03 | 2.15 | 1.52 | 0.52 | 0.39 | 0.20 |
| 40 | 3.79 | 0.91 | 0.98 | 0.46 | 0.51 | 0.63 |
| 60 | 3.14 | 0.34 | 0.62 | 0.37 | 0.41 | 0.86 |
| 120 | 2.90 | 0.07 | 0.21 | 0.21 | 0.26 | 0.87 |

**Table S3.** Table of carbon equivalent.

| Time (min) | TCP (mg C/L) | DCP (mg C/L) | CP (mg C/L) | Phenol (mg C/L) | BQ (mg C/L) | Acids (mg C/L) | TOC (mg C/L) |
|---|---|---|---|---|---|---|---|
| 0 | 7.293 | 0.000 | 0.000 | 0.000 | 0.000 | 0.000 | 7.293 |
| 5 | 3.208 | 1.851 | 0.650 | 0.352 | 0.140 | 0.000 | 6.201 |
| 10 | 2.479 | 1.449 | 0.902 | 0.451 | 0.200 | 0.000 | 5.481 |
| 20 | 1.834 | 0.950 | 0.851 | 0.398 | 0.260 | 0.083 | 4.375 |
| 40 | 1.380 | 0.402 | 0.549 | 0.352 | 0.340 | 0.260 | 3.284 |
| 60 | 1.145 | 0.150 | 0.347 | 0.283 | 0.273 | 0.356 | 2.554 |
| 120 | 1.057 | 0.031 | 0.118 | 0.161 | 0.173 | 0.360 | 1.900 |

**Table S4.** The decay rates of TA, NB, BA, CBZ and DMOB without $Cl^-$.

| species | $k_{\cdot OH+non\text{-}radical\ pathway}$($10^{-4}$ $min^{-1}$) | $k_{\cdot OH}$ ($10^{-4}$ $min^{-1}$) | $k_{non\text{-}radical\ pathway}$ ($10^{-14}$$min^{-1}$) |
|---|---|---|---|
| NB | 3.22 | 2.09 | 1.13 |
| BA | 6.91 | 3.16 | 3.75 |
| DMOB | 4.91 | 3.75 | 1.16 |
| CBZ | 8.94 | 4.71 | 4.23 |
| TA | 2.36 | 2.36 | 0.00 |

**Table S5.** The decay rates of NB, BA, CBZ and DMOB with $Cl^-$.

| $k_{NB}$ ($10^{-4}$ $min^{-1}$) | $k_{BA}$ ($10^{-2}$ $min^{-1}$) | $k_{DMOB}$ ($10^{-2}$ $min^{-1}$) | $k_{CBZ}$ ( $min^{-1}$) |
|---|---|---|---|
| 1.62 | 6.92 | 8.90 | 1.26 |

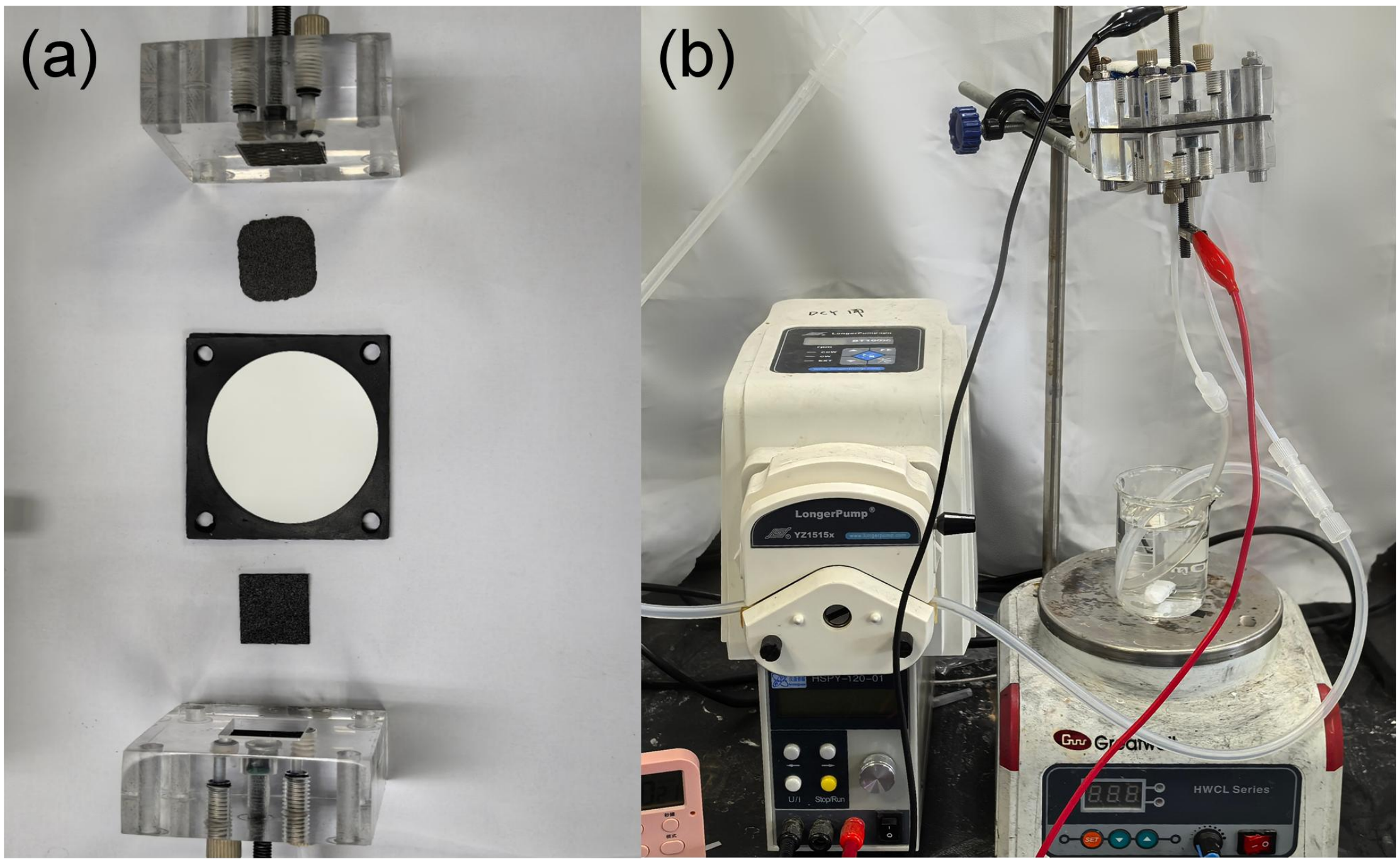


**Fig. S1 Structure of the apparatus. a-b,** (a) The internal structure of a continuous flow electrochemical reactor and (b) integral unit for ECH. The electrode measures 2 × 2 cm and the reactor measures 6 × 6 × 4 cm.

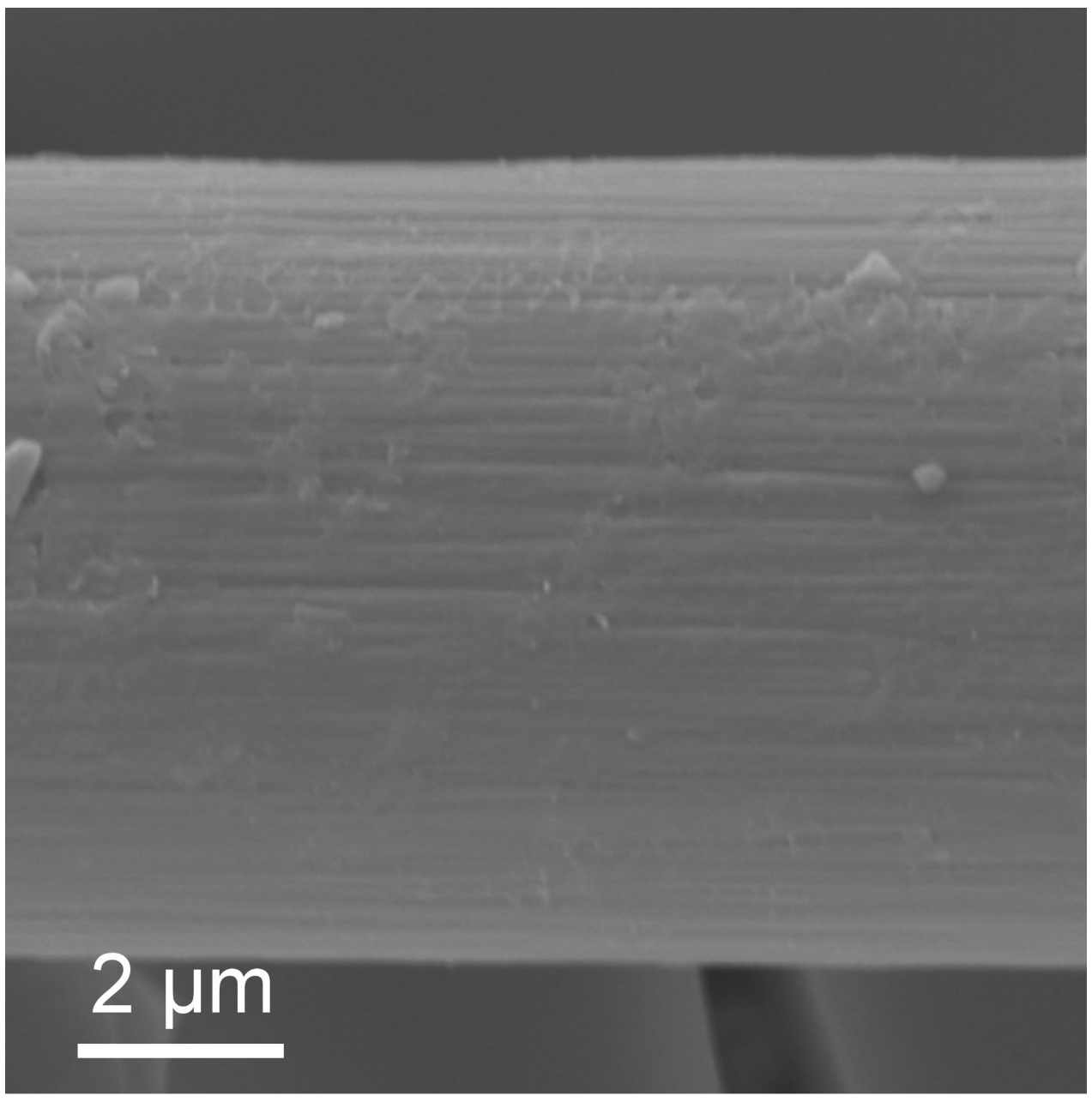


**Fig. S2 The SEM image.** The SEM image of the pure carbon paper.

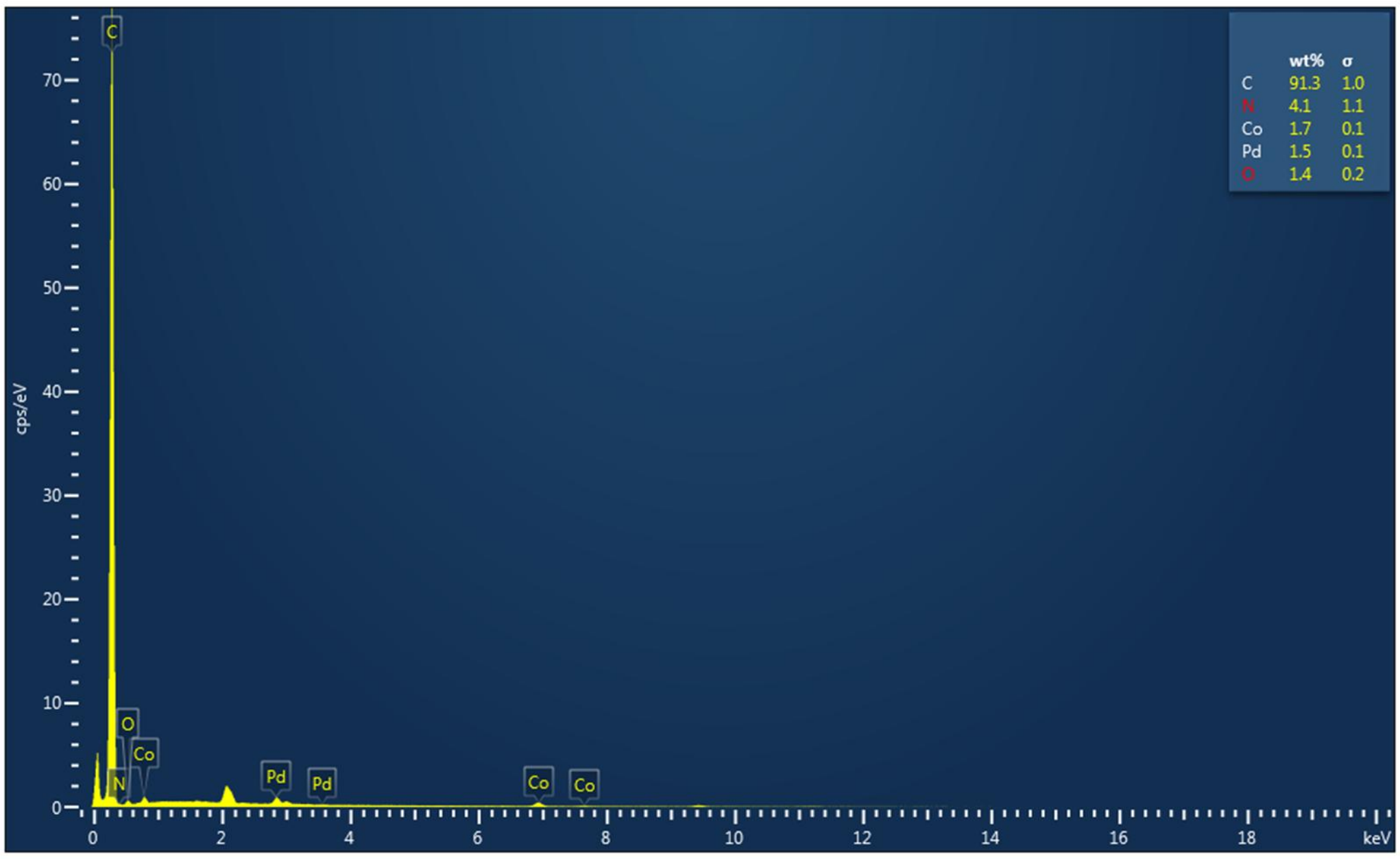


**Fig. S3 The corresponding EDS spectrum.** The corresponding EDS spectrum of PdCo/CP cathode.

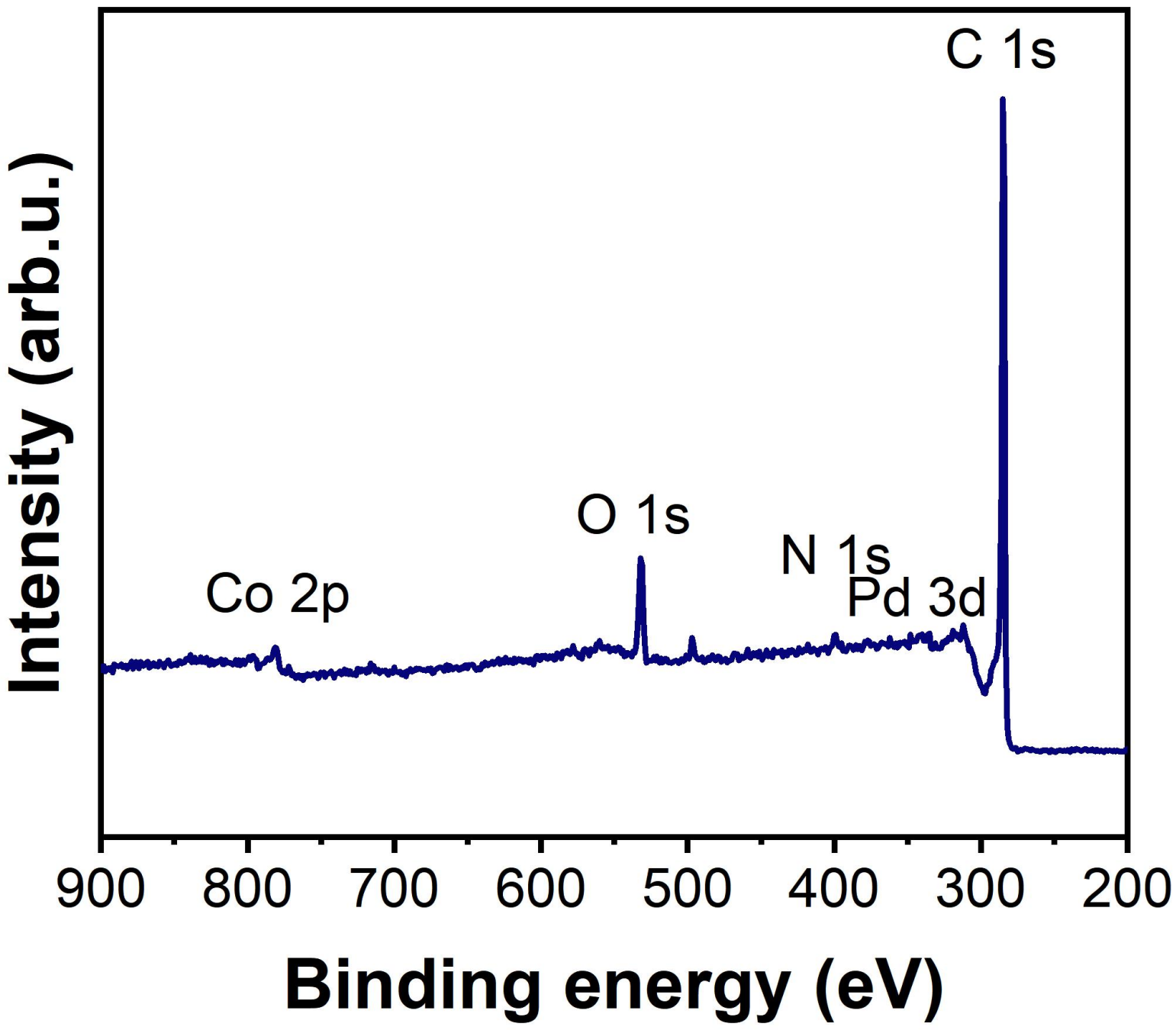


**Fig. S4 The wide scan XPS spectra.** The wide scan XPS spectra of PdCo/CP.

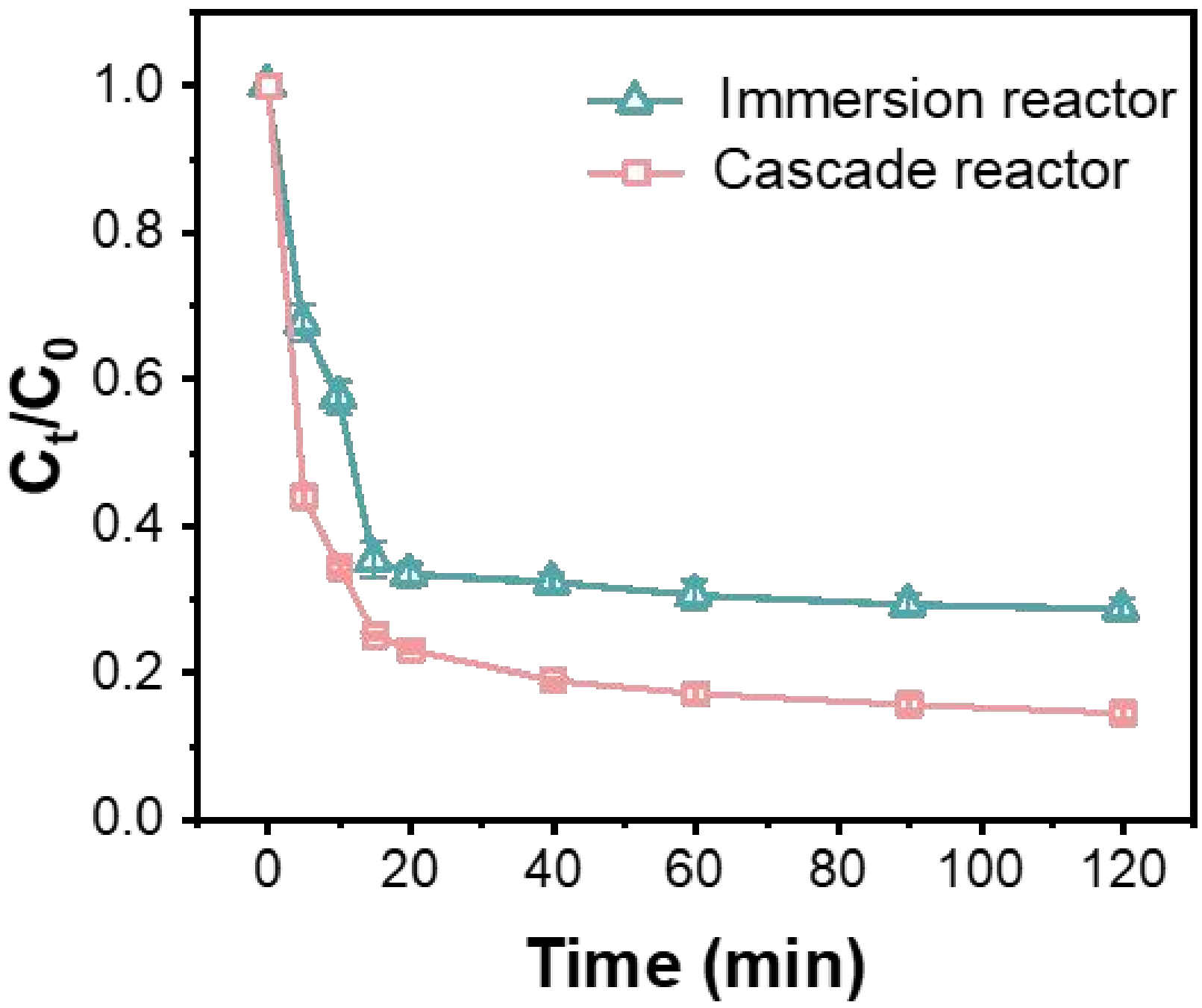


**Fig. S5 Degradation efficiency of different reactors.** 2,4,6-TCP dechlorination performance with immersion reactor and cascade reactor. Data are presented as mean ± s.d. from n = 3 independent experiments (reactions were independently prepared and run on separate days). n represents the number of independent experiments, not technical replicates. Source data are provided as a Source Data file.

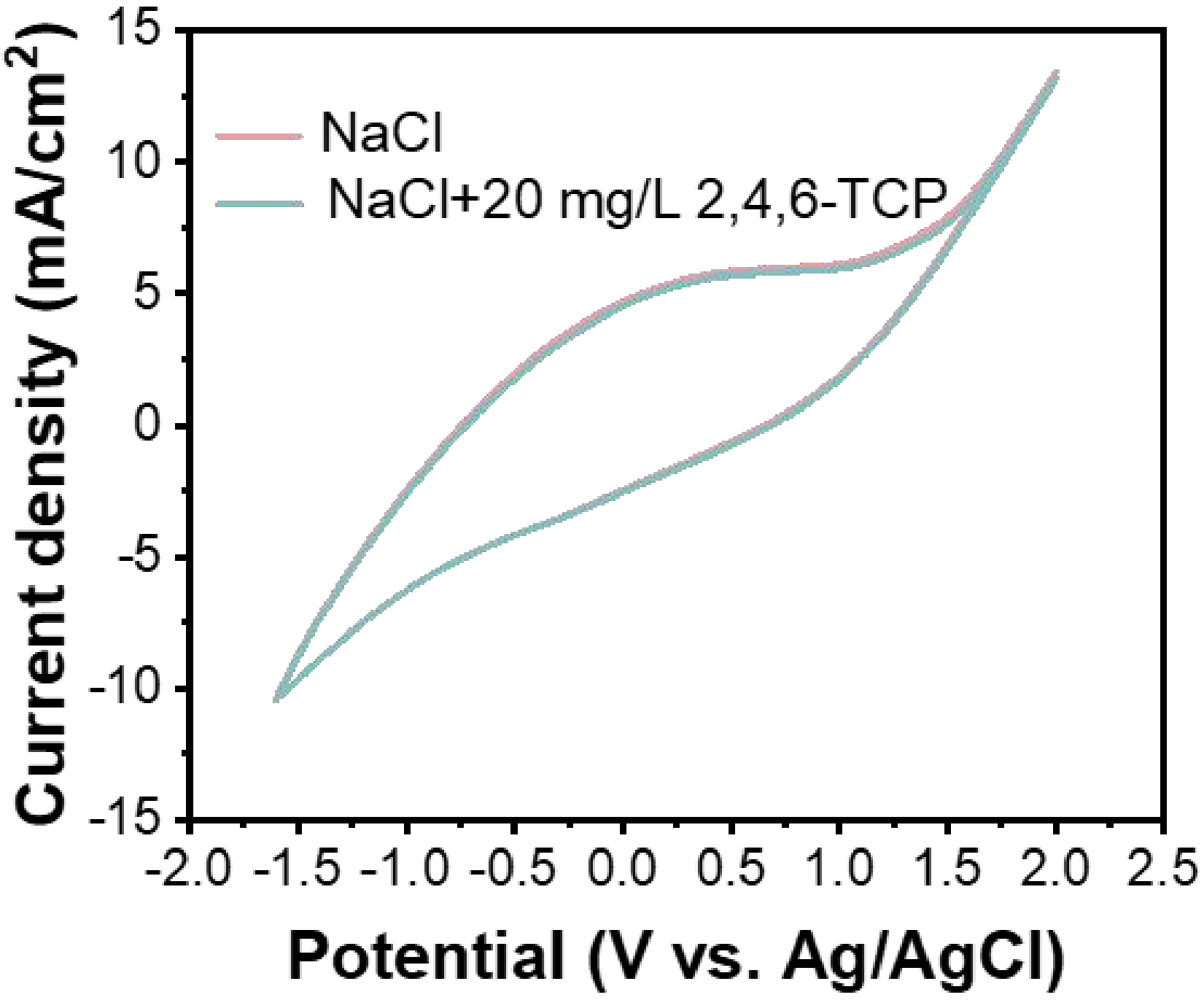


**Fig. S6 Cyclic voltammogram (CV).** The CV curves of 2,4,6-TCP in the NaCl solutions.

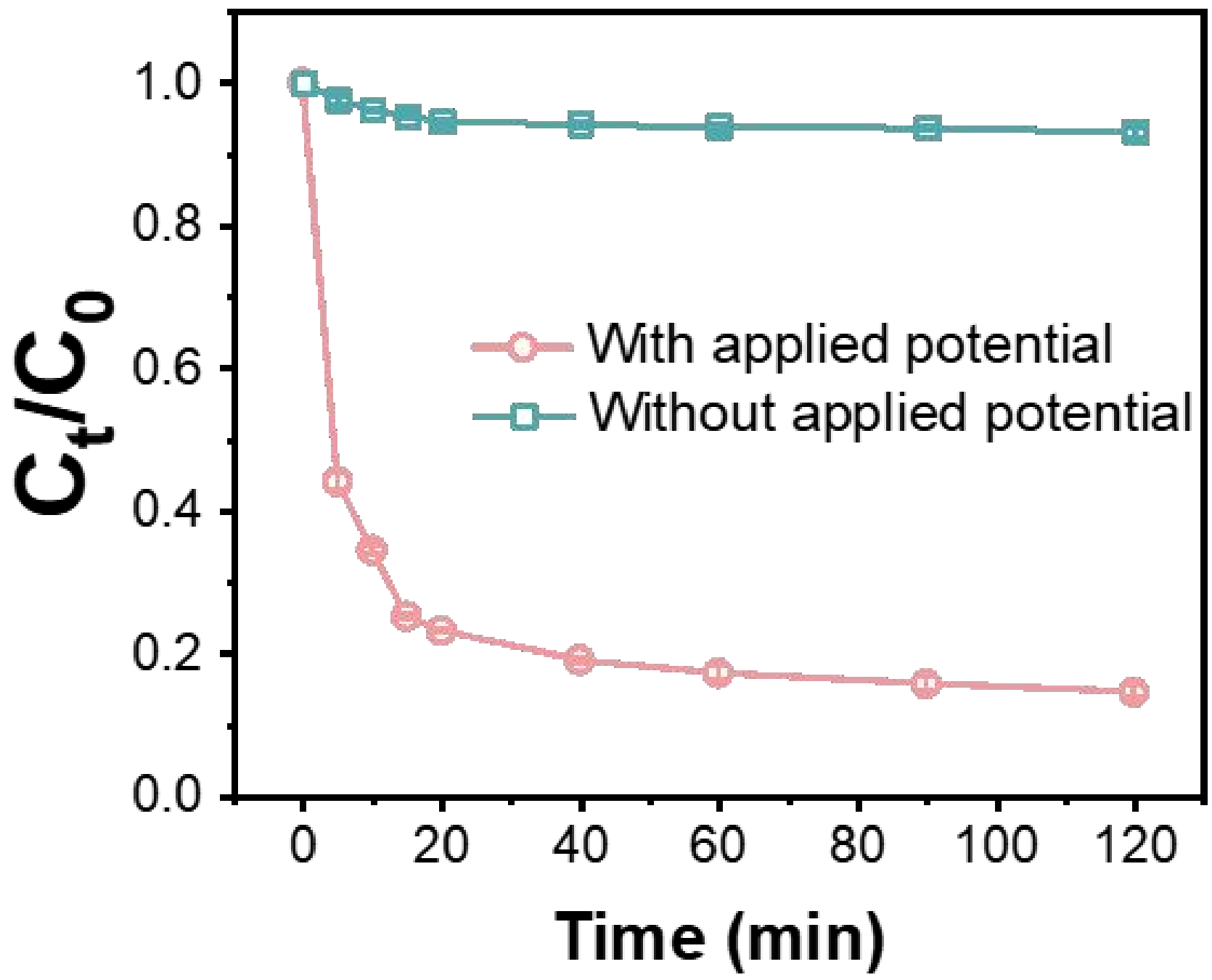


**Fig. S7 The effect of adsorption on degradation.** The degradation efficiency of 2,4,6-TCP with and without applied potential. Data are presented as mean ± s.d. from n = 3 independent experiments (reactions were independently prepared and run on separate days). n represents the number of independent experiments, not technical replicates. Source data are provided as a Source Data file.

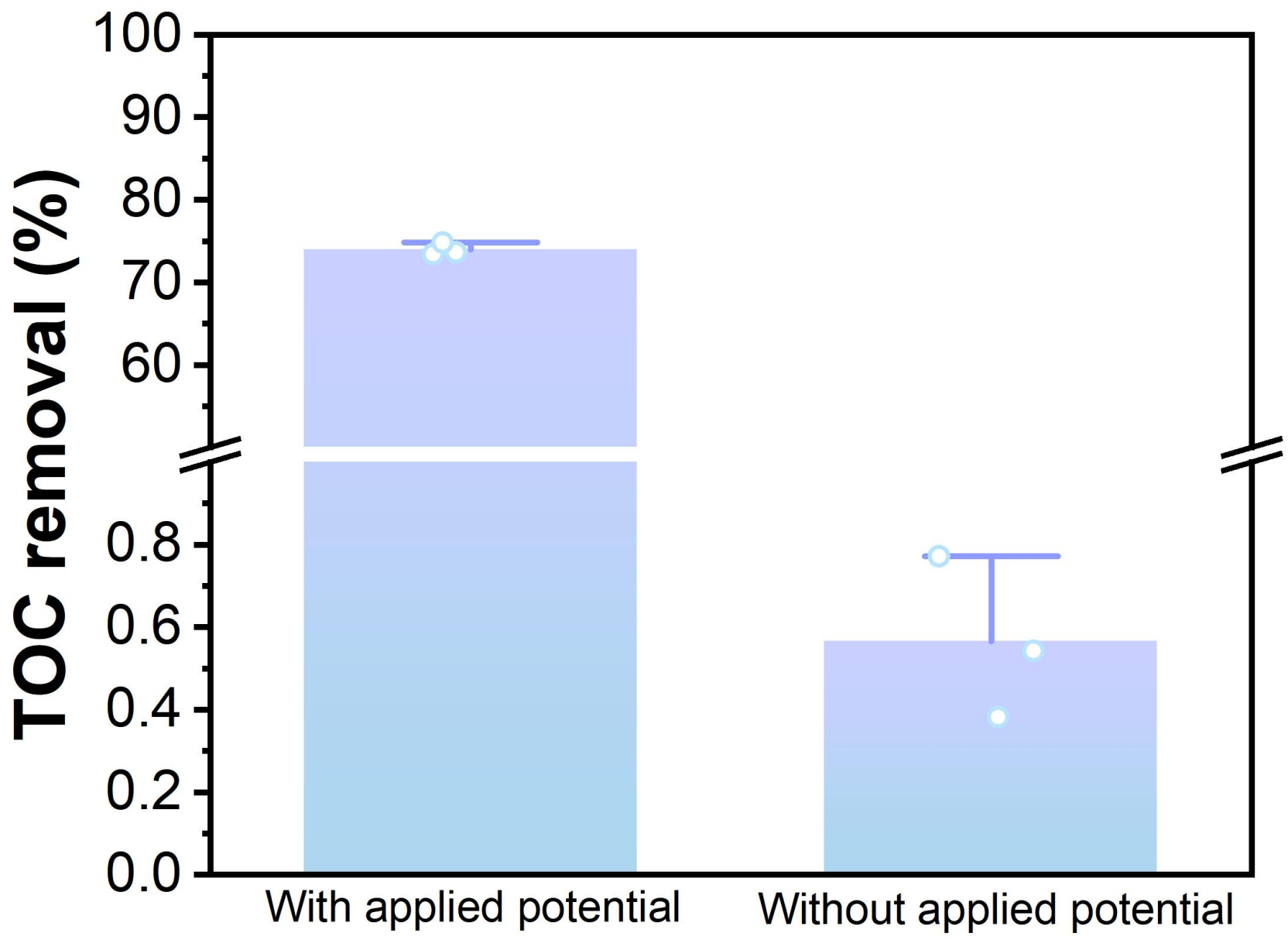


**Fig. S8 The effect of adsorption on TOC removal.** The TOC removal of 2,4,6-TCP with and without applied potential. Data are presented as mean ± s.d. from n = 3 independent experiments (reactions were independently prepared and run on separate days). n represents the number of independent experiments, not technical replicates. Source data are provided as a Source Data file.

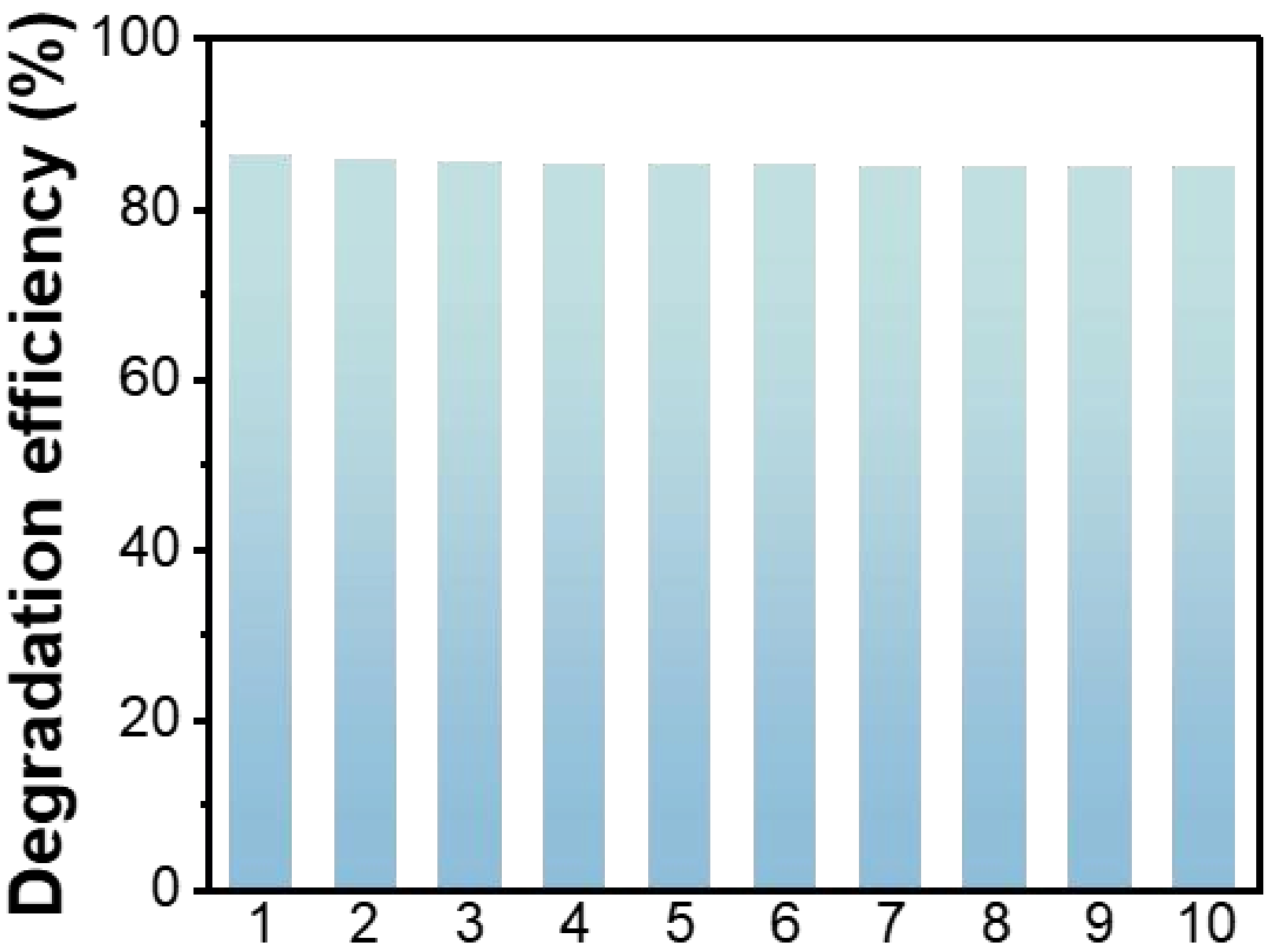


**Fig. S9 The stability test.** Degradation performance with 10 reuses of the electrode with the cascade reactor.

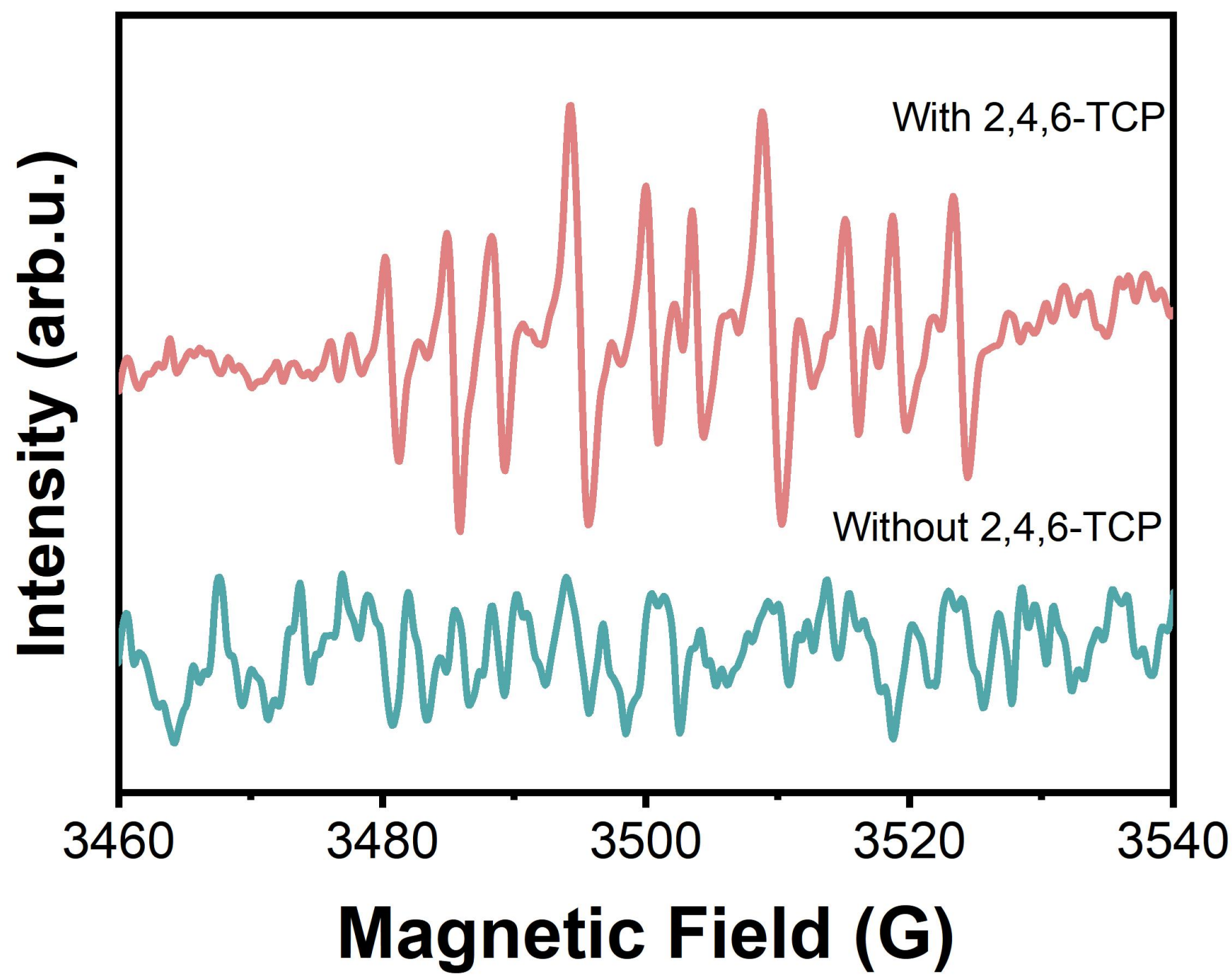


**Fig. S10 The RCS signal.** EPR spectra of RCS trapped by PBN with and without 2,4,6-TCP after reacting for 10 min.

.

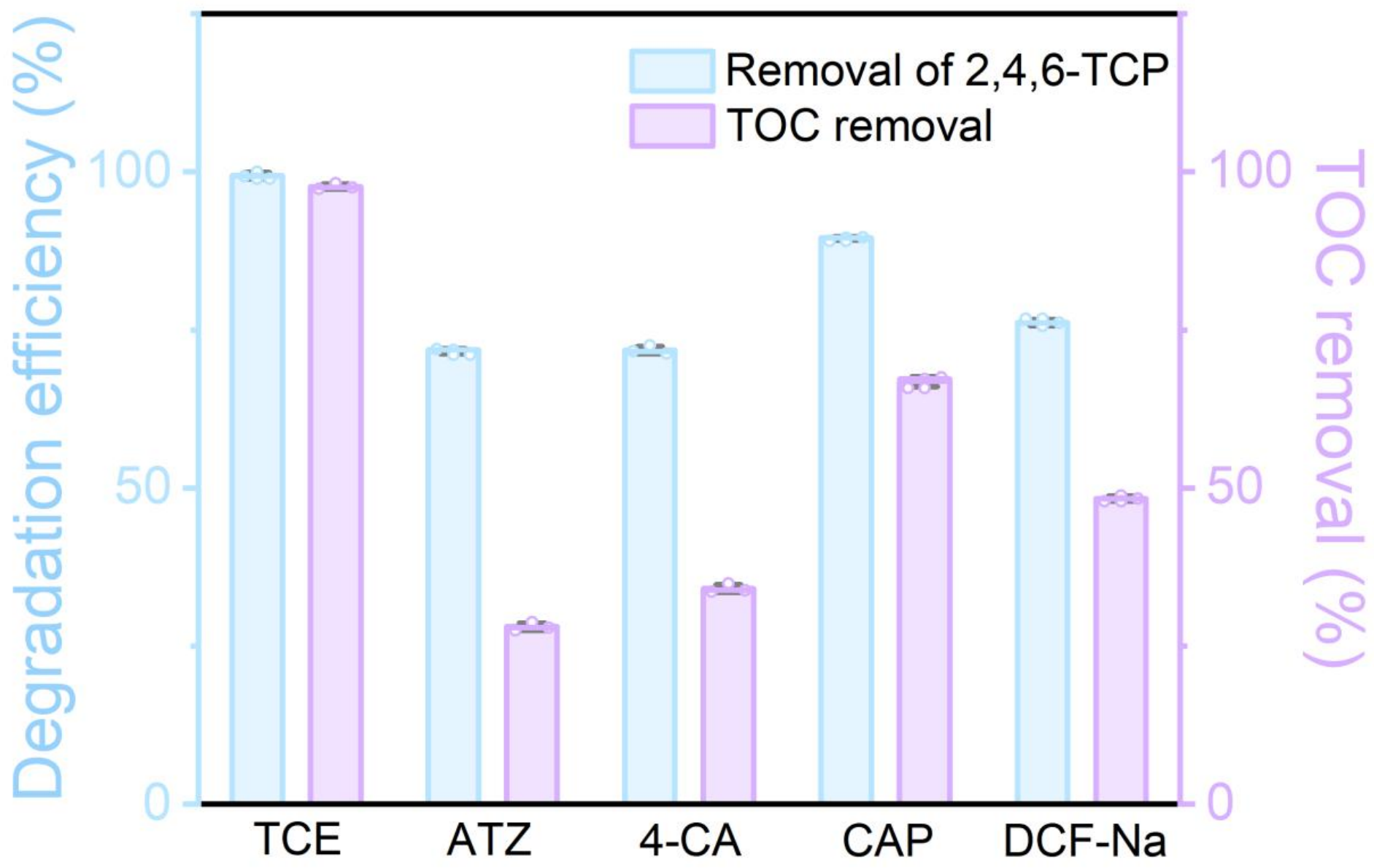


**Fig. S11 The effectiveness of cascade reactors on other pollutants.** The degradation efficiency and the TOC removal of non-phenolic COPs and aliphatic chlorinated compound. Data are presented as mean ± s.d. from n = 3 independent experiments (reactions were independently prepared and run on separate days). n represents the number of independent experiments, not technical replicates. Source data are provided as a Source Data file.

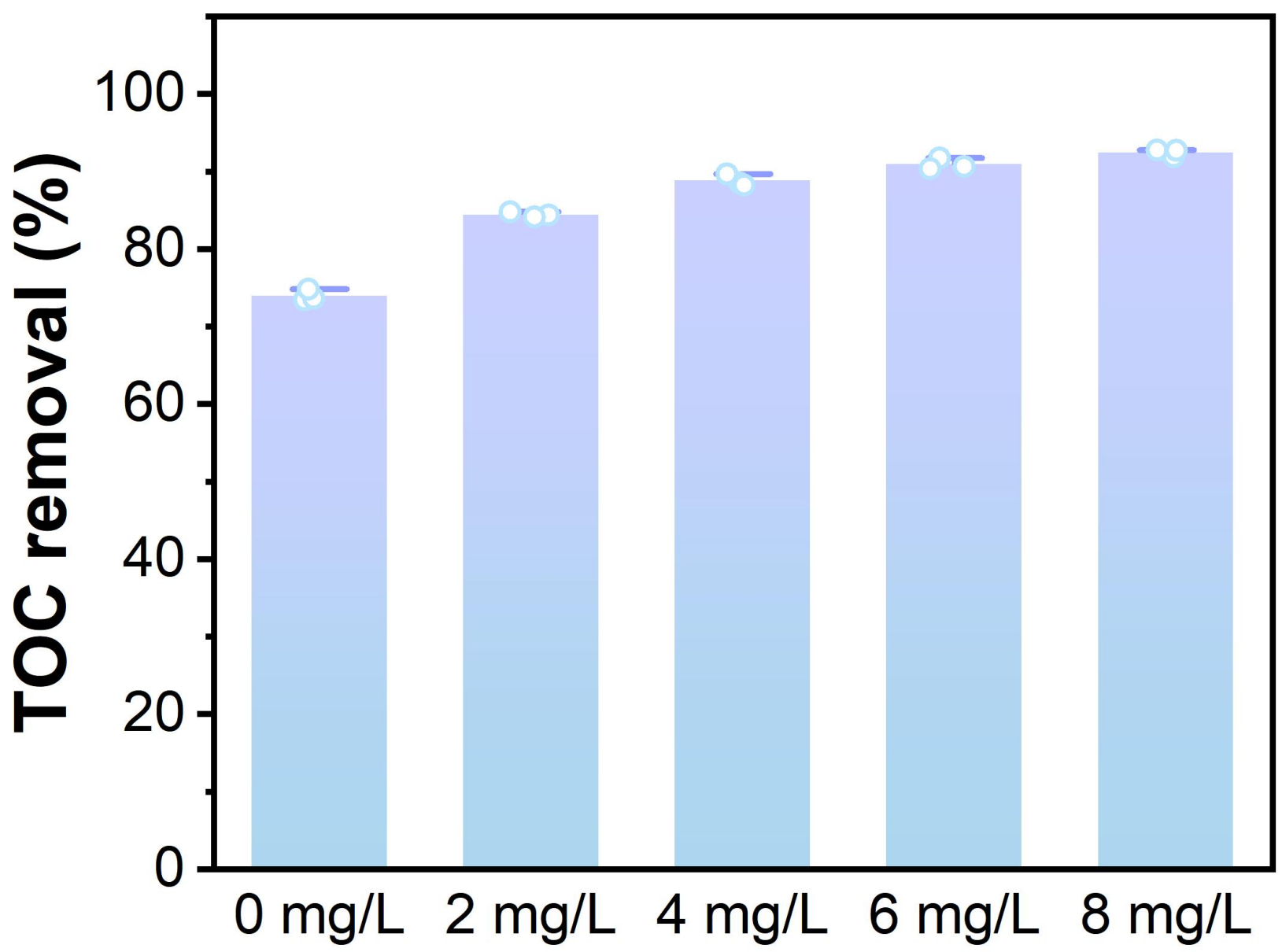


**Fig. S12 The effectiveness of $Cl^-$ concentrations.** The TOC removal of different additional $Cl^-$ concentrations. Data are presented as mean ± s.d. from n = 3 independent experiments (reactions were independently prepared and run on separate days). n represents the number of independent experiments, not technical replicates. Source data are provided as a Source Data file.

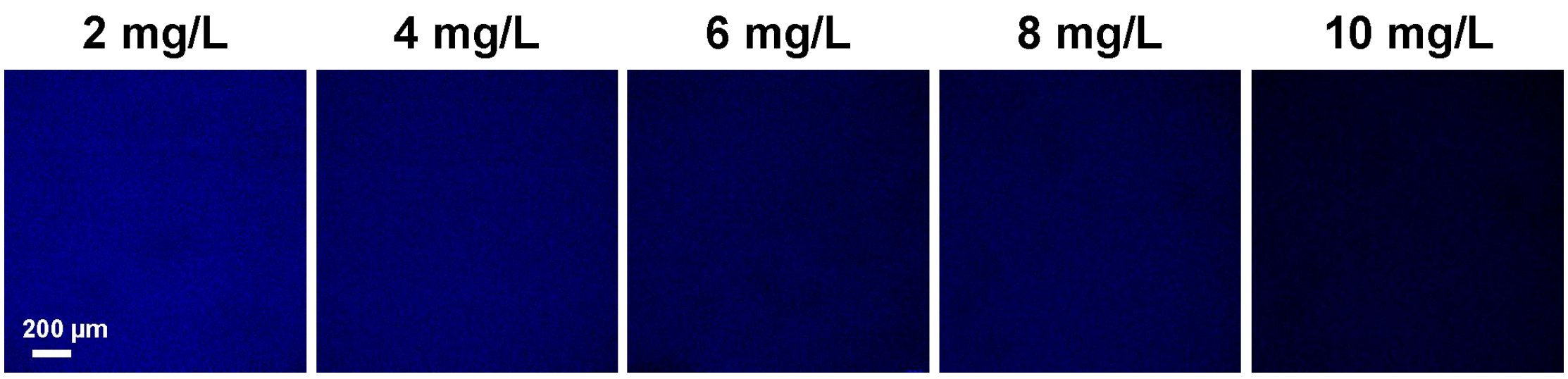


**Fig. S13 Fluorescence images of LSCM.** Fluorescence images of LSCM at different $Cl^-$ concentrations for standard curve.

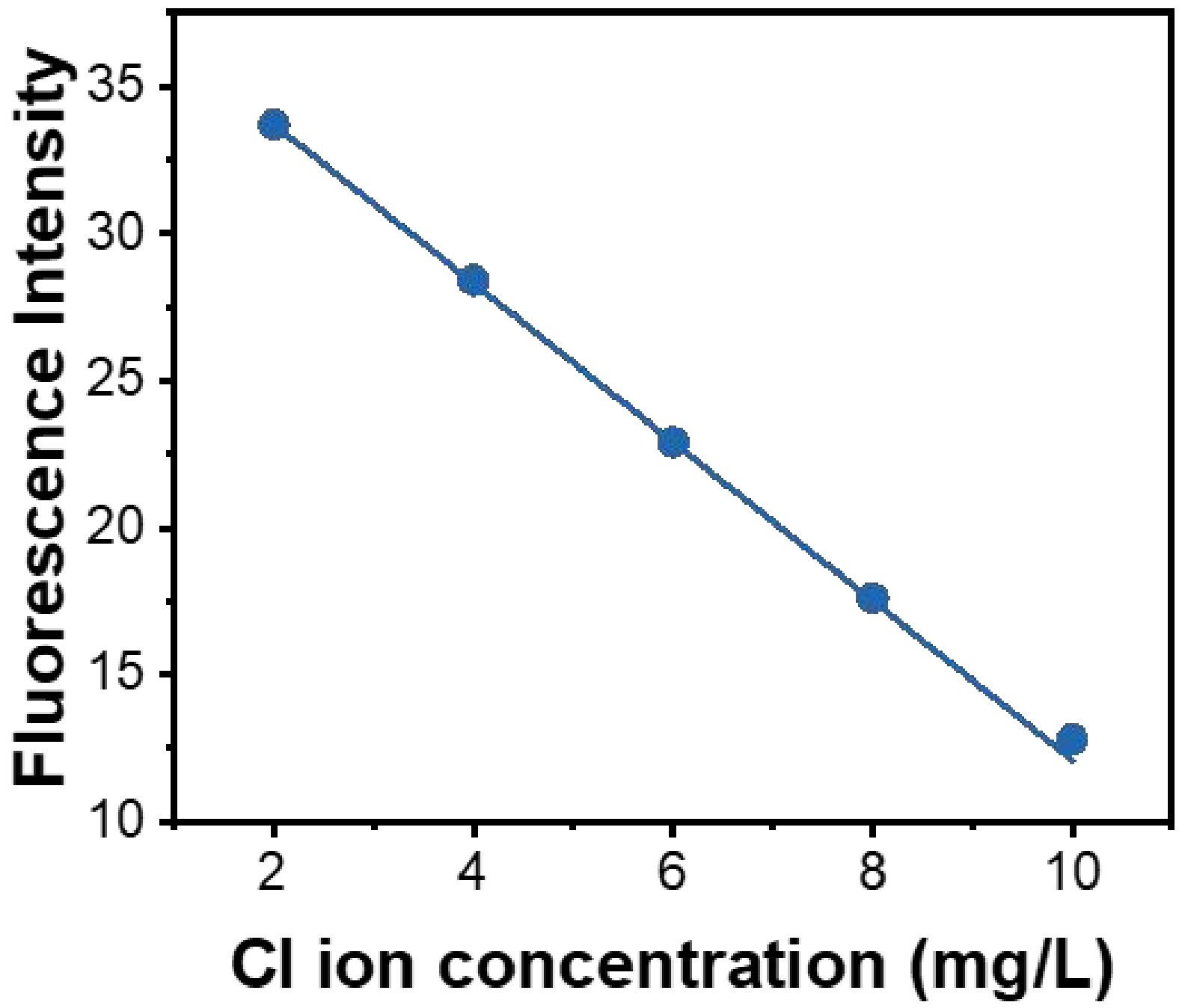


**Fig. S14 The standard curve.** The standard curve of chloride ion fluorescence intensity of MQAE. The solid line represents a linear fit to the experimental data ($y = -2.63x + 38.86$, $R^2 = 0.999$).

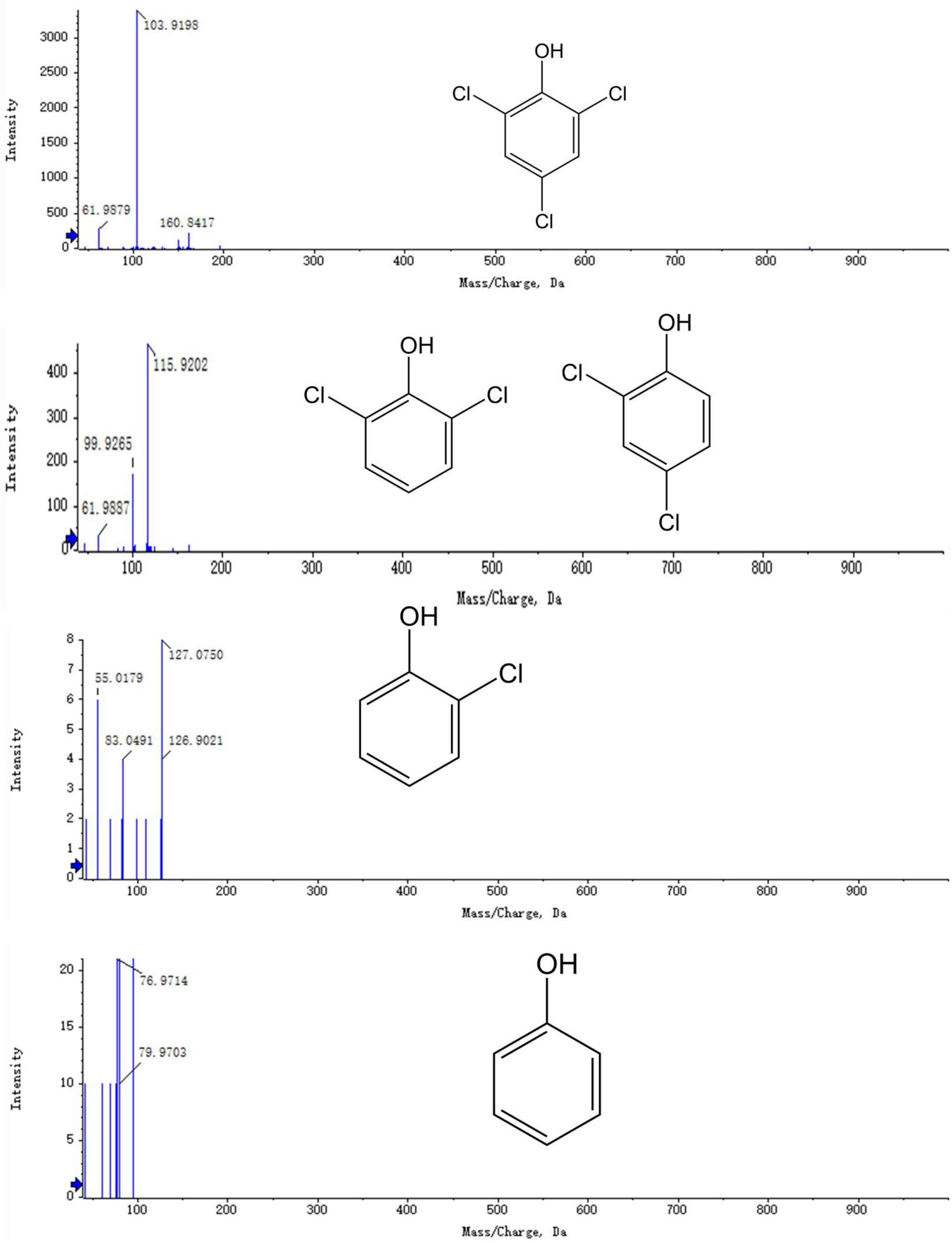


**Fig. S15 The reduction intermediates.** Mass spectra of 2,4,6-TCP degradation in reduction alone process at 5 min (electrocatalysis process in $N_2$-saturated solution, pH 4, 1.5V).

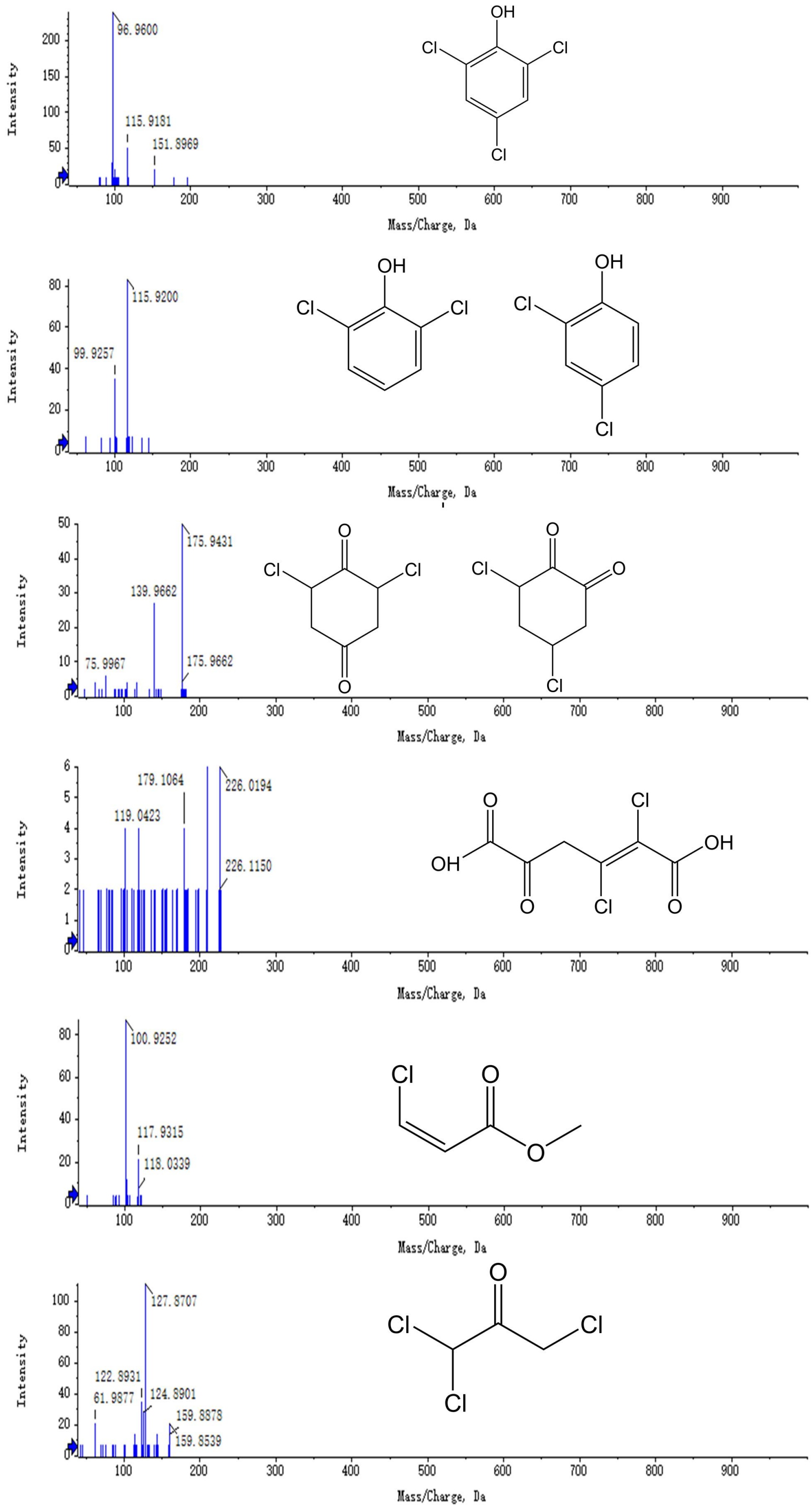


**Fig. S16 The oxidation intermediates.** Mass spectra of 2,4,6-TCP degradation in oxidation alone process at 5 min (electrocatalysis process in $N_2$-saturated solution, pH 4, 1.5V).

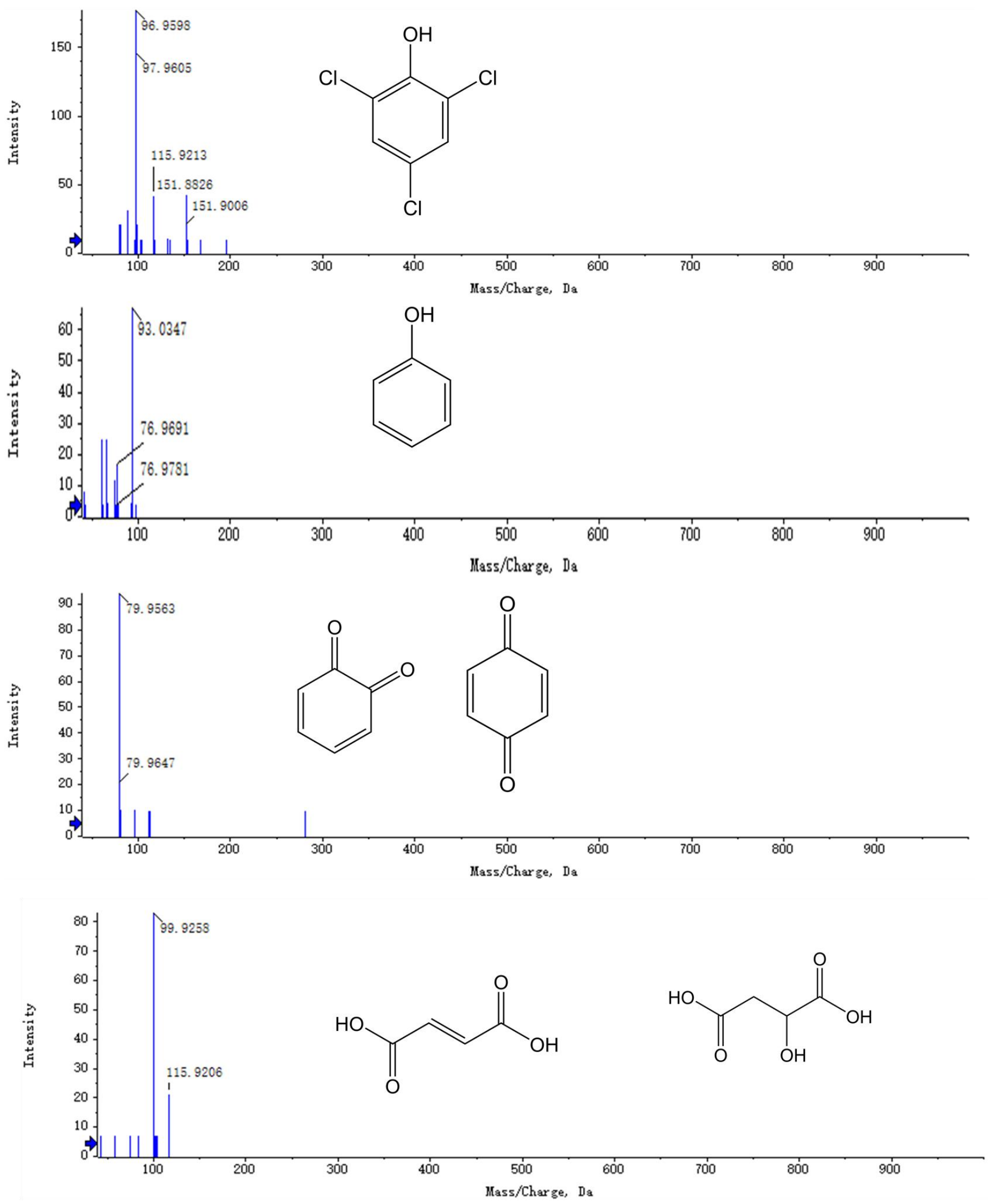


**Fig. S17 The redox intermediates.** Mass spectra of 2,4,6-TCP degradation in redox synergy process at 5 min (electrocatalysis process in $N_2$-saturated solution, pH 4, 1.5V).

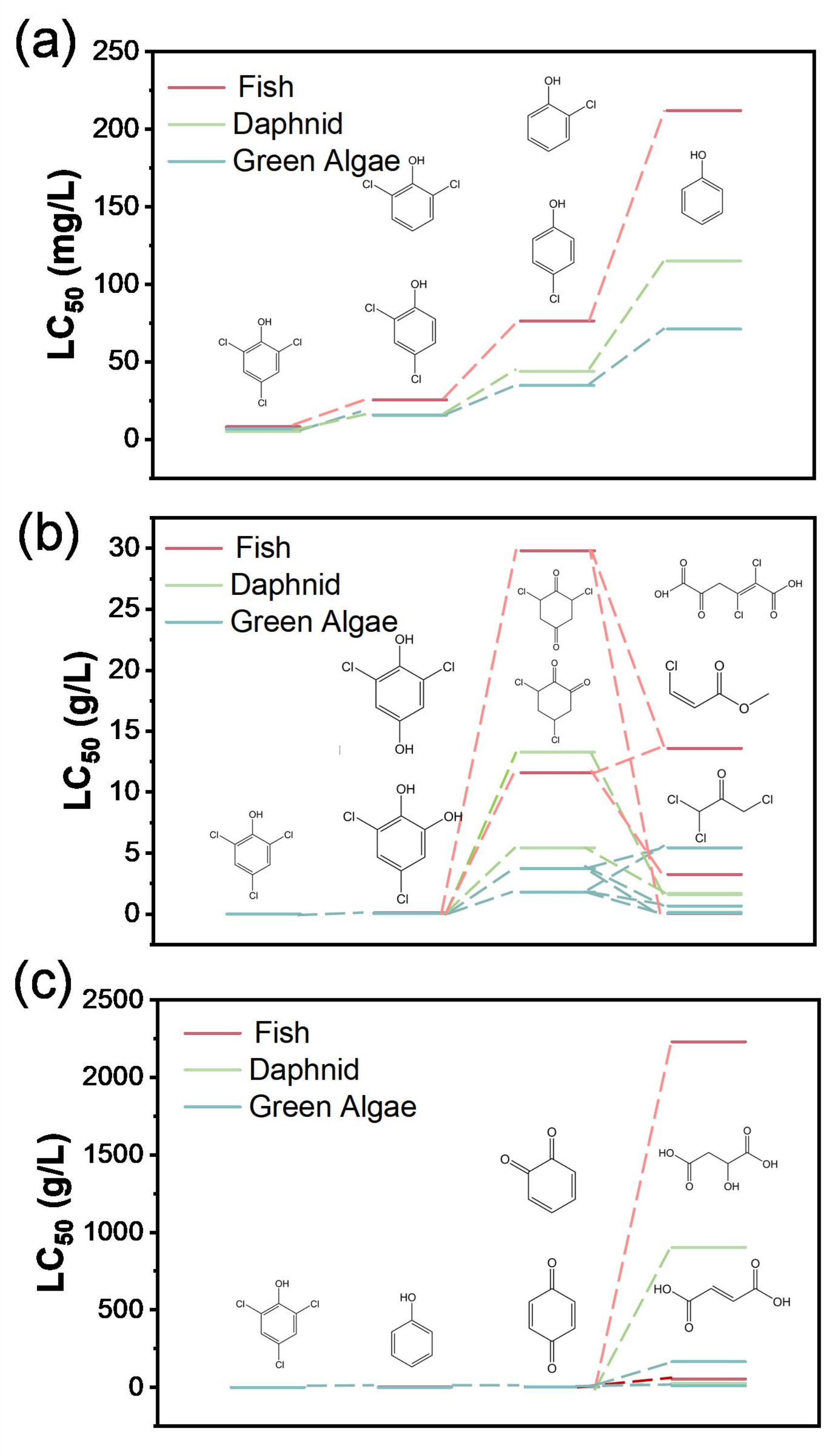


**Fig. S18 Toxicological analysis of different systems. a-c,** The toxicity of (a) reduction only, (b) oxidation only, and (c) redox tandem degradation products to fish, daphnid, and green algae (PdCo/CP as the cathode, $RuO_2$/Ti as the anode).
