# Supplementary material for: In-situ chlorine radical generation from captured Cl- for redox synergy in electrocatalytic hydrodehalogenation
Source: Nat Commun. 2026 Sep 4;17:10543. doi: 10.1038/s41467-026-77253-1 (PMC13642356; doi:10.1038/s41467-026-77253-1)
Supplement: Supplementary file 2 [file 41467_2026_77253_MOESM2_ESM.pdf]

nature portfolio

Peer Review File

In-Situ Chlorine Radical Generation from Captured Cl^- for Redox Synergy in Electrocatalytic Hydrodehalogenation

Corresponding Author: Professor Jiansheng Li

This file contains all reviewer reports in order by version, followed by all author rebuttals in order by version.

Version 0:

Reviewer comments:

Reviewer #1

(Remarks to the Author)
The manuscript presents a cascade chlorine cycling electrochemical reactor integrating a PdCo/CP cathode and a RuO_2/Ti anode for the efficient degradation of 2,4,6-trichlorophenol (2,4,6-TCP). The electrochemical assembly couples electrocatalytic hydrodechlorination (ECH) at the cathode with chlorine evolution reaction (CER) at the anode, enabling the in situ reutilization of chloride ions released during dechlorination. This redox tandem configuration demonstrates superior degradation efficiency and enhanced TOC removal compared to conventional reduction-only or oxidation-only systems, while simultaneously mitigating the accumulation of toxic chlorinated intermediates. From a practical standpoint, the integration of chloride recycling into a cascade electrochemical architecture represents a significant advance, placing this work on par with high-impact studies in the field. Beyond its applied relevance, the manuscript provides substantial mechanistic insight supported by comprehensive electrochemical characterization, EPR analysis, radical quenching experiments, LSCM visualization, UPLC-MS identification of intermediates, and complementary AIMD simulations. The study convincingly demonstrates a strategy in which released chloride ions are transformed into secondary oxidants, thereby promoting deeper mineralization of chlorinated organic pollutants. Overall, I consider this manuscript to be of significant interest to the readership of Nature Communications. However, I recommend publication after major revision to address the points outlined below.
1. What is the quantitative contribution of sequential dechlorination versus RCS-mediated oxidation to the overall enhancement observed in the redox tandem system?
2. Can the authors provide carbon balance or intermediate quantification data to directly correlate TOC removal with specific mineralization pathways? This is an important point to include briefly in the manuscript clearly.
3. related to question 2, Why does the oxidation-only process lead to the accumulation of more toxic chlorinated intermediates, whereas the redox tandem system shows continuous toxicity reduction?
4. Also relevant to question 2 and as mechanistic consideration. What differences in degradation pathways account for incomplete mineralization in reduction-only and oxidation-only systems compared to the tandem process? This is important point to delineate in the manuscript as the author claim tandem setup provides superior advantage over the simple reduction or oxidation approach.
5. How does the relative contribution of Cl· radicals compare with ·OH radicals in driving pollutant degradation in the tandem system? This is an important point to consider as OH radical is an integral part of advanced oxidation process (AOP) and contribution of Cl vs. OH radical needs to be clearly established.
6. Although quenching experiments suggest that Cl· is the dominant reactive species, can the authors quantify the relative contributions of Cl· versus ·OH to overall degradation using kinetic modeling or competitive scavenging analysis?
7. Can the authors quantitatively demonstrate the efficiency of Cl^- reutilization (e.g., percentage of released Cl^- converted to RCS versus remaining in solution), and how this correlates with enhanced mineralization? I suggest adding this control experiment to the manuscript to further elucidate this point.
8. What is the relationship between the increase in Cl^- concentration (reflected by conductivity changes) and the enhancement of redox synergy?
9. How do the formation and concentration of free chlorine differ among oxidation-only, reduction-only, and tandem systems, and how does this influence overall mineralization efficiency?
10. Cl3- is a known chloride oxidation pathway in chloride-containing electrochemical systems via the equilibrium Cl2+Cl−⇌Cl3−. What thermodynamic or interfacial arguments support the preferential formation of Cl_2 (or radical species such as Cl·) over Cl_3^- in this cascade reactor, and why would Cl_3^- formation be less favorable under the reported operating conditions?

11. Have the authors conducted control experiments under open-circuit conditions or without applied potential to exclude possible adsorption, non-electrochemical degradation, or spontaneous chemical reactions contributing to 2,4,6-TCP removal? If so, please provide the corresponding degradation and TOC data to confirm that the observed performance arises solely from the electrochemical processes.

Reviewer #2

(Remarks to the Author)
The manuscript developed a cascade electrochemical reactor for removing chlorinated organic pollutants (COPs) from water bodies. It proposed an innovative research direction that converts removed chloride ions into reusable active chlorine species. The topic is of significant importance and the manuscript was well prepared with plenty of data and insightful discussions. This work is publishable after the following comments were addressed well.
1. While the manuscript proposes a strategy to prevent catalyst poisoning by reusing chloride ions, sufficient comparison and differentiation from existing literature should be provided.
2. The work investigates the superiority of the redox tandem by simulating reduction-only and oxidation-only conditions using pure carbon paper electrodes for the cathode and anode, respectively. The purpose of the experiment using a proton exchange membrane to separate the anode and cathode chambers should be further explained in the manuscript.
3. Has the mechanism by which chloride ions are converted into active chlorine species (such as HOCl or Cl·) been fully elucidated? Any supporting data for this mechanism?
4. Since the PBN was used to capture chlorine radicals in the experiment, the specific conditions should be provided. It is recommended to supplement with detailed experimental parameters, such as PBN concentration and reaction duration.
5. The AIMD section mentions the helmholtz layer without discussion on its impact. Further explanation is recommended.
6. Why is pH 4 set as the reaction condition? Any basis or supporting evidence?
7. Some sentences contain spelling and grammatical typos that may compromise the paper's professionalism and readability. It is suggested that the author carefully review the entire manuscript and eliminate unnecessary spaces and formatting errors.

Reviewer #3

(Remarks to the Author)
Overall evaluation
This manuscript proposes a cascade electrochemical system that couples cathodic hydrodechlorination with anodic chlorine radical generation, enabling in situ reutilization of Cl^- for deep mineralization of chlorinated pollutants.
The manuscript claims a “new strategy”, but redox coupling (ECH + oxidation) is already widely reported (see refs 5, 9, etc.). And, chloride-mediated oxidation (RCS/CER) is also well established. So the true novelty (chloride self-recycling) is incremental and not clearly distinguished from, electro-Fenton + halide systems, paired electrolysis and self-generated oxidant systems.
There is insufficient quantitative evidence for Cl^- → RCS reutilization. This is the central claim, but evidence is indirect, e.g., EPR confirms Cl· presence, but not its origin (from released Cl^- vs electrolyte/background); No isotope labeling or mass balance; Free chlorine measured is very low (~0.2 mg/L, page 19). Chlorine mass balance, FE toward CER vs OER, as well as quantitative RCS yield are missing.
Based on the current shape of the manuscript, I would recommend a major revision for the subsequent consideration of publication.
The below are some more specific questions:
Q1. How do you prove that RCS originates from released Cl^- rather than background electrolyte or electrode processes?
Q2. What is the Faradaic efficiency of CER vs OER at 1.5 V?
Q3. Can the author quantify relative contributions of Cl·, $Cl_2{\cdot}^-$, ClO·, and ·OH?
Q4. What is the energy consumption per mg TOC removed?
Q5. How stable is PdCo/CP over multiple cycles? Any metal leaching?
Q6. How does performance change with initial chloride concentration?
Q7. Does the system work for non-phenolic COPs or aliphatic chlorinated compounds?
Q8. Why is free chlorine so low (~0.2 mg/L) if CER is significant?
Q9. Could the observed enhancement simply be due to improved mass transfer, not Cl^- recycling?

Version 1:

Reviewer comments:

Reviewer #1

(Remarks to the Author)
The revised manuscript is of very high quality, and I am satisfied with the authors' responses to the questions raised, as well as the changes implemented throughout the manuscript. I recommend publication without further revisions, as the study is complete and timely. The results are well presented, logically connected, and supported by adequate explanations throughout the manuscript. Finally, sufficient detail regarding the experimental procedures is provided.

Reviewer #2

(Remarks to the Author)
My comments have been properly addressed.

Reviewer #3

(Remarks to the Author)
I appreciate the efforts by the authors. The revised manuscript is now in a good shape for publishing in Nat. Commun..

Open Access This Peer Review File is licensed under a Creative Commons Attribution 4.0 International License, which permits use, sharing, adaptation, distribution and reproduction in any medium or format, as long as you give appropriate credit to the original author(s) and the source, provide a link to the Creative Commons license, and indicate if changes were made.
In cases where reviewers are anonymous, credit should be given to 'Anonymous Referee' and the source.
The images or other third party material in this Peer Review File are included in the article's Creative Commons license, unless indicated otherwise in a credit line to the material. If material is not included in the article's Creative Commons license and your intended use is not permitted by statutory regulation or exceeds the permitted use, you will need to obtain permission directly from the copyright holder.
To view a copy of this license, visit https://creativecommons.org/licenses/by/4.0/

Point to Point Response

(NCOMMS-26-013704-T)

Reply to the comments from reviewers

We sincerely thank the Editor and Reviewers for their careful evaluation of our manuscript and for their constructive comments. We fully recognize that these issues are crucial for improving the rigor, clarity, and overall persuasiveness of the study. In response to these concerns, we have made systematic and substantial revisions to the manuscript and have carried out additional experiments and analyses. Specifically, we have strengthened the evidence for the reutilization of chlorine-derived reactive species and their relative contributions, clarified more clearly the novelty of this work in comparison with previous related studies, evaluated the cycling stability of the electrode, and extended the applicability of the system to additional non-phenolic and aliphatic chlorinated pollutants. Our detailed point-by-point responses to all comments are provided below.

Reviewer comments

Reviewer 1:

The manuscript presents a cascade chlorine cycling electrochemical reactor integrating a PdCo/CP cathode and a RuO_2/Ti anode for the efficient degradation of 2,4,6-trichlorophenol (2,4,6-TCP). The electrochemical assembly couples electrocatalytic hydrodechlorination (ECH) at the cathode with chlorine evolution reaction (CER) at the anode, enabling the in situ reutilization of chloride ions released during dechlorination. This redox tandem configuration demonstrates superior degradation efficiency and enhanced TOC removal compared to conventional reduction-only or oxidation-only systems, while simultaneously mitigating the accumulation of toxic chlorinated intermediates. From a practical standpoint, the integration of chloride recycling into a cascade electrochemical architecture represents a significant advance, placing this work on par with high-impact studies in the field. Beyond its applied relevance, the manuscript provides substantial mechanistic insight supported by comprehensive electrochemical characterization, EPR analysis, radical quenching experiments, LSCM visualization,

UPLC-MS identification of intermediates, and complementary AIMD simulations. The study convincingly demonstrates a strategy in which released chloride ions are transformed into secondary oxidants, thereby promoting deeper mineralization of chlorinated organic pollutants. Overall, I consider this manuscript to be of significant interest to the readership of Nature Communications. However, I recommend publication after major revision to address the points outlined below.

Response:

Thank you very much for your highly recognition of our work and for your valuable suggestions. We have carefully considered the comments and made corresponding revisions. The specific revision are presented below.

Comment 1-1: What is the quantitative contribution of sequential dechlorination versus RCS-mediated oxidation to the overall enhancement observed in the redox tandem system?

Response: Thank you for raising this important question. We agree that clarifying the relative contributions of stepwise dechlorination and RCS-mediated oxidation to the overall performance enhancement is highly important. Since these two processes are continuously coupled and mutually reinforced in the tandem system, it is difficult to strictly separate their contributions in an absolute sense. Therefore, a semi-quantitative attribution strategy was adopted.

Specifically, we first constructed a reduction-only system in which the anode was replaced by carbon paper, in order to evaluate the independent role of cathodic stepwise dechlorination. The results showed that this process mainly induced C–Cl bond cleavage and converted 2,4,6-TCP into lower-chlorinated or dechlorinated intermediates, while its contribution to TOC removal remained limited (Fig.2h). We then analyzed the composition of the electrolyte after the reduction-only reaction and, based on these results, prepared a electrolyte containing the corresponding concentrations of Cl^{-}, 2,4,6-TCP and the representative intermediate phenol. Oxidation-only experiments were subsequently performed in the presence and absence of Cl^{-} (Fig.3f). Under otherwise identical conditions, in the presence of Cl^{-}, the removal efficiencies of 2,4,6-TCP and phenol reached 82.8% and 98.3%, respectively, and the TOC removal efficiency reached 73.2%, which was close to that observed in the tandem redox system. In

contrast, in the absence of Cl^-, the TOC removal efficiency was only 12.8%. This corresponds to an additional 60.4 percentage points of TOC removal enhancement in the oxidation stage, strongly indicating that Cl^--derived RCS play a dominant role in deep oxidation and mineralization.

Based on these results, we conclude that the enhanced performance of the tandem system arises from the sequential synergy between these two steps: cathodic stepwise dechlorination is primarily responsible for precursor transformation and detoxification, whereas anodic RCS-mediated oxidation mainly drives the subsequent ring opening and deep mineralization. Thus, although the two processes cannot be completely separated in a strict quantitative sense, the above comparison provides a semi-quantitative attribution showing that RCS-mediated oxidation is the key contributor to the TOC removal enhancement in the tandem system. The corresponding supplementary results and discussion have been added to the revised manuscript and the Supplementary Information.

The Fig. 3 was revised into:

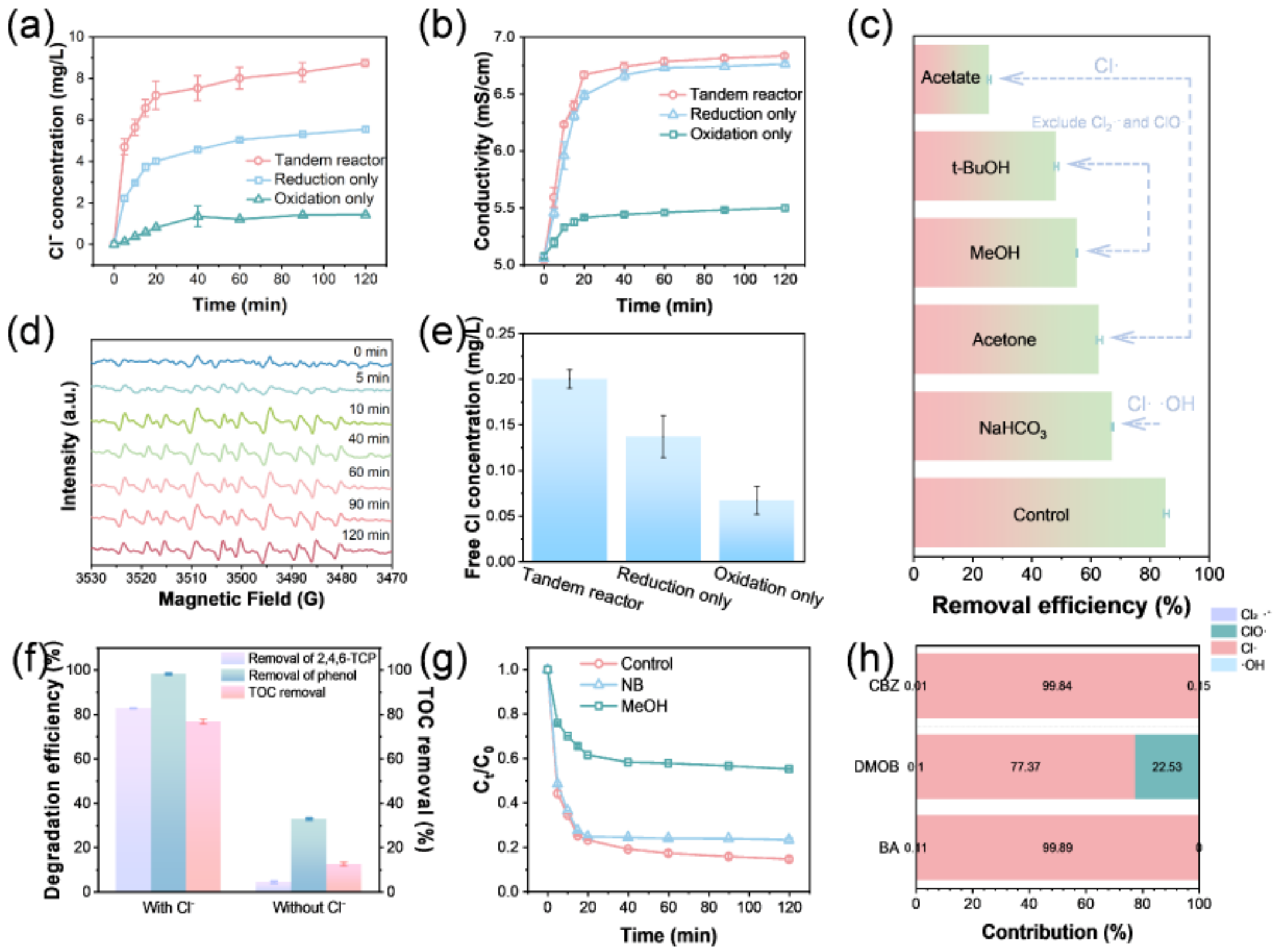

Fig.3 Evidence of the conversion of free chlorine ions to RCS. a,b, (a) Cl^- concentration and (b) Time-dependent variation in electrical conductivity in oxidation only, reduction only, and tandem reactor. **c,** 2,4,6-TCP quenching experiment in tandem reactor. **d,** EPR spectra of RCS trapped by PBN. **e,** Free Cl concentration in oxidation only, reduction only, and tandem reactor. **f,** The degradation efficiency and the TOC removal of simulated electrolyte after reduction-only reaction in the presence or absence of Cl^-. **g,** Quenching of ·OH: [MeOH] = [NB] = 5 mM. **h,** Relative radical contributions to the probe decay of BA, DMOB, and CBZ derived from probe-kinetic analysis.

In lines 432-453, the paragraph "To semi-quantitatively evaluate the contribution of the RCS-mediated oxidation process to the overall performance enhancement of the system, the composition of the electrolyte after the reduction-only reaction was first analyzed, which mainly contained Cl^-, 2,4,6-TCP, and its representative dechlorinated intermediate, phenol. Based on this composition, a simulated electrolyte was further prepared, and oxidation-only experiments were conducted in the presence and absence of Cl^- to investigate the role of RCS-mediated oxidation in the overall system performance. As shown in Fig.3f, in the presence of Cl^-, the removal efficiencies of 2,4,6-TCP and phenol reached 82.8% and 98.3%, respectively, and the TOC removal efficiency reached 76.72%, which was comparable to the mineralization performance of the redox tandem system. In contrast, in the absence of Cl^-, the TOC removal efficiency was only 12.46%. This result indicates that the presence of Cl^- provided an additional 60.4 percentage points of TOC removal enhancement during the subsequent oxidation stage, strongly suggesting that Cl^--derived RCS play a dominant role in deep oxidation and mineralization. Combined with the reduction-only results, it can be inferred that sequential dechlorination mainly contributes to C–Cl bond cleavage, precursor transformation, and detoxification, whereas RCS-mediated oxidation is primarily responsible for the subsequent ring opening and deep mineralization. Therefore, the performance enhancement of the tandem system arises from the sequential synergy between these two processes, with RCS-mediated oxidation being the key contributor to TOC removal improvement." was added.

Comment 1-2: Can the authors provide carbon balance or intermediate quantification data to directly correlate TOC removal with specific mineralization pathways? This is an important point to include briefly in the manuscript clearly.

Response: Thank you for raising this important point. We agree that directly correlating TOC removal with the proposed mineralization pathway is essential for clarifying the reaction process in the tandem system. In response to this comment, we further analyzed the temporal evolution of the major intermediates in the redox tandem system by LC-MS and correlated these results with the TOC removal profile, thereby strengthening the discussion linking TOC removal to the coupled reductive–oxidative process. Specifically, as shown in Fig.7d, at the early stage of the reaction, the concentrations of dechlorinated intermediates, including DCP, CP, and phenol, gradually increased as stepwise dechlorination proceeded. Subsequently, with the further progress of the RCS-mediated oxidation process, more oxidized products, such as benzoquinone and low-molecular-weight organic acids, were continuously formed. At the later stage, the concentrations of all these intermediates gradually decreased, while the TOC continuously declined, indicating that these products were further oxidized and eventually driven toward mineralization. Overall, these results demonstrate that, in the tandem system, the parent pollutant was first transformed into lower-chlorinated or dechlorinated intermediates, which were subsequently converted into more highly oxidized products, accompanied by a continuous decrease in total organic carbon. This behavior is consistent with the proposed deep mineralization pathway. Therefore, these results provide more direct evidence linking TOC removal with the proposed mineralization process. The corresponding discussion has now been added to the revised manuscript and the Supplementary Information.

The Fig.7 was revised into:

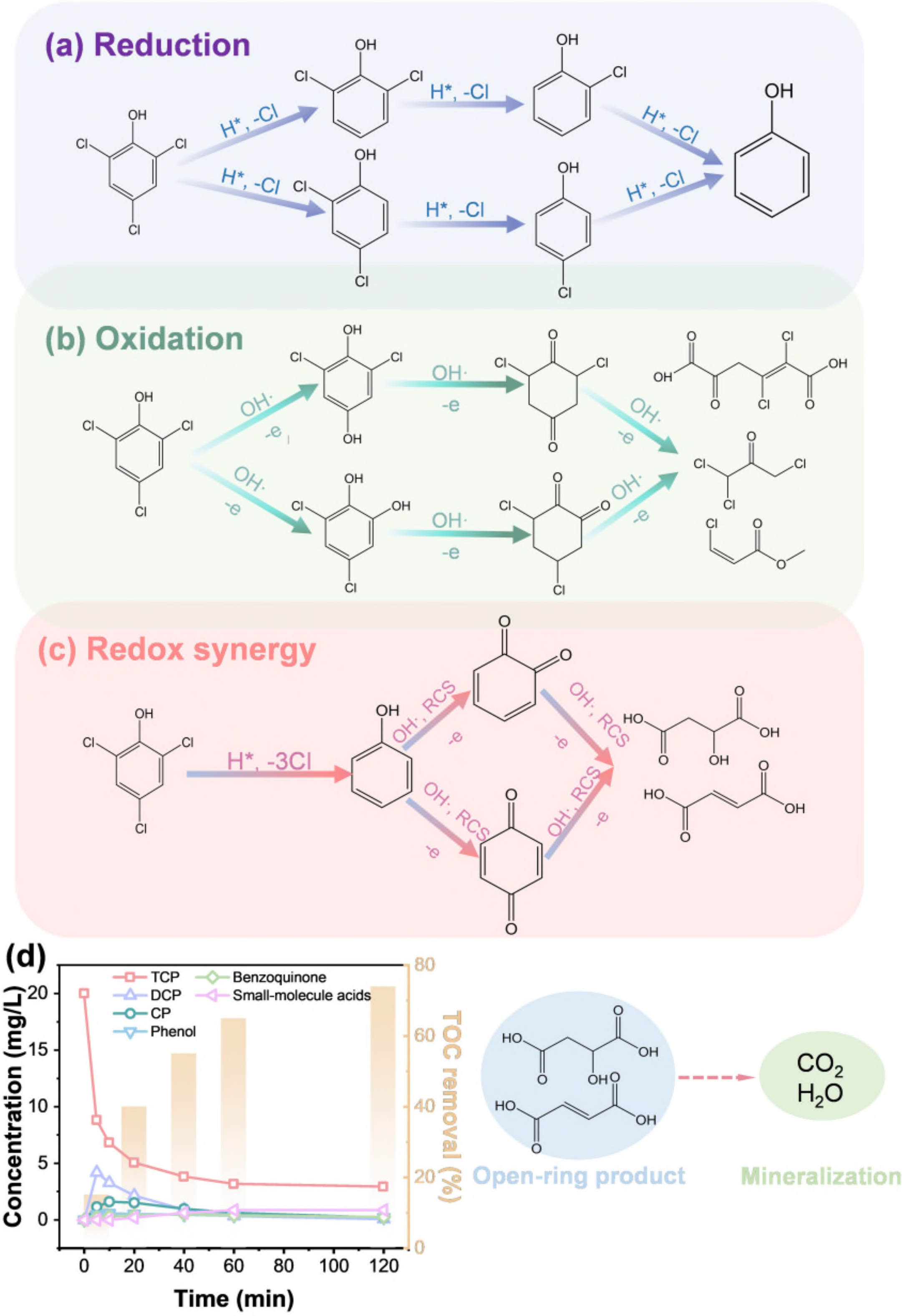

Fig.7 Degradation pathways of 2,4,6-TCP in different systems. a-c, The schematic diagram of reaction pathways of PdCo/CP cathode-RuO_2/Ti anode for 2,4,6-TCP degradation via (a) reduction only, (b) oxidation only, and (c) redox tandem. **d,** Concentration changes of intermediate products and TOC removal in the redox tandem system.

The Tables S2 and 3 was added into Supplementary Information:

Table S2. Table of Intermediate Product Concentrations.

Time (min)	TCP (mg/L)	DCP (mg/L)	CP (mg/L)	Phenol (mg/L)	BQ (mg/L)	Acids (mg/L)
0	20.00	0.00	0.00	0.00	0.00	0.00
5	8.8	4.19	1.16	0.46	0.21	0.00
10	6.8	3.28	1.61	0.59	0.30	0.00
20	5.03	2.15	1.52	0.52	0.39	0.20
40	3.79	0.91	0.98	0.46	0.51	0.63
60	3.14	0.34	0.62	0.37	0.41	0.86
120	2.90	0.07	0.21	0.21	0.26	0.87

Table S3. Table of carbon equivalent.

Time (min)	TCP (mg C/L)	DCP (mg C/L)	CP (mg C/L)	Phenol (mg C/L)	BQ (mg C/L)	Acids (mg C/L)	TOC (mg C/L)
0	7.293	0.000	0.000	0.000	0.000	0.000	7.293
5	3.208	1.851	0.650	0.352	0.140	0.000	6.201
10	2.479	1.449	0.902	0.451	0.200	0.000	5.481
20	1.834	0.950	0.851	0.398	0.260	0.083	4.375
40	1.380	0.402	0.549	0.352	0.340	0.260	3.284
60	1.145	0.150	0.347	0.283	0.273	0.356	2.554
120	1.057	0.031	0.118	0.161	0.173	0.360	1.900

In lines 662-674, we added the paragraph: "To further clarify the relationship between TOC removal and the oxidation process, the temporal evolution of TOC and the concentrations of the major intermediates were investigated. As shown in Fig.7d, at the early stage of the reaction, the concentrations of dechlorination products, including DCP, CP, and phenol, initially increased, indicating that the cathodic reduction process first promoted the stepwise dechlorination of 2,4,6-TCP to form lower-chlorinated products and phenol. Subsequently, with the continuous generation of RCS, these dechlorinated intermediates were further oxidized, leading to the gradual formation of more oxidized products such as BQ and low-molecular-weight organic acids. As the reaction proceeded further, the concentrations of these oxidation intermediates also gradually decreased, accompanied by a continuous decline in TOC, indicating that the organics were further oxidized and eventually driven toward mineralization to form CO_2 and H_2O.".

Comment 1-3: Related to question 2, Why does the oxidation-only process lead to the accumulation of more toxic chlorinated intermediates, whereas the redox tandem system shows continuous toxicity reduction?

Response: We greatly appreciate your suggestion. For this question, we believe that the difference in toxicity evolution between the oxidation-only and tandem systems fundamentally arises from their distinct degradation sequences and intermediate profiles. As illustrated in the degradation pathway (Fig.7), in the oxidation-only system, 2,4,6-TCP is directly subjected to anodic oxidation while still retaining its highly chlorinated aromatic structure. Because the C–Cl bonds have not yet been sufficiently cleaved at this stage, the reaction is more likely to proceed through partial oxidation pathways, leading to the formation and accumulation of a series of chlorinated aromatic intermediates. Compared with non-chlorinated products, these partially oxidized but insufficiently dechlorinated intermediates generally retain relatively high toxicity, and the presence of residual C–Cl bonds also makes them less susceptible to further oxidative degradation. In some cases, such intermediates may even exhibit higher ecological risk due to the formation of more reactive structural motifs. Therefore, although the oxidation-only system can decrease the concentration of the parent pollutant, it does not necessarily lead

to continuous detoxification.

In contrast, the tandem system first promotes the cleavage of C–Cl bonds through cathodic hydrodechlorination, gradually converting 2,4,6-TCP into less chlorinated or even non-chlorinated intermediates, thereby weakening the toxicity associated with chlorinated structures at the source. These intermediates are then further oxidized by reactive chlorine species generated at the anode, making them more susceptible to ring opening and deep mineralization rather than remaining at the stage of toxic chlorinated byproducts. Therefore, the tandem system not only improves degradation efficiency, but more importantly reconstructs the transformation pathway of the pollutant, shifting it from the accumulation of highly chlorinated partially oxidized intermediates to a process of dechlorination-first detoxification followed by deep oxidation and mineralization, thereby resulting in a more continuous reduction in toxicity.

In lines 608-612, we added the sentence: "These results indicate that oxidation-only and redox tandem follow distinct transformation sequences: oxidation-only directly oxidizes highly chlorinated substrates, whereas the tandem process first removes chlorine substituents through cathodic hydrodechlorination and then oxidizes the dechlorinated intermediates.".

In lines 645-648, we added the sentence: "Because oxidation occurs before sufficient dechlorination in the oxidation-only pathway, many intermediates still retain chlorinated aromatic structures, which are less favorable for deep oxidation and therefore more likely to accumulate during the reaction.".

In lines 653-655, we added the sentence: "In contrast, the tandem process reconstructs the pathway into a dechlorination-first route, thereby suppressing the formation and persistence of toxic chlorinated oxidation intermediates.".

Comment 1-4: Also relevant to question 2 and as mechanistic consideration. What differences in degradation pathways account for incomplete mineralization in reduction-only and oxidation-only systems compared to the tandem process? This is important point to delineate in the manuscript as the author claim tandem setup provides superior advantage over the simple reduction or oxidation approach.

Response: Thank you very much for raising this issue. The incomplete mineralization in the reduction-only and oxidation-only systems originates from their distinct pathway limitations.

In the reduction-only system, cathodic hydrodechlorination efficiently removes chlorine substituents but largely preserves the aromatic ring, so the reaction mainly stops at phenolic intermediates rather than proceeding to deep mineralization. In the oxidation-only system, oxidation occurs before sufficient dechlorination, leading to the formation and accumulation of chlorinated benzoquinones and chlorinated ring-opening products, which are less favorable for complete mineralization. By contrast, the tandem system reorganizes the degradation sequence into dechlorination first and oxidation afterward. This pathway first lowers the chlorination level and then facilitates subsequent ring-opening and mineralization of the dechlorinated intermediates, thereby overcoming the intrinsic limitations of the individual reduction-only and oxidation-only processes.

In lines 639-641, we added the sentence: "Because this pathway mainly removes chlorine substituents but does not efficiently cleave the aromatic ring, mineralization remains incomplete and the reaction terminates predominantly at phenolic intermediates.".

In lines 643-646, we added the sentence: "Because oxidation occurs before sufficient dechlorination in the oxidation-only pathway, many intermediates still retain chlorinated aromatic structures, which are less favorable for deep oxidation and therefore more likely to accumulate during the reaction.".

In lines 651-653, we added the sentence: "In contrast, the tandem process reconstructs the pathway into a dechlorination-first route, thereby suppressing the formation and persistence of toxic chlorinated oxidation intermediates.".

Comment 1-5: How does the relative contribution of Cl· radicals compare with ·OH radicals in driving pollutant degradation in the tandem system? This is an important point to consider as OH radical is an integral part of advanced oxidation process (AOP) and contribution of Cl vs. OH radical needs to be clearly established.

Response: We greatly appreciate the valuable suggestion and have further investigated this point. In response to this comment, we further combined competitive scavenging experiments with probe-based kinetic analysis to semi-quantitatively evaluate their relative contributions in the tandem system. Specifically, as shown in Fig.3g, the addition of nitrobenzene (NB), which efficiently quenches ·OH but has negligible effect on reactive chlorine species (RCS), led to only an 8.7% decrease in degradation efficiency. In contrast, the addition of MeOH, which

quenches both ·OH and Cl·, resulted in a much larger decrease of 40.6%. Based on this competitive scavenging analysis, the relative contributions of ·OH and Cl· in the quenchable radical-mediated pathway were estimated to be approximately 21.4% and 78.6%, respectively, indicating that Cl· contributes much more strongly than ·OH to pollutant degradation in the oxidation step. To further support this conclusion, probe-based kinetic analysis was performed to semi-quantitatively evaluate the apparent steady-state concentrations of the major reactive species. In the oxidation system without Cl^- and 2,4,6-TCP, no obvious RCS was detected. By contrast, in the Cl^--containing system, the apparent steady-state concentration of Cl· reached 6.37×10^{-13} M, which was more than three orders of magnitude higher than that of ·OH, further confirming the dominant role of Cl·. In addition, the relative contribution analysis derived from probe kinetics (Fig.3h) showed that Cl· accounted for the dominant fraction of the oxidation process, whereas the contributions of ·OH were comparatively minor. Taken together, these results clearly establish that, although ·OH is present in the tandem system, Cl· is the dominant reactive species driving pollutant degradation under the present conditions. The corresponding results and discussion have now been added to the revised manuscript and Supplementary Information.

The Fig.3 was revised into:

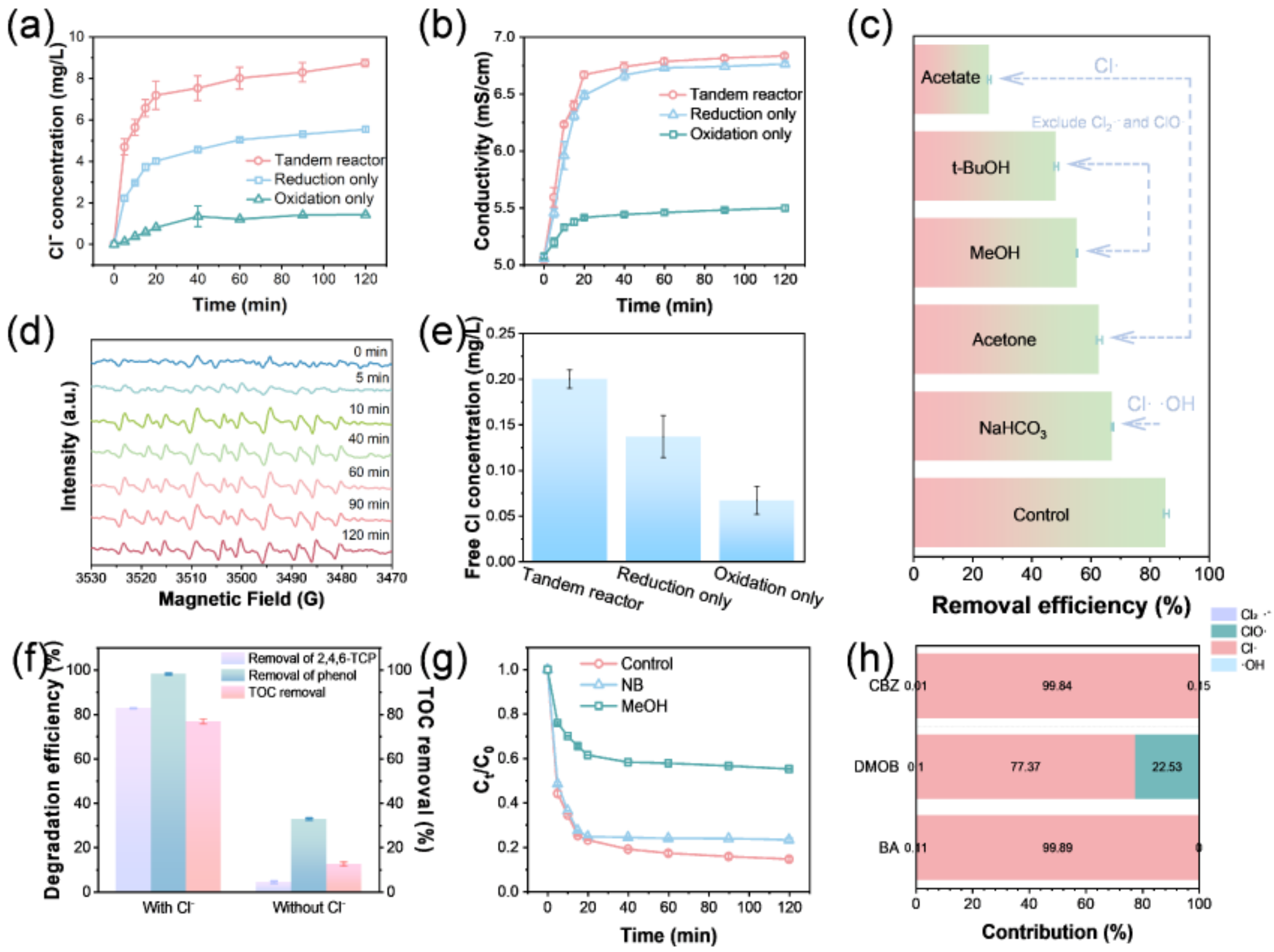

Fig.3 Evidence of the conversion of free chlorine ions to RCS. a,b, (a) Cl^- concentration and (b) Time-dependent variation in electrical conductivity in oxidation only, reduction only, and tandem reactor. **c,** 2,4,6-TCP quenching experiment in tandem reactor. **d,** EPR spectra of RCS trapped by PBN. **e,** Free Cl concentration in oxidation only, reduction only, and tandem reactor. **f,** The degradation efficiency and the TOC removal of simulated electrolyte after reduction-only reaction in the presence or absence of Cl^-. **g,** Quenching of ·OH: [MeOH] = [NB] = 5 mM. **h,** Relative radical contributions to the probe decay of BA, DMOB, and CBZ derived from probe-kinetic analysis.

The Table 1 was added into the revised manuscript:

	·OH (10^{-16} M)	Cl·(10^{-13} M)	ClO· (10^{-13} M)	$Cl_2^{\cdot-}$ (10^{-14} M)
Without Cl^-	8.92	0.00	0.00	0.00
With Cl^-	2.11	6.37	1.59	4.88

The Table S4 was added into the Suplementary Information:

species	$k_{\cdot OH+non\text{-}radical\ pathway}$ (10^{-4} min^{-1})	$k_{\cdot OH}$ (10^{-4} min^{-1})	$k_{non\text{-}radical\ pathway}$ (10^{-14} min^{-1})
NB	3.22	2.09	1.13
BA	6.91	3.16	3.75
DMOB	4.91	3.75	1.16
CBZ	8.94	4.71	4.23
TA	2.36	2.36	0.00

Table S5. The decay rates of NB, BA, CBZ and DMOB with Cl-.

k_{NB} (10^{-4} min^{-1})	k_{BA} (10^{-2} min^{-1})	k_{DMOB} (10^{-2} min^{-1})	k_{CBZ} (min^{-1})
1.62	6.92	8.90	1.26

In lines 459-478, we added the paragraph: "To further clarify the relative contributions of different reactive species during the oxidation process, both scavenging experiments and probe-based kinetic analysis were employed. As shown in Fig.3g, the addition of nitrobenzene (NB), a quencher that is highly reactive toward ·OH but has negligible effect on RCS, resulted in only an 8.7% decrease in degradation efficiency.[56] In contrast, the addition of MeOH, which can quench both ·OH and Cl·, caused a much larger decrease of 40.6%. This result indicates that RCS plays a more dominant role than ·OH in the oxidation step. Furthermore, NB, BA, CBZ, and DMOB were used as probe compounds to semi-quantitatively evaluate the apparent steady-state concentrations of ·OH, Cl·, ClO·, and $Cl_2^{\cdot-}$, respectively, as summarized in Table 1.[39] In the oxidation system without Cl^- and 2,4,6-TCP, no obvious RCS signal was detected. In contrast, in the Cl^--containing system, the apparent steady-state concentration of Cl· reached 6.37×10−13 M, which was markedly higher than that of ·OH, indicating the dominant role of Cl· in this system. The relative contribution analysis derived from probe kinetics (Fig.3h) further showed that Cl· accounted for the major fraction of the oxidation process, while

$ClO\cdot$ made a secondary contribution, and the roles of $Cl_2\cdot^-$ and $\cdot OH$ were comparatively minor. Taken together, these results demonstrate that the oxidation process in the tandem system was mainly driven by RCS, among which $Cl\cdot$ was the dominant reactive species.”.

In lines 865-906, we added the paragraph: “Based on previous studies, probe-based kinetic experiments were carried out under oxidation-only conditions to semi-quantitatively evaluate the relative contributions of different reactive species during the oxidation step.[39] A Cl^--free blank system and a Cl^--containing target system were established using 50 mM Na_2SO_4 as the supporting electrolyte, with the Cl^- concentration in the target system set to that measured in the post-reaction electrolyte of the reduction-only system. All other reaction conditions were kept identical to those used in the corresponding oxidation experiments. Terephthalic acid (TA, 0.5 mM) was used as the probe for $\cdot OH$, while nitrobenzene (NB), benzoic acid (BA), 1,4-dimethoxybenzene (DMOB), and carbamazepine (CBZ) were employed as stepwise discriminative probes for $\cdot OH$、$Cl\cdot$、$ClO\cdot$ and $Cl_2\cdot^-$, respectively, each at an initial concentration of 50 μM. Each probe was tested individually and was not co-present with the target pollutant. Their concentrations were determined by liquid chromatography, and the specific analytical method was adopted with reference to previous studies.[60] Aliquots were collected at predetermined time intervals, filtered, and then subjected to quantitative analysis. The apparent first-order rate constant, k_{obs}, for each probe was obtained by linear fitting according to:

$$ln\left(\frac{C_t}{C_0}\right)=-k_{obs}t \qquad (2)$$

t denoted the reaction time, C_t denoted the concentration at that time, and C_0 denoted the initial concentration of the probe.

In the Cl^--free blank system, the apparent steady-state concentration of $\cdot OH$ was first estimated from the apparent rate constant of TA:

$$[\cdot OH]_{SS}=\frac{k_{TA}}{k_{TA,\cdot OH}} \qquad (3)$$

where $k_{TA,\cdot OH}$ was the second-order reaction rate constant between TA and $\cdot OH$ (4.4×10^9 M^{-1} s^{-1})[61] and k_{TA} was the measured rate constant for TA oxidation in the blank system.

Subsequently, the decay rates of NB、BA、DMOB and CBZ measured in the blank system were used to subtract the non-radical loss term, $k_{non\text{-}radical}$, for each probe (Tables S4 and S5). In the Cl^--containing target system, the apparent rate constants of NB, BA, DMOB, and CBZ were further used in a stepwise subtraction manner to derive $[\cdot OH]_{SS}$, $[Cl\cdot]_{SS}$, $[ClO\cdot]_{SS}$ and$[ClO_2\cdot^-]_{SS}$. Specifically, NB was used to calculate $[\cdot OH]_{SS}$. BA was used to determine $[Cl\cdot]_{SS}$ after subtracting the contribution of ·OH. DMOB was used to calculate $[ClO\cdot]_{SS}$ after further subtracting the contributions of ·OH and Cl·, and CBZ was finally used to determine $[ClO_2\cdot^-]_{SS}$ after subtracting the contributions of the first three species.The results are summarized in Table S6. Since this method is based on apparent rate constants with background correction using the blank system, the obtained values are more appropriately interpreted as semi-quantitative descriptors of the relative roles of reactive species rather than strict absolute concentrations. The contributions of each free radical are calculated by the following formula:

$$Contribution_i = \frac{k_i}{\sum k_{radicals}} \times 100\% \quad (4)$$

i represented each free radical, and "contribution" indicated its relative contribution.”.

Comment 1-6: Although quenching experiments suggest that Cl· is the dominant reactive species, can the authors quantify the relative contributions of Cl· versus ·OH to overall degradation using kinetic modeling or competitive scavenging analysis?

Response: Thank you for this valuable suggestion. Accordingly, we performed a semi-quantitative competitive scavenging analysis. NB, which selectively quenches ·OH, caused only an 8.7% decrease in degradation efficiency, whereas MeOH, which quenches both ·OH and Cl·, caused a 40.6% decrease (Fig.3g). Based on this comparison, the relative contributions of ·OH and Cl· in the quenchable radical-mediated pathway were estimated to be ~21.4% and ~78.6%, respectively, indicating the dominant role of Cl·. This conclusion is further supported by probe-based kinetic analysis, which showed that the apparent steady-state concentration of Cl· was more than three orders of magnitude higher than that of ·OH in the Cl^--containing system. Together, these results provide semi-quantitative evidence that Cl·, rather than ·OH, is the dominant reactive species responsible for pollutant degradation in the tandem system.

Relevant discussion has been added to the revised manuscript and Supplementary Information. The Fig.3 was revised into:

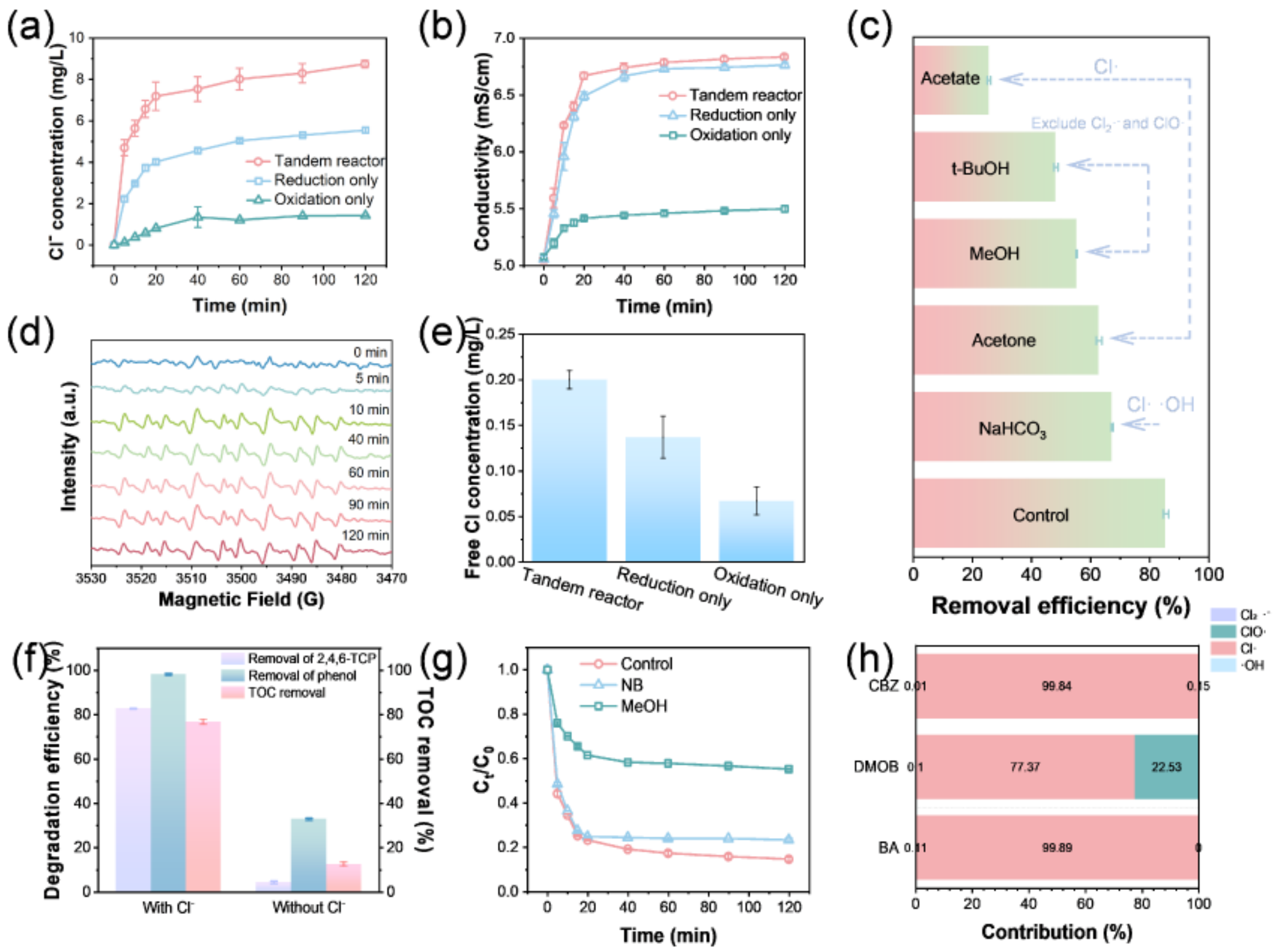

Fig.3 Evidence of the conversion of free chlorine ions to RCS. a,b, (a) Cl^- concentration and (b) Time-dependent variation in electrical conductivity in oxidation only, reduction only, and tandem reactor. **c,** 2,4,6-TCP quenching experiment in tandem reactor. **d,** EPR spectra of RCS trapped by PBN. **e,** Free Cl concentration in oxidation only, reduction only, and tandem reactor. **f,** The degradation efficiency and the TOC removal of simulated electrolyte after reduction-only reaction in the presence or absence of Cl^-. **g,** Quenching of ·OH: [MeOH] = [NB] = 5 mM. **h,** Relative radical contributions to the probe decay of BA, DMOB, and CBZ derived from probe-kinetic analysis.

The Table 1 was added into the revised manuscript:

	·OH (10^{-16} M)	Cl· (10^{-13} M)	ClO· (10^{-13} M)	$Cl_2^{·-}$ (10^{-14} M)

Without Cl^-	8.92	0.00	0.00	0.00
With Cl^-	2.11	6.37	1.59	4.88

The Table S4 was added into the Suplementary Information:

species	$k_{OH+non\text{-}radical\ pathway}$ (10^{-4} min^{-1})	k_{OH} (10^{-4} min^{-1})	$k_{non\text{-}radical\ pathway}$ (10^{-14} min^{-1})
NB	3.22	2.09	1.13
BA	6.91	3.16	3.75
DMOB	4.91	3.75	1.16
CBZ	8.94	4.71	4.23
TA	2.36	2.36	0.00

Table S5. The decay rates of NB, BA, CBZ and DMOB with Cl^-.

k_{NB} (10^{-4} min^{-1})	k_{BA} (10^{-2} min^{-1})	k_{DMOB} (10^{-2} min^{-1})	k_{CBZ} (min^{-1})
1.62	6.92	8.90	1.26

In lines 459-478, we added the paragraph: "To further clarify the relative contributions of different reactive species during the oxidation process, both scavenging experiments and probe-based kinetic analysis were employed. As shown in Fig.3g, the addition of nitrobenzene (NB), a quencher that is highly reactive toward ·OH but has negligible effect on RCS, resulted in only an 8.7% decrease in degradation efficiency.[56] In contrast, the addition of MeOH, which can quench both ·OH and Cl·, caused a much larger decrease of 40.6%. This result indicates that RCS plays a more dominant role than ·OH in the oxidation step. Furthermore, NB, BA, CBZ, and DMOB were used as probe compounds to semi-quantitatively evaluate the apparent steady-state concentrations of ·OH, Cl·, ClO·, and $Cl_2^{\cdot-}$, respectively, as summarized in Table 1.[39] In the oxidation system without Cl^- and 2,4,6-TCP, no obvious RCS signal was detected. In contrast, in the Cl^--containing system, the apparent steady-state concentration of Cl· reached

$6.37\times10{-}13$ M, which was markedly higher than that of ·OH, indicating the dominant role of Cl· in this system. The relative contribution analysis derived from probe kinetics (Fig.3h) further showed that Cl· accounted for the major fraction of the oxidation process, while ClO· made a secondary contribution, and the roles of $Cl_2^{\cdot-}$ and ·OH were comparatively minor. Taken together, these results demonstrate that the oxidation process in the tandem system was mainly driven by RCS, among which Cl· was the dominant reactive species.”.

In lines 865-906, we added the paragraph: “Based on previous studies, probe-based kinetic experiments were carried out under oxidation-only conditions to semi-quantitatively evaluate the relative contributions of different reactive species during the oxidation step.[39] A Cl^--free blank system and a Cl^--containing target system were established using 50 mM Na_2SO_4 as the supporting electrolyte, with the Cl^- concentration in the target system set to that measured in the post-reaction electrolyte of the reduction-only system. All other reaction conditions were kept identical to those used in the corresponding oxidation experiments. Terephthalic acid (TA, 0.5 mM) was used as the probe for ·OH, while nitrobenzene (NB), benzoic acid (BA), 1,4-dimethoxybenzene (DMOB), and carbamazepine (CBZ) were employed as stepwise discriminative probes for ·OH、Cl·、ClO· and $Cl_2^{\cdot-}$, respectively, each at an initial concentration of 50 μM. Each probe was tested individually and was not co-present with the target pollutant. Their concentrations were determined by liquid chromatography, and the specific analytical method was adopted with reference to previous studies.[60] Aliquots were collected at predetermined time intervals, filtered, and then subjected to quantitative analysis. The apparent first-order rate constant, k_{obs}, for each probe was obtained by linear fitting according to:

$$ln\left(\frac{C_t}{C_0}\right)=-k_{obs}t \tag{2}$$

t denoted the reaction time, C_t denoted the concentration at that time, and C_0 denoted the initial concentration of the probe.

In the Cl^--free blank system, the apparent steady-state concentration of ·OH was first estimated from the apparent rate constant of TA:

$$[\cdot OH]_{SS}=\frac{k_{TA}}{k_{TA,\cdot OH}} \tag{3}$$

where $k_{TA,\cdot OH}$ was the second-order reaction rate constant between TA and ·OH (4.4×10^9 M^{-1} s^{-1})[61] and k_{TA} was the measured rate constant for TA oxidation in the blank system. Subsequently, the decay rates of NB、BA、DMOB and CBZ measured in the blank system were used to subtract the non-radical loss term, $k_{non\text{-}radical}$, for each probe (Tables S4 and S5). In the Cl^--containing target system, the apparent rate constants of NB, BA, DMOB, and CBZ were further used in a stepwise subtraction manner to derive $[\cdot OH]_{SS}$, $[Cl\cdot]_{SS}$, $[ClO\cdot]_{SS}$ and$[ClO_2\cdot^-]_{SS}$. Specifically, NB was used to calculate $[\cdot OH]_{SS}$. BA was used to determine $[Cl\cdot]_{SS}$ after subtracting the contribution of ·OH. DMOB was used to calculate $[ClO\cdot]_{SS}$ after further subtracting the contributions of ·OH and Cl·, and CBZ was finally used to determine $[ClO_2\cdot^-]_{SS}$ after subtracting the contributions of the first three species.The results are summarized in Table S6. Since this method is based on apparent rate constants with background correction using the blank system, the obtained values are more appropriately interpreted as semi-quantitative descriptors of the relative roles of reactive species rather than strict absolute concentrations. The contributions of each free radical are calculated by the following formula:

$$Contribution_i = \frac{k_i}{\sum k_{radicals}} \times 100\% \quad (4)$$

i represented each free radical, and "contribution" indicated its relative contribution.”.

Comment 1-7: Can the authors quantitatively demonstrate the efficiency of Cl^- reutilization (e.g., percentage of released Cl^- converted to RCS versus remaining in solution), and how this correlates with enhanced mineralization? I suggest adding this control experiment to the manuscript to further elucidate this point.

Response: We fully understand and appreciate you raising this issue. First, based on the composition of the reduction-only electrolyte, we prepared a simulated solution containing the corresponding concentrations of 2,4,6-TCP and phenol, and compared the oxidation-only performance in the presence and absence of Cl^-. The results showed that the TOC removal efficiency increased markedly in the presence of Cl^-, demonstrating that Cl^--derived RCS made a major contribution to the subsequent mineralization step. Second, we further investigated the effect of externally added Cl^- concentration on TOC removal. As the Cl^- concentration

increased from 0 to 8 mg/L, the TOC removal efficiency continuously increased and finally reached 92.44%. This trend further demonstrates a clear positive correlation between the amount of available Cl^- and the extent of mineralization. Taken together, these two sets of results jointly support that the reutilization of released Cl^- is closely associated with enhanced mineralization, and that Cl^--enabled RCS generation is a key contributor to the superior performance of the tandem system.The relevant content and analysis have been included in the revised manuscript and Supplementary Information.

The Fig.3 was revised into:

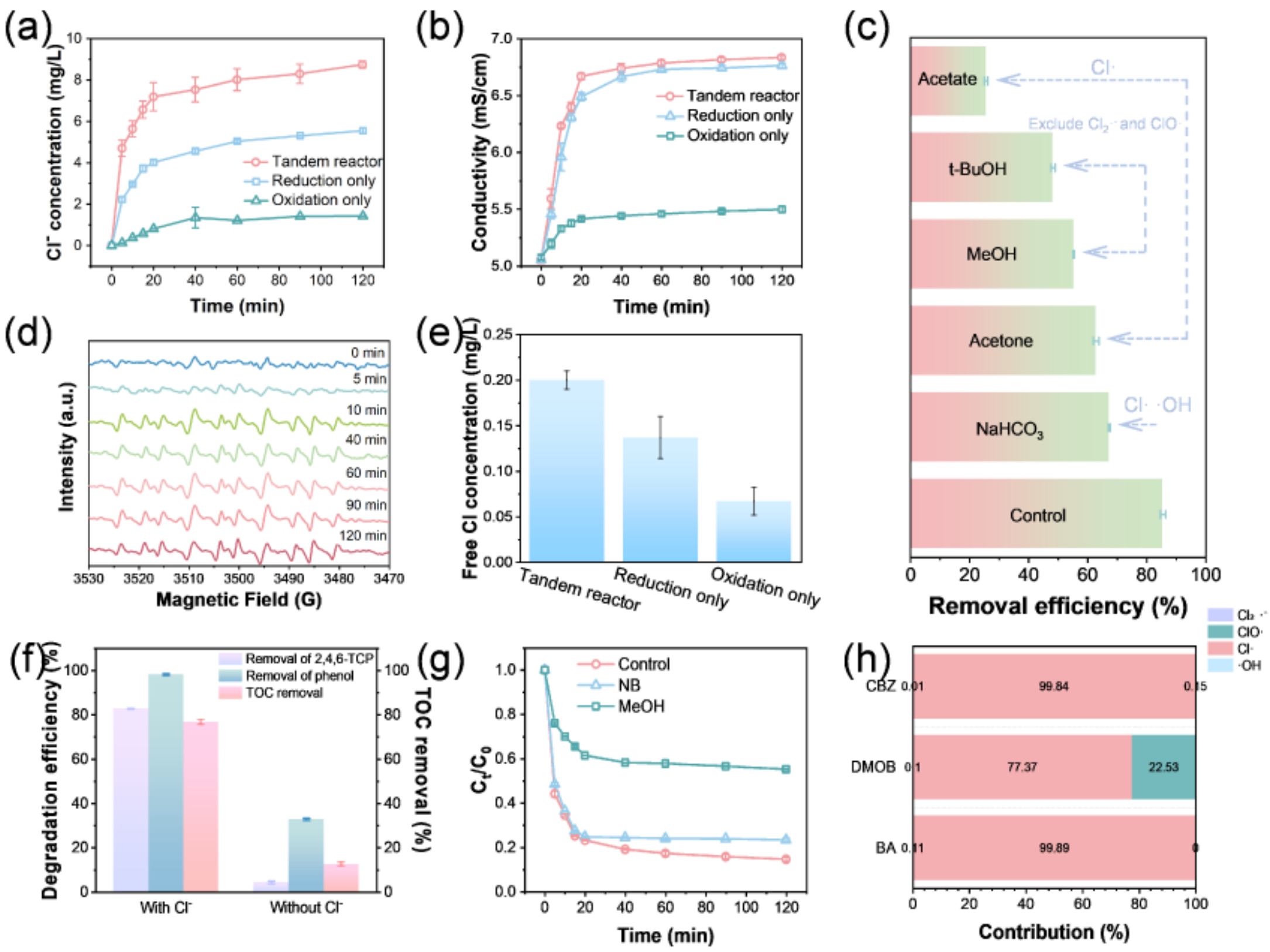

Fig.3 Evidence of the conversion of free chlorine ions to RCS. a,b, (a) Cl^- concentration and (b) Time-dependent variation in electrical conductivity in oxidation only, reduction only, and tandem reactor. **c,** 2,4,6-TCP quenching experiment in tandem reactor. **d,** EPR spectra of RCS trapped by PBN. **e,** Free Cl concentration in oxidation only, reduction only, and tandem reactor. **f,** The degradation efficiency and the TOC removal of simulated electrolyte after reduction-only reaction in

the presence or absence of Cl^-. **g,** Quenching of ·OH: [MeOH] = [NB] = 5 mM. **h,** Relative radical contributions to the probe decay of BA, DMOB, and CBZ derived from probe-kinetic analysis.

In lines 432-453, the paragraph "To semi-quantitatively evaluate the contribution of the RCS-mediated oxidation process to the overall performance enhancement of the system, the composition of the electrolyte after the reduction-only reaction was first analyzed, which mainly contained Cl^-, 2,4,6-TCP, and its representative dechlorinated intermediate, phenol. Based on this composition, a simulated electrolyte was further prepared, and oxidation-only experiments were conducted in the presence and absence of Cl^- to investigate the role of RCS-mediated oxidation in the overall system performance. As shown in Fig.3f, in the presence of Cl^-, the removal efficiencies of 2,4,6-TCP and phenol reached 82.8% and 98.3%, respectively, and the TOC removal efficiency reached 76.72%, which was comparable to the mineralization performance of the redox tandem system. In contrast, in the absence of Cl^-, the TOC removal efficiency was only 12.46%. This result indicates that the presence of Cl^- provided an additional 60.4 percentage points of TOC removal enhancement during the subsequent oxidation stage, strongly suggesting that Cl^--derived RCS play a dominant role in deep oxidation and mineralization. Combined with the reduction-only results, it can be inferred that sequential dechlorination mainly contributes to C–Cl bond cleavage, precursor transformation, and detoxification, whereas RCS-mediated oxidation is primarily responsible for the subsequent ring opening and deep mineralization. Therefore, the performance enhancement of the tandem system arises from the sequential synergy between these two processes, with RCS-mediated oxidation being the key contributor to TOC removal improvement." was added.

The Supplementary Fig.13 was added in supplementary information.

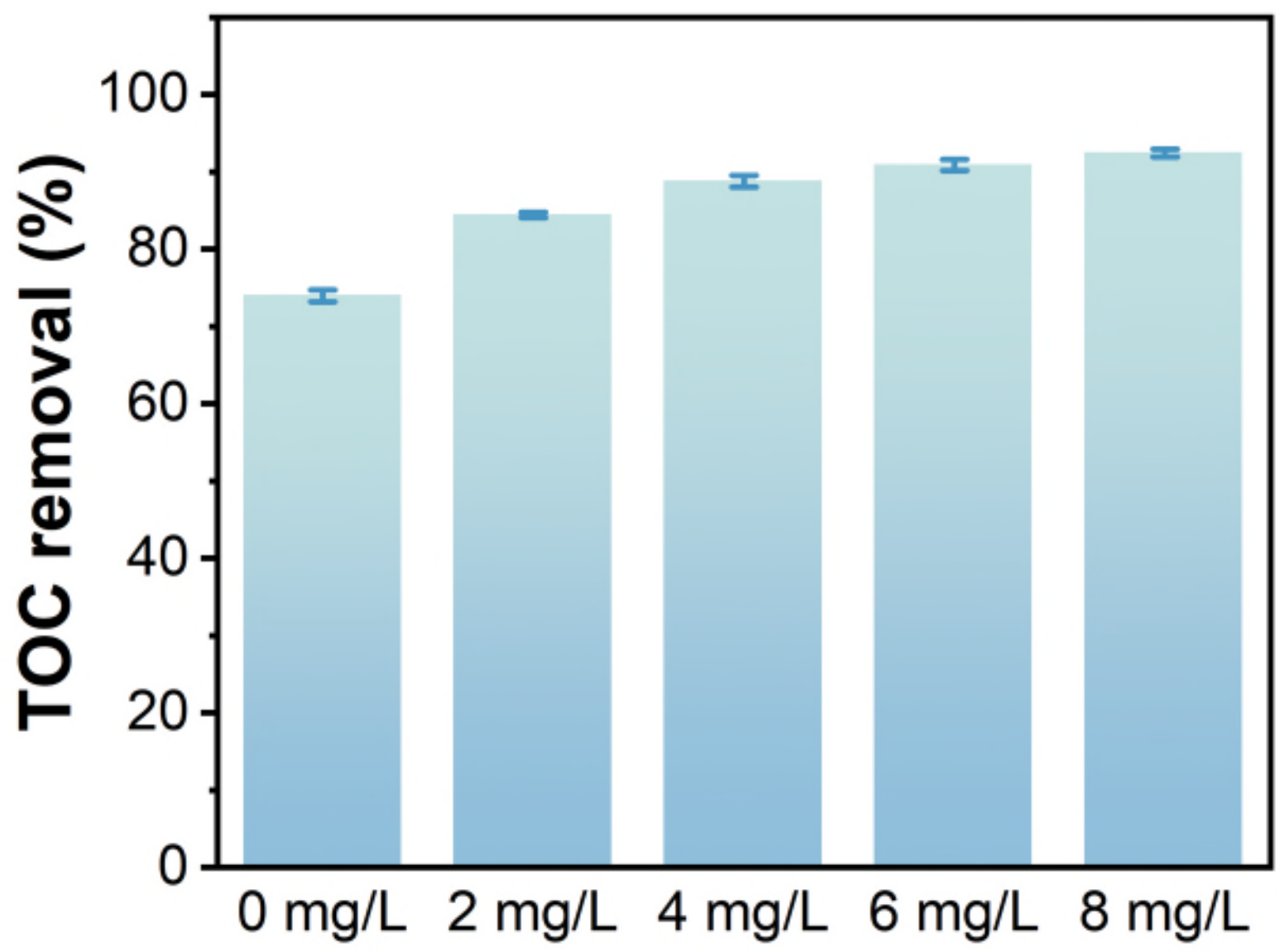

Fig.S12 The TOC removal of different additional Cl^- concentrations

In lines 453-458, the paragraph "With the addition of external Cl^- to the electrolyte, the TOC removal efficiency further increased from 73.97% to 92.45% as the chloride concentration increased (Supplementary Fig.12). This result suggests that a higher Cl^- concentration provides more available precursors for RCS generation, thereby promoting subsequent deep oxidation and enhancing the mineralization efficiency." was added.

Comment 1-8: What is the relationship between the increase in Cl^- concentration (reflected by conductivity changes) and the enhancement of redox synergy?

Response: Thank you for raising this important point. The increase in Cl^- concentration, as reflected by the conductivity change, is mechanistically associated with the enhancement of redox synergy because it indicates that more chloride released from cathodic hydrodechlorination becomes available for anodic reutilization into reactive chlorine species (RCS). In other words, as 2,4,6-TCP undergoes stepwise hydrodechlorination at the cathode, Cl^- is continuously liberated into solution, leading to an increase in conductivity. This increasing chloride pool is not merely a passive product reservoir; rather, in the tandem system,

it provides the precursor for anodic generation of secondary oxidants such as Cl·, Cl_2, and HOCl. As supported by the LSCM results (Fig.4), the accumulation and interfacial availability of Cl^- near the anode facilitate the formation of these RCS, which can further oxidize dechlorinated intermediates and residual organics, thereby promoting ring opening and deep mineralization. Therefore, the rise in Cl^- concentration reflects a strengthened coupling between the cathodic and anodic steps: cathodic dechlorination continuously supplies chloride, while the anode reactivates it into oxidative species, together enhancing the overall redox synergy. We also agree that conductivity alone is only an indirect indicator. Accordingly, in the manuscript, we interpret the conductivity change together with free-chlorine measurements, radical identification results, and degradation performance to collectively support the role of chloride reutilization in strengthening the tandem redox synergy.

In lines 361-364, the sentence was revised into "This behavior is mainly attributed to the continuous release of Cl^- during the cleavage of C-Cl bonds in 2,4,6-TCP, since Cl^- possesses a relatively high molar conductivity and therefore contributes significantly to the increase in solution conductivity.".

In lines 364-366, the sentence was revised into "Therefore, the conductivity increase can be regarded as an indirect indicator of Cl^- accumulation originating from pollutant dechlorination rather than merely a bulk solution property change.".

Comment 1-9: How do the formation and concentration of free chlorine differ among oxidation-only, reduction-only, and tandem systems, and how does this influence overall mineralization efficiency?

Response: Thank you for this important question. The formation and role of free chlorine differ substantially among the oxidation-only, reduction-only, and tandem systems. In the oxidation-only system, free chlorine can be generated at the anode, but its overall formation is limited by the lack of a continuous chloride supply released from cathodic dechlorination. In the reduction-only system, chloride ions are continuously released through C–Cl bond cleavage, but they are not further converted into oxidation-active chlorine species because the anodic oxidation step is absent or ineffective. As a result, chloride mainly accumulates in solution, while mineralization remains limited. By contrast, in the tandem system, cathodic dechlorination

continuously supplies Cl^-, which can then be further activated at the anode into free chlorine and other reactive chlorine species. Therefore, the tandem system establishes a dynamic chloride reutilization loop. It should be emphasized that the steady-state free chlorine concentration does not directly represent the total oxidative contribution of chlorine-derived species, because free chlorine and related RCS are continuously consumed by organics and intermediates once formed. Thus, the superior mineralization efficiency of the tandem system is not simply due to a higher static free chlorine concentration, but rather to the continuous generation and effective utilization of chlorine-derived oxidizing species, together with the prior stepwise dechlorination that makes the intermediates more susceptible to further oxidation.

Comment 1-10: Cl_3^- is a known chloride oxidation pathway in chloride-containing electrochemical systems via the equilibrium $Cl_2+Cl^-\rightleftharpoons Cl_3^-$. What thermodynamic or interfacial arguments support the preferential formation of Cl_2 (or radical species such as Cl·) over Cl_3^- in this cascade reactor, and why would Cl_3^- formation be less favorable under the reported operating conditions?

Response: Thank you to the reviewer for raising this insightful question. We agree that Cl_3^- can in principle form through the solution-phase equilibrium $Cl_2 + Cl^- \rightleftharpoons Cl_3^-$. However, under the operating conditions of the present cascade reactor, its significant accumulation is unlikely to be favored. The reasons are as follows. First, the system operates under acidic conditions, under which anodic chlorine chemistry is more likely to proceed via interfacial Cl^- activation and initial Cl_2 formation, followed by transformation into free-chlorine species such as $HOCl/ClO^-$. From this reaction pathway, Cl_2/free chlorine is therefore more reasonably considered the dominant chlorine-derived species than Cl_3^- (Liu, Y. et al. Nat. Commun. 2023, 14, 2475). Second, chloride-involved electrochemical oxidation is governed primarily by interfacial Cl^- adsorption and activation at the electrode surface. Accordingly, the initial anodic process is more likely to involve adsorbed chlorine intermediates that subsequently evolve into Cl_2/free chlorine. In contrast, Cl_3^- is better understood as a solution-phase association species formed by the equilibrium between dissolved Cl_2 and Cl^- in the bulk phase, rather than as a preferential primary interfacial oxidation product (Duan, Y. et al. Nat. Commun. 2024, 15, 1973). Third, the present system is fundamentally different from concentrated brine or chlorine-storage systems.

No additional chloride was introduced into the supporting electrolyte, and the Cl^- in our reactor originates exclusively from pollutant dechlorination. As a result, the system operates under dilute-chloride and continuously consumptive conditions, in which newly generated chlorine-derived oxidants are expected to be rapidly consumed in the subsequent oxidation of organics and intermediates, making substantial bulk accumulation of Cl_3^- less favorable (A. Cerquetti. et al. J. Electroanal. Chem. Interfacial Electrochem. 1969, 20, 3, 411-418). Based on these considerations, although we cannot completely exclude the possibility of trace Cl_3^- formation in local microenvironments, we believe that under our operating conditions, the species more relevant to the observed tandem oxidation behavior are Cl_2/free chlorine and chlorine-radical-related reactive species, rather than Cl_3^-.

1. Liu, Y. et al. Electrosynthesis of chlorine from seawater-like solution through single-atom catalysts. Nat. Commun. 14, 2475 (2023).

2. Duan, X. et al. Dynamic chloride ion adsorption on single iridium atom boosts seawater oxidation catalysis. Nat. Commun. 15, 1973 (2024).

3. Cerquetti, A., Longhi, P., Mussini, T. & Natta, G. Thermodynamics of the $Cl_2/Cl^-/Cl_3^-$ system in aqueous solution. J. Electroanal. Chem. Interfacial Electrochem. 20, 411–418 (1969).

Comment 1-11: Have the authors conducted control experiments under open-circuit conditions or without applied potential to exclude possible adsorption, non-electrochemical degradation, or spontaneous chemical reactions contributing to 2,4,6-TCP removal? If so, please provide the corresponding degradation and TOC data to confirm that the observed performance arises solely from the electrochemical processes.

Response: Thank you for this important comment. To exclude the possible contribution of adsorption, non-electrochemical degradation, and spontaneous chemical reactions, control experiments were conducted without applied potential using the same reactor configuration and solution composition. Under the condition, 2,4,6-TCP removal was only 6.8%, while the corresponding TOC removal (0.54%) was much negligible compared with that under electrochemical operation. This indicates that, although a minor contribution from adsorption or non-electrochemical transformation cannot be completely excluded, the substantial enhancement in degradation and especially mineralization observed in the tandem system mainly arose from the electrochemical processes. The corresponding data have now been added

to the revised manuscript and the supplementary information.

The Supplementary Figs. 7 and 8 was added in supplementary information.

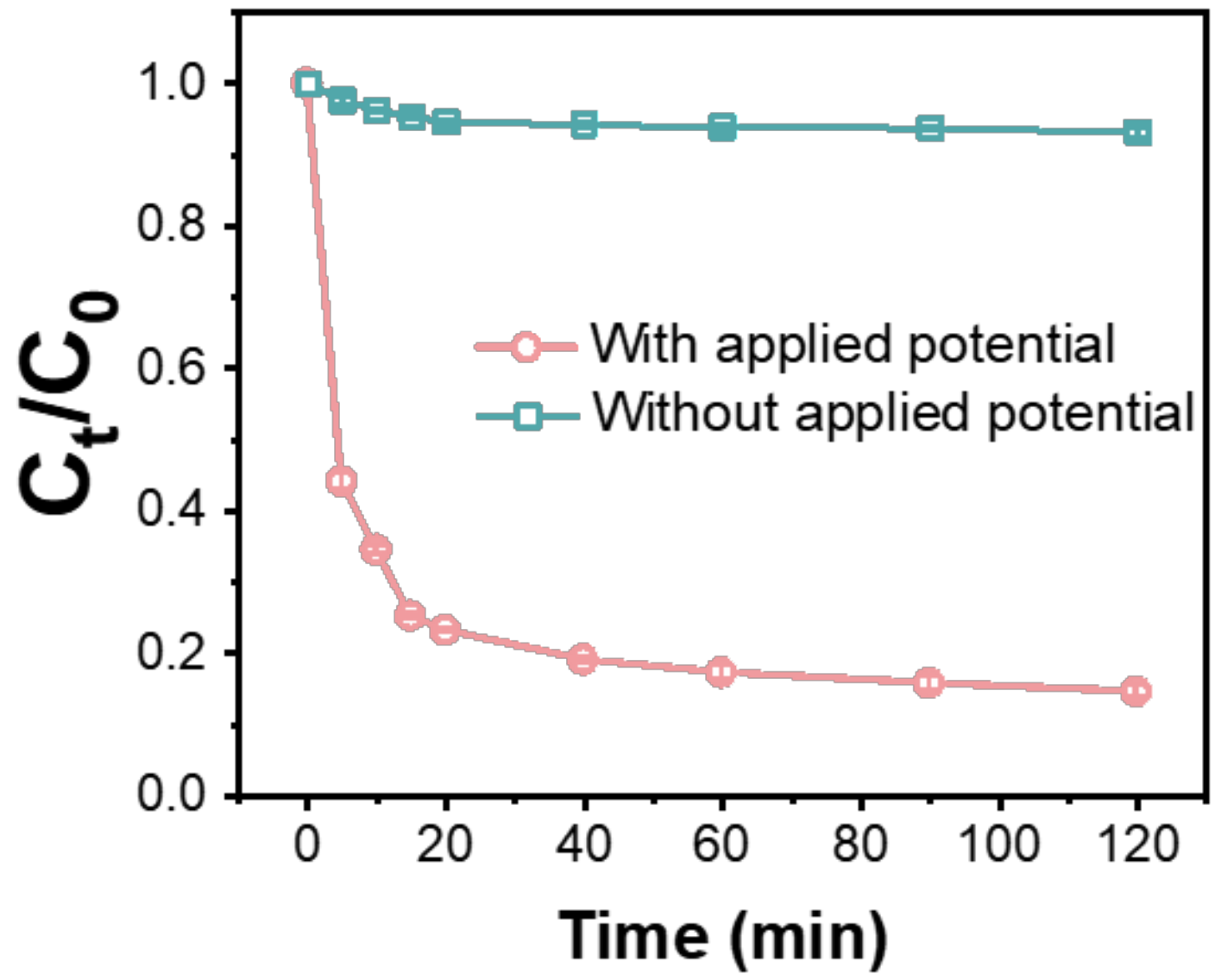

Fig.S7 The degradation efficiency of 2,4,6-TCP with and without applied potential.

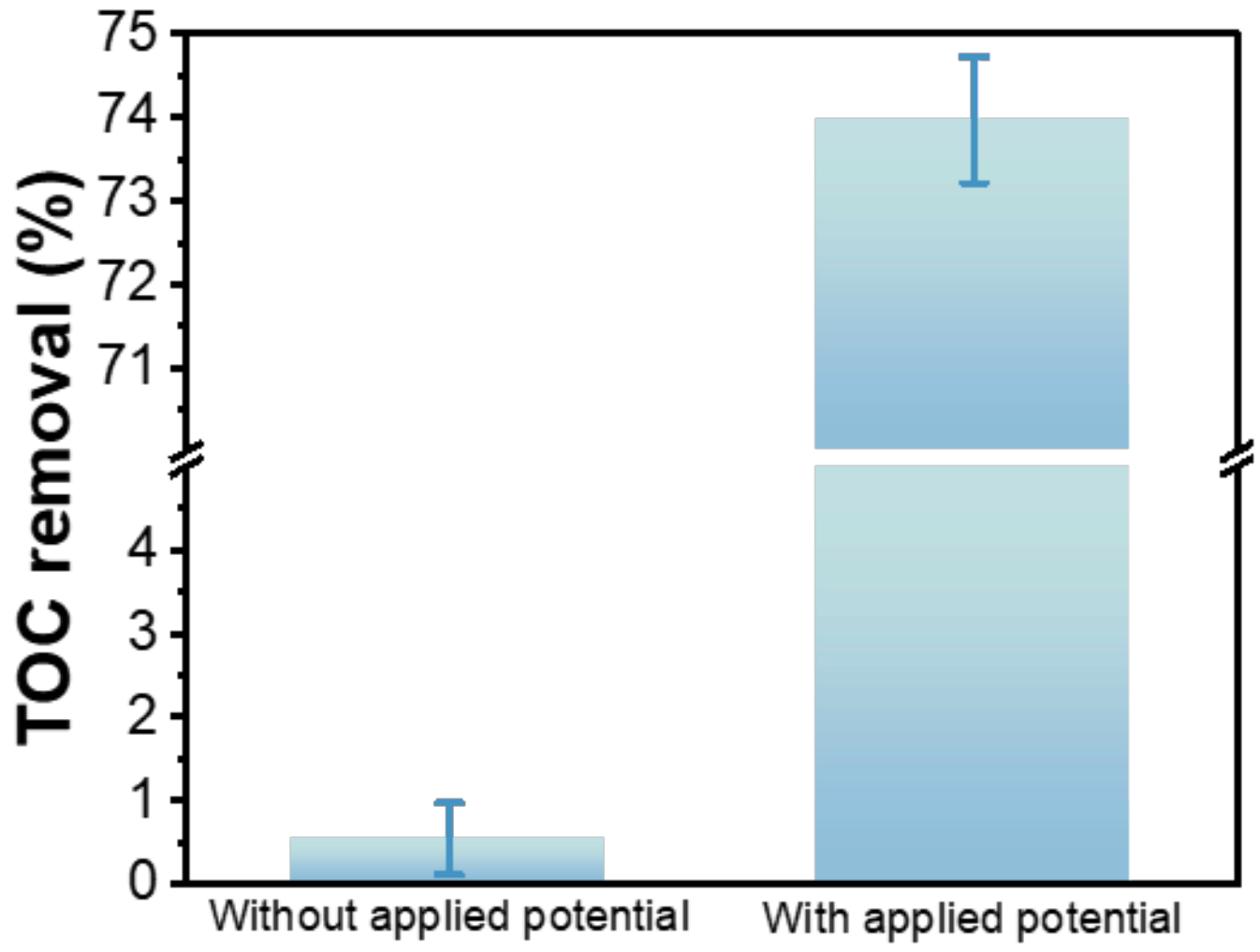

Fig.S8 The TOC removal of 2,4,6-TCP with and without applied potential.

In lines 258-265, the sentence "Subsequently, experiments were carried out without applied potential. Under this condition, the removal efficiency of 2,4,6-TCP was only 6.8%, and the corresponding TOC removal efficiency was merely 0.54%, both of which were negligible

compared with the performance obtained at 1.5 V (Supplementary Figs. 7 and 8). These results indicate that the degradation observed in the tandem system mainly originated from the electrochemical process rather than from adsorption or other non-electrochemical effects." was added.

Reviewer 2:

The manuscript developed a cascade electrochemical reactor for removing chlorinated organic pollutants (COPs) from water bodies. It proposed an innovative research direction that converts removed chloride ions into reusable active chlorine species. The topic is of significant importance and the manuscript was well prepared with plenty of data and insightful discussions. This work is publishable after the following comments were addressed well.

Response:

We greatly appreciate your thoughtful evaluation of our work and your patience in offering such detailed and constructive suggestions. Guided by your insightful comments, we have carefully revised the manuscript to improve its quality and clarity. The specific changes and corresponding responses are provided below.

Comment 2-1: While the manuscript proposes a strategy to prevent catalyst poisoning by reusing chloride ions, sufficient comparison and differentiation from existing literature should be provided.

Response: Thanks to your reasonable and scientific advice. In the revised manuscript, we have further strengthened the comparison with the existing literature. Unlike conventional electrocatalytic dechlorination studies, which mainly focus on improving cathodic dechlorination efficiency and often regard the released Cl^- as a byproduct or even a potential source of electrode contamination/poisoning, the present work further converts the released Cl^- in situ at the anode into RCS for subsequent oxidation reactions. This strategy not only enables the reutilization of chloride ions, but also helps avoid their accumulation in the system and the resulting adverse effects on electrode active sites, thereby highlighting the distinctive advantage of the tandem redox system over existing studies. The changes are shown below.

In lines 83-90, the sentence was revised into "More importantly, in conventional EHDC systems,

the released Cl^- is commonly regarded as a detached byproduct and a potential source of active-site poisoning, and previous studies have mainly addressed this issue by improving the chloride tolerance of Pd-based cathodes through catalyst design or additives. In contrast, the present tandem strategy prevents persistent chloride accumulation by further converting the released Cl^- in situ at the anode, thus offering a process-level advantage in mitigating electrode poisoning beyond conventional cathode-only optimization.[10, 17]".

Comment 2-2: The work investigates the superiority of the redox tandem by simulating reduction-only and oxidation-only conditions using pure carbon paper electrodes for the cathode and anode, respectively. The purpose of the experiment using a proton exchange membrane to separate the anode and cathode chambers should be further explained in the manuscript.

Response: Thank you for this insightful comment. The proton exchange membrane was introduced to physically separate the anodic and cathodic chambers so that the oxidation-only and reduction-only experiments could more accurately reflect the intrinsic contribution of each half-reaction. This configuration minimizes the crossover of reactive intermediates and products between the two electrodes, thereby preventing mutual interference between cathodically generated reducing species and anodically generated oxidizing species. In addition, pure carbon paper was used to replace the cathode or anode, respectively, in the control experiments to eliminate the catalytic contribution of the corresponding functional electrode. In this way, the reduction-only and oxidation-only systems could be used to isolate the individual role of the PdCo/CP cathode and the RuO_2/Ti anode, respectively. Therefore, the membrane-separated configuration together with the carbon-paper substitution controls provided a more rigorous basis for comparing the individual oxidation/reduction pathways with the tandem redox system and for evaluating the synergistic advantage of the coupled process. We have clarified this point in the revised manuscript.

In lines 274-277, the sentence was revised into "The proton exchange membrane can minimize the crossover of reactive intermediates between the two electrodes, so that the individual contribution of the RuO_2/Ti anode and PdCo/CP cathode could be evaluated separately.".

In lines 295-297, the sentence was revised into "Subsequently, the cathode and anode within

the cascade reactor were replaced with carbon paper to investigate the specific contribution of oxidation-only and reduction-only.”.

Comment 2-3: Has the mechanism by which chloride ions are converted into active chlorine species (such as HOCl or Cl·) been fully elucidated? Any supporting data for this mechanism?

Response: Thank you for this insightful comment. We agree that, in the present study, the exact elementary steps underlying the conversion of Cl^- into RCS have not been fully resolved at the atomic level. To avoid overstatement, we have revised the relevant statements in the manuscript and added a clearer mechanistic discussion. Specifically, the Cl^- released during cathodic dechlorination is proposed to migrate toward the anode interface, where it undergoes interfacial enrichment and is subsequently oxidized into RCS, with Cl· serving as one of the key reactive species in this system. This proposed pathway is supported by multiple lines of evidence. First, the EPR spectra show that the characteristic signal of Cl· becomes progressively more pronounced with increasing reaction time, indicating the continuous generation of chlorine radicals during the reaction. Second, comparative quenching experiments using different scavengers further confirm the involvement of RCS in the oxidation process. Third, LSCM results reveal that the Cl^- signal on the anode surface decreases rapidly within 5 s, suggesting fast interfacial consumption of Cl^-, which is consistent with their conversion into RCS. In addition, AIMD simulations show that Cl^- is located closer to the anode surface than H_2O, implying that chloride is more likely to be activated into RCS than H_2O is to be oxidized into ·OH and related oxygenated radicals under the present conditions. Taken together, these results support the proposed anodic reutilization of released chloride and its further conversion into RCS for subsequent oxidation reactions.

In lines 385-391, the sentence was revised into “In addition, the reaction rate constant of acetate with Cl· is significantly higher than that of acetone with Cl·, whereas both compounds exhibit comparable reactivity toward ·OH.[39] As shown in Fig. 3c, acetate caused a much stronger inhibition of degradation than acetone, highlighting the important role of Cl· in the oxidation process. These results indicate that released Cl^- in the solution were converted into RCS for oxidation, with Cl· being the primary contributor throughout the oxidation process.”.

In lines 369-376, the sentence "Previous studies suggest that anodic chloride activation proceeds through interfacial enrichment/adsorption of Cl^- followed by the formation of adsorbed chlorine intermediates (e.g., Cl or OCl). Depending on the interfacial binding strength and local reaction environment, these intermediates may either evolve toward Cl_2/HOCl or further generate chlorine radicals. Therefore, in the present system, the released Cl^- is proposed to be activated on the RuO_2/Ti anode through adsorbed chlorine intermediates, with a radical-dominated RCS pathway favored under the present conditions.[47, 48]" was added.

In lines 403-404, the sentence was revised into "This suggests that a small amount of non-radical active chlorine species also coexisted in the reaction system.".

In lines 520-527, the sentence was revised into "This interfacial accumulation also implied that Cl^- was not merely transported to the anode surface, but was further electrochemically consumed there. Previous studies have shown that under anodic polarization, interfacial Cl^- can dynamically adsorb on active sites and be converted into adsorbed chlorine intermediates (e.g., M–Cl, Cl or OCl), which subsequently evolve into RCS. Therefore, the local decrease in Cl^- concentration observed at the anode surface during 5–15 s was reasonably attributed to its interfacial oxidation and conversion into RCS, rather than simple redistribution in the electrolyte.[58]".

Comment 2-4: Since the PBN was used to capture chlorine radicals in the experiment, the specific conditions should be provided. It is recommended to supplement with detailed experimental parameters, such as PBN concentration and reaction duration.

Response: We apologize for this oversight and thank the reviewer for bringing this issue to our attention. In the revised manuscript, we have added the following details to the Method section regarding the EPR measurements.

In lines 811-817, the sentence was revised into "EPR spectra were recorded on a Bruker EMXplus-9/12 spectrometer. PBN (100 mM) and DMPO (100 mM) were employed as spin-trapping agents for reactive chlorine species (RCS) and H*, respectively. The trapping agent was first introduced into the electrolyte before electrolysis. For H* analysis, the sample was taken after 5 min of reaction and immediately subjected to EPR measurement. For Cl· analysis, samples were collected at predetermined reaction intervals for EPR detection.".

Comment 2-5: The AIMD section mentions the helmholtz layer without discussion on its impact. Further explanation is recommended.

Response: Thank you very much for pointing out this issue. In the revised manuscript, we have added a relevant explanation to clarify the role and relevance of the Helmholtz layer in this system. The Helmholtz layer represents the compact electrode/electrolyte interfacial region, where the ion distribution and solvent configuration differ from those in the bulk phase and may therefore influence interfacial adsorption and reaction kinetics. In the present system, the Helmholtz layer is mentioned mainly to indicate that Cl^- near the anode surface is more likely to undergo interfacial enrichment and reorganization and, compared with H_2O, is more readily activated to generate reactive species, thereby affecting its anodic conversion behavior and participation in the reaction.

In lines 575-583, the sentence was revised into "As a specifically adsorbable reactant, Cl^- is located much closer to the anode, with a preferred distance of approximately 1.41 Å, indicating its enrichment within the inner Helmholtz layer. In contrast, H2O molecules are distributed farther from the surface, mainly in the outer Helmholtz layer and diffusion layer, with distances predominantly exceeding 9.35 Å. This distinct interfacial configuration suggests that Cl^- has a greater probability of directly interacting with anodic active sites than H2O, thereby favoring the CER over the competing OER. As a result, the interfacial enrichment of Cl^- in the Helmholtz layer facilitates the generation of RCS for subsequent oxidation reactions.".

Comment 2-6: Why is pH 4 set as the reaction condition? Any basis or supporting evidence?

Response: We thank you for this insightful comment. According to previous studies, acidic conditions are generally more favorable for the generation of H* on the cathode and RCS on the anode (Zhang, Y. et al. Nat. Commun. 2025, 16, 10863; Liu, Y. et al. Nat. Commun. 2023, 14, 2475; Wu, B. et al. Nat. Commun. 2025, 16, 166). Specifically, in acidic media, H^+ can be directly reduced to H*, thus avoiding the additional water-dissociation step required in neutral or alkaline electrolytes and resulting in more favorable interfacial kinetics. In line with this, our previous work also indicated that cathodic reduction performed more effectively at pH 4 (Deng, C. et al. J. Colloid Interface Sci. 2025, 696, 137877). In addition, the speciation of active

chlorine is strongly pH-dependent. Under acidic conditions, the system is more likely to form Cl_2 and HOCl, whereas under alkaline conditions, the equilibrium shifts predominantly toward OCl^-. Compared with an OCl^--dominated environment, a Cl_2/HOCl-enriched interface is generally considered more favorable for the generation of Cl• and the subsequent oxidation pathway mediated by RCS. Therefore, pH 4 was chosen in this study to better promote the tandem process. This explanation has been added to the revised manuscript.

In lines 833-835, the sentence "Based on previous studies of the cathodic reduction process, pH 4 was selected as the reaction condition in this study.[18]" was added.

1. Deng, C. et al. Electronic structure modulation on PdCo bimetal for enhanced atomic hydrogen mediated electro-dehalogenation of 2,4,6-trichlorophenol. J. Colloid Interface Sci. 696, 137877 (2025).

Comment 2-7: Some sentences contain spelling and grammatical typos that may compromise the paper's professionalism and readability. It is suggested that the author carefully review the entire manuscript and eliminate unnecessary spaces and formatting errors.

Response: We sincerely thank the reviewer for this helpful comment. We have carefully re-examined the entire manuscript and corrected the spelling, grammar, and formatting errors throughout the text. Unnecessary spaces have also been removed. These revisions have improved the clarity, readability, and overall presentation of the manuscript.

Reviewer 3:

This manuscript proposes a cascade electrochemical system that couples cathodic hydrodechlorination with anodic chlorine radical generation, enabling in situ reutilization of Cl^- for deep mineralization of chlorinated pollutants.

The manuscript claims a "new strategy", but redox coupling (ECH + oxidation) is already widely reported (see refs 5, 9, etc.). And, chloride-mediated oxidation (RCS/CER) is also well established. So the true novelty (chloride self-recycling) is incremental and not clearly distinguished from, electro-Fenton + halide systems, paired electrolysis and self-generated oxidant systems.

There is insufficient quantitative evidence for Cl^- → RCS reutilization. This is the central claim, but evidence is indirect, e.g., EPR confirms Cl· presence, but not its origin (from released Cl^- vs electrolyte/background); No isotope labeling or mass balance; Free chlorine measured is very low (~0.2 mg/L, page 19). Chlorine mass balance, FE toward CER vs OER, as well as quantitative RCS yield are missing.

Based on the current shape of the manuscript, I would recommend a major revision for the subsequent consideration of publication.

Response:

We sincerely appreciate the insightful comments and fully acknowledge the concern regarding the novelty of our proposed system, particularly given that redox coupling (reduction coupled with oxidation) and chloride-mediated oxidation are both established strategies in the literature. We would like to clarify that the novelty of our work does not lie in simply combining reductive and oxidative processes in a tandem configuration. Rather, the key innovation is the in situ self-recycling and valorization of chloride ions released from cathodic hydrodechlorination.

Specifically, in conventional electroreductive dechlorination processes, the released Cl^- is generally regarded as a terminal byproduct or even a secondary waste stream. In contrast, in our cascade electrochemical system, these chloride ions are not left as inert residues; instead, they are reutilized in situ at the anode and converted into reactive chlorine species (RCS), which subsequently participate in the further oxidation of dechlorinated intermediates and residual organics. In this way, the chlorine originally bound in chlorinated pollutants is transformed from a leaving group into a functional oxidative mediator, thereby enabling deep mineralization rather than partial detoxification alone. Therefore, the essence of our contribution is not merely a redox tandem process, but a closed-loop chloride reutilization strategy that converts dechlorination-derived Cl^- from a secondary waste into a valuable reactive resource.

We agree that more direct and quantitative evidence for the Cl^- → RCS reutilization pathway is essential to support this central claim. In the original manuscript, such evidence was not sufficiently developed. In response to the reviewer's suggestion, we have now incorporated additional analyses and discussion in the revised manuscript to more clearly substantiate the chloride self-recycling mechanism and reinforce the novelty of this work.

Comment 3-1: How do you prove that RCS originates from released Cl^- rather than

background electrolyte or electrode processes?

Response: Thank you very much for raising this critical question. We would like to further clarify the origin of the RCS observed in our system. In this study, the background electrolyte consisted of only 50 mM Na_2SO_4, and no chloride-containing species were introduced into the supporting electrolyte. Therefore, the background electrolyte itself did not provide any chlorine source. Under this condition, the only possible source of the detected Cl· and related RCS is the Cl^- released during the cathodic hydrodechlorination of 2,4,6-TCP. In other words, the pollutant itself served as the sole chlorine source in the present system. Accordingly, the observed generation of RCS can be directly attributed to the chloride ions liberated from pollutant dechlorination, rather than to the background electrolyte or unrelated electrode processes. To further support this conclusion, we have highlighted the control experiment conducted in the absence of pollutant. When the system contained only the 50 mM Na_2SO_4 background electrolyte and was operated for 10 min under otherwise identical conditions, no corresponding chlorine-radical signal was detected. This result further confirms that the appearance of RCS is directly associated with the dechlorination of 2,4,6-TCP. Based on this experimental design, we believe that isotope labeling is not a prerequisite for identifying the chlorine origin in the present system, because the chlorine source is already uniquely defined by the system composition itself. We have further clarified this point in the revised manuscript to make it clearer that the observed RCS originate from the Cl^- released during pollutant dechlorination, rather than from the background electrolyte or other electrode-related processes. The Supplementary Fig.10 was added in Supplementary Information:

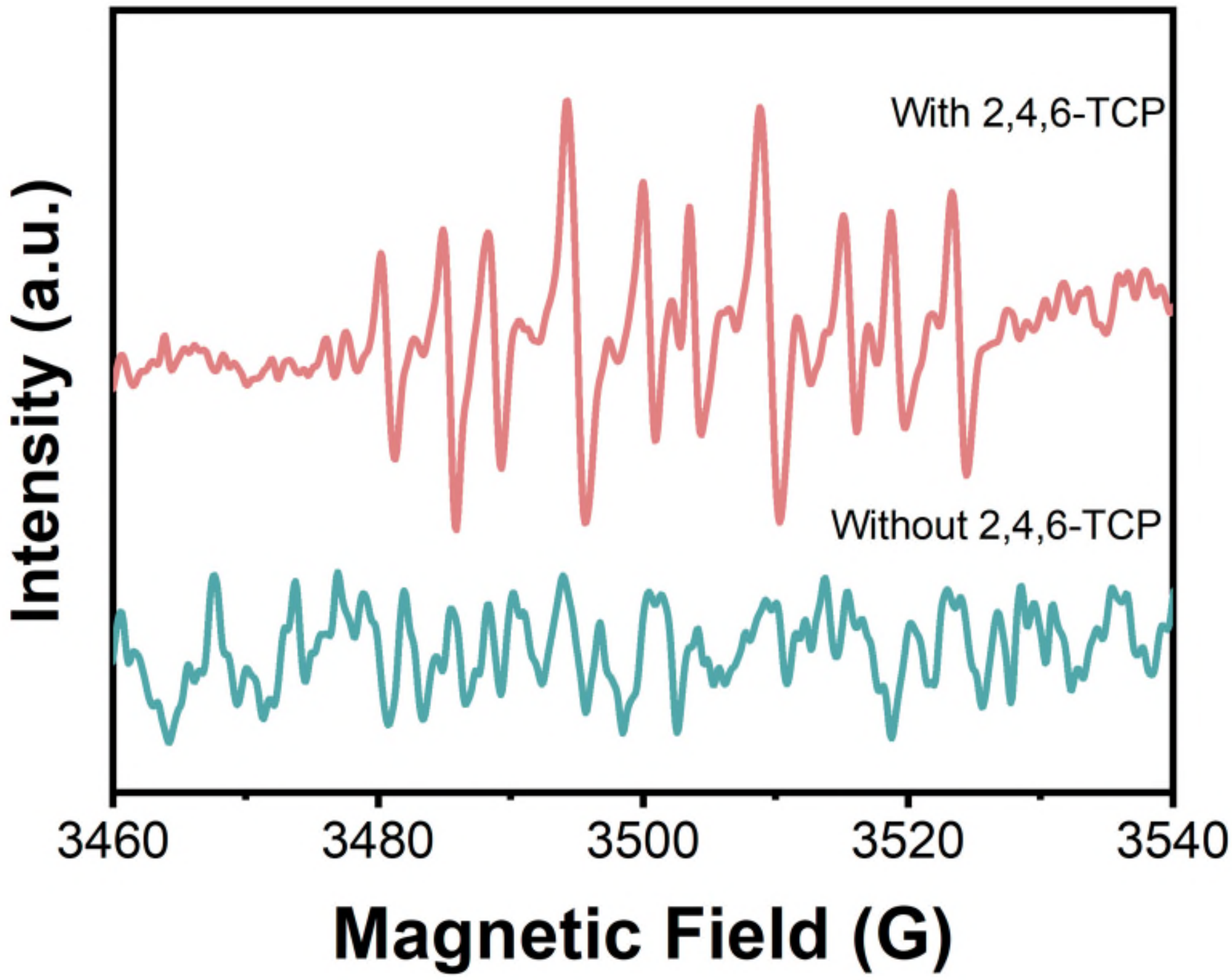

Fig.S10 EPR spectra of RCS trapped by PBN with and without 2,4,6-TCP after reacting for 10 min.

In lines 346-351, the sentence " As shown in Supplementary Fig.10, after 10 min of reaction, a clear Cl· signal was detected only in the system containing 2,4,6-TCP, while no obvious signal was observed in the absence of the pollutant. This comparison suggests that the Cl^- in solution originated from the dechlorination of 2,4,6-TCP and was subsequently transformed into Cl· during the reaction." was added.

Comment 3-2: What is the Faradaic efficiency of CER vs OER at 1.5 V?

Response: Thank you for this valuable suggestion. We agree that distinguishing the relative contributions of CER and OER at 1.5 V is important for clarifying the anodic reaction pathway. However, it should be noted that the 1.5 V used in this study refers to the applied cell voltage of a two-electrode system rather than a strictly defined anodic potential. In addition, the Cl^- in our system is not supplied as a fixed external component, but is continuously released from cathodic dechlorination and remains dynamically variable during the reaction. Moreover, the generated RCS are rapidly consumed by the pollutants. Under such conditions, a rigorous absolute quantification of the Faradaic efficiencies of CER and OER in the real degradation

system is difficult. If calculated only from the accumulated free chlorine, the result would represent merely an apparent lower bound of CER and would not reflect the full extent of chlorine-related oxidation processes.

Therefore, we adopted a semi-quantitative attribution strategy based on multiple control experiments and mechanistic analyses to evaluate the contributions of chloride reutilization and RCS-mediated oxidation. First, the quenching results in Fig.3c together with the EPR analysis confirmed the presence of RCS. Furthermore, as shown in Fig.3g, we supplemented a comparative quenching experiment using 5 mM nitrobenzene (NB), which is effective for quenching ·OH but has little effect on RCS. The results showed that the degradation efficiency decreased by only 8.7% after the addition of NB, whereas it decreased by 40.6% after the addition of MeOH, which can quench both ·OH and Cl·. This comparison indicates that RCS, rather than ·OH, plays the dominant role in the oxidation step. In addition, we further carried out oxidation experiments using a simulated post-reduction electrolyte. As shown in Fig.3g, in the presence of Cl^-, the degradation efficiencies of 2,4,6-TCP and phenol, as well as the TOC removal efficiency, were all significantly higher than those observed in the Cl^--free system. Taken together, although we are currently unable to provide rigorously separated Faradaic efficiency values for CER and OER, these results strongly support that the RCS-mediated oxidation pathway plays a dominant role in the mineralization process of this system.

The Fig.3 was revised into:

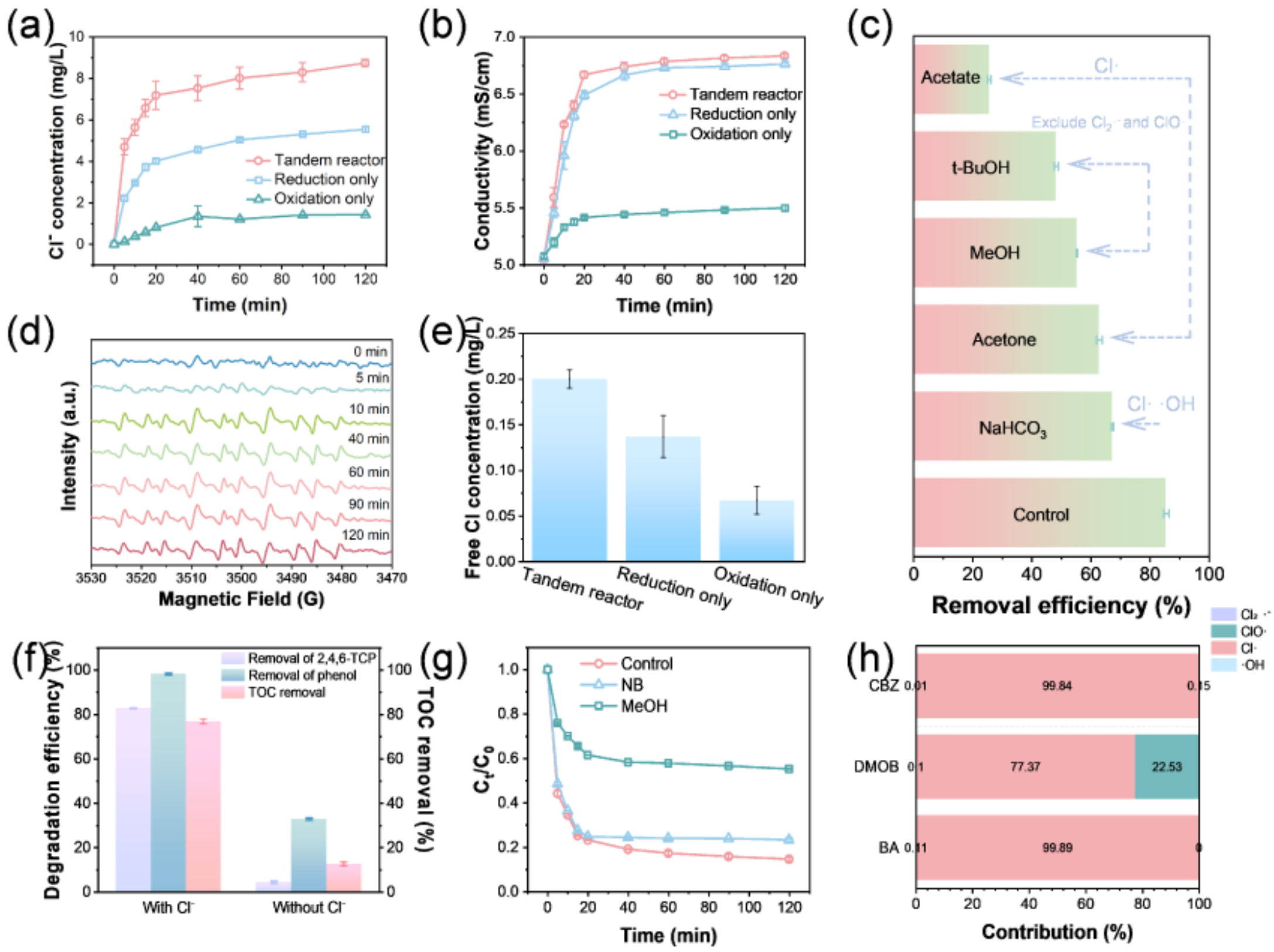

Fig.3 Evidence of the conversion of free chlorine ions to RCS. a,b, (a) Cl^- concentration and (b) Time-dependent variation in electrical conductivity in oxidation only, reduction only, and tandem reactor. **c,** 2,4,6-TCP quenching experiment in tandem reactor. **d,** EPR spectra of RCS trapped by PBN. **e,** Free Cl concentration in oxidation only, reduction only, and tandem reactor. **f,** The degradation efficiency and the TOC removal of simulated electrolyte after reduction-only reaction in the presence or absence of Cl^-. **g,** Quenching of ·OH: [MeOH] = [NB] = 5 mM. **h,** Relative radical contributions to the probe decay of BA, DMOB, and CBZ derived from probe-kinetic analysis.

In lines 432-453, the paragraph "To semi-quantitatively evaluate the contribution of the RCS-mediated oxidation process to the overall performance enhancement of the system, the composition of the electrolyte after the reduction-only reaction was first analyzed, which mainly contained Cl^-, 2,4,6-TCP, and its representative dechlorinated intermediate, phenol. Based on this composition, a simulated electrolyte was further prepared, and oxidation-only experiments were conducted in the presence and absence of Cl^- to investigate the role of RCS-

mediated oxidation in the overall system performance. As shown in Fig.3f, in the presence of Cl^-, the removal efficiencies of 2,4,6-TCP and phenol reached 82.8% and 98.3%, respectively, and the TOC removal efficiency reached 76.72%, which was comparable to the mineralization performance of the redox tandem system. In contrast, in the absence of Cl^-, the TOC removal efficiency was only 12.46%. This result indicates that the presence of Cl^- provided an additional 60.4 percentage points of TOC removal enhancement during the subsequent oxidation stage, strongly suggesting that Cl^--derived RCS play a dominant role in deep oxidation and mineralization. Combined with the reduction-only results, it can be inferred that sequential dechlorination mainly contributes to C–Cl bond cleavage, precursor transformation, and detoxification, whereas RCS-mediated oxidation is primarily responsible for the subsequent ring opening and deep mineralization. Therefore, the performance enhancement of the tandem system arises from the sequential synergy between these two processes, with RCS-mediated oxidation being the key contributor to TOC removal improvement." was added.

In lines 461-466, the paragraph " As shown in Fig.3g, the addition of nitrobenzene (NB), a quencher that is highly reactive toward ·OH but has negligible effect on RCS, resulted in only an 8.7% decrease in degradation efficiency.[56] In contrast, the addition of MeOH, which can quench both ·OH and Cl·, caused a much larger decrease of 40.6%. This result indicates that RCS plays a more dominant role than ·OH in the oxidation step." was added.

Comment 3-3: Can the author quantify relative contributions of Cl·, $Cl_2^{\cdot-}$, ClO·, and ·OH?

Response: Thank you for this important comment. We agree that distinguishing the relative roles of Cl·, $Cl_2^{\cdot-}$, ClO·, and ·OH is valuable for clarifying the oxidation mechanism in the tandem system. In response, we further carried out a semi-quantitative probe-based kinetic analysis to evaluate the relative contributions of these reactive species. Specifically, NB, BA, DMOB, and CBZ were used as stepwise discriminative probes to estimate the apparent steady-state concentrations and the corresponding pseudo-first-order contribution rates of ·OH, Cl·, ClO·, and $Cl_2^{\cdot-}$. The results showed that, in the Cl^--containing system, the apparent steady-state concentration of Cl· reached 6.37×10^{-13} M, which was substantially higher than that of ·OH, while ClO· and $Cl_2^{\cdot-}$ were also detected at lower levels. Based on the probe-kinetic-derived contribution analysis (Fig. 3h), Cl· accounted for the dominant fraction of the oxidation process,

whereas ClO· made a secondary contribution and the roles of $Cl_2^{\cdot -}$ and ·OH were comparatively minor. For example, for BA, the radical-mediated decay was dominated by Cl· (99.89%), with only a negligible contribution from ·OH (0.11%). For DMOB, Cl· and ClO· contributed 77.37% and 22.53%, respectively, whereas ·OH contributed only 0.10%. For CBZ, Cl· contributed 99.84%, while the contributions of ClO·, $Cl_2^{\cdot -}$, and ·OH were all minimal. Taken together, these results indicate the following contribution trend under the present tandem conditions: Cl· > ClO· >> $Cl_2^{\cdot -}$ ≈ ·OH. It should be noted that these values are derived from probe-based kinetic analysis and therefore should be interpreted as semi-quantitative descriptors of the relative roles of reactive species, rather than a strict absolute partition of all reaction pathways for the target pollutant. Nevertheless, they clearly demonstrate that Cl· is the dominant reactive species in the oxidation step of this tandem system. The corresponding results and discussion have been added to the revised manuscript and Supplementary Information.

The Fig.3 was revised into:

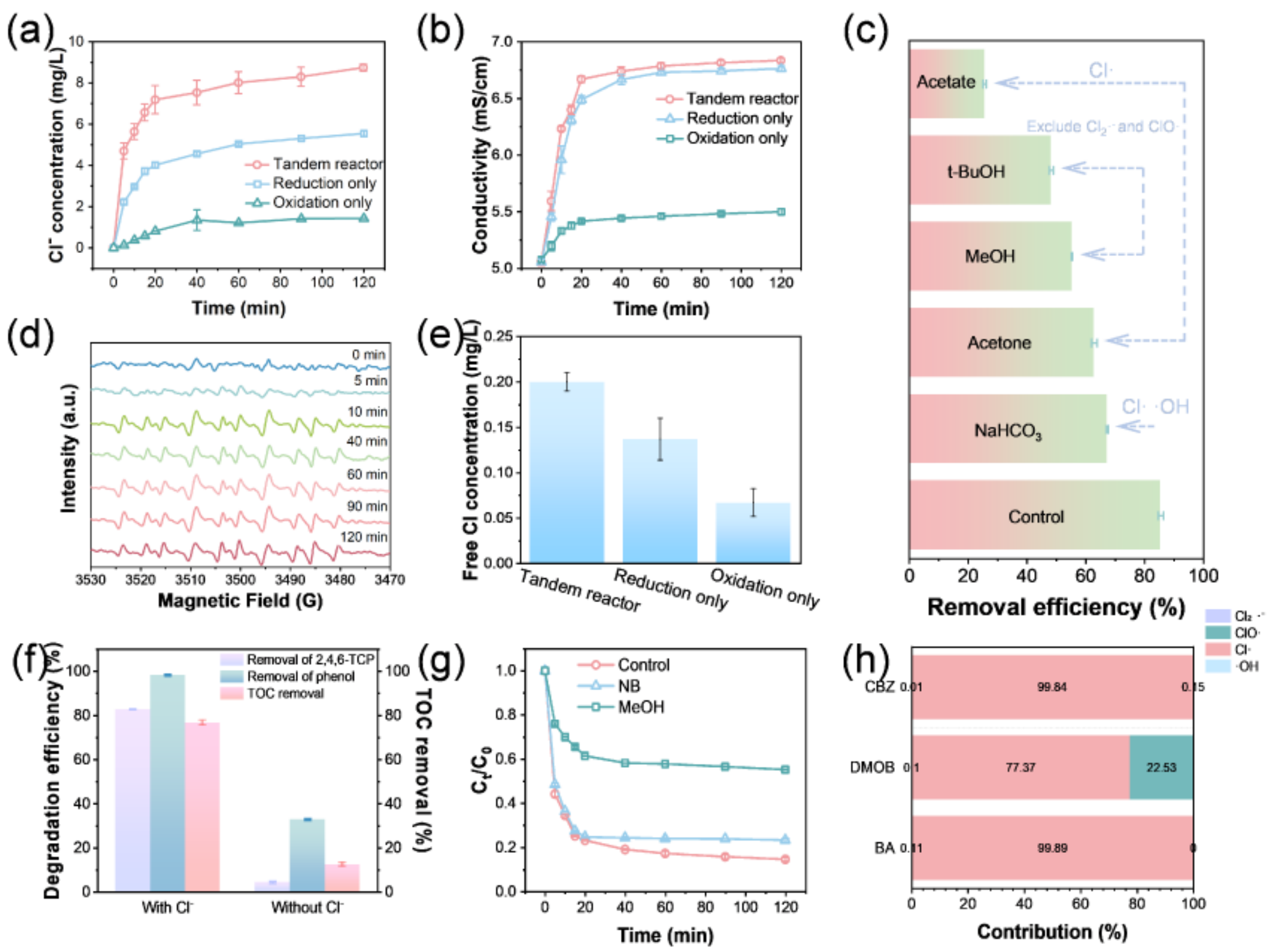

Fig.3 Evidence of the conversion of free chlorine ions to RCS. a,b, (a) Cl^- concentration and (b) Time-dependent variation in electrical conductivity in oxidation only, reduction only, and tandem

reactor. **c,** 2,4,6-TCP quenching experiment in tandem reactor. **d,** EPR spectra of RCS trapped by PBN. **e,** Free Cl concentration in oxidation only, reduction only, and tandem reactor. **f,** The degradation efficiency and the TOC removal of simulated electrolyte after reduction-only reaction in the presence or absence of Cl^-. **g,** Quenching of ·OH: [MeOH] = [NB] = 5 mM. **h,** Relative radical contributions to the probe decay of BA, DMOB, and CBZ derived from probe-kinetic analysis.

The Table 1 was added into the revised manuscript:

	·OH (10^{-16} M)	Cl·(10^{-13} M)	ClO· (10^{-13} M)	$Cl_2^{\cdot -}$ (10^{-14} M)
Without Cl^-	8.92	0.00	0.00	0.00
With Cl^-	2.11	6.37	1.59	4.88

The Table S4 was added into the Suplementary Information:

species	$k_{OH+non\text{-}radical\ pathway}$ (10^{-4} min^{-1})	k_{OH} (10^{-4} min^{-1})	$k_{non\text{-}radical\ pathway}$ (10^{-14} min^{-1})
NB	3.22	2.09	1.13
BA	6.91	3.16	3.75
DMOB	4.91	3.75	1.16
CBZ	8.94	4.71	4.23
TA	2.36	2.36	0.00

Table S5. The decay rates of NB, BA, CBZ and DMOB with Cl^-.

k_{NB} (10^{-4} min^{-1})	k_{BA} (10^{-2} min^{-1})	k_{DMOB} (10^{-2} min^{-1})	k_{CBZ} (min^{-1})
1.62	6.92	8.90	1.26

In lines 459-478, we added the paragraph: "To further clarify the relative contributions of different reactive species during the oxidation process, both scavenging experiments and probe-based kinetic analysis were employed. As shown in Fig.3g, the addition of nitrobenzene (NB), a quencher that is highly reactive toward ·OH but has negligible effect on RCS, resulted in only an 8.7% decrease in degradation efficiency.[56] In contrast, the addition of MeOH, which can quench both ·OH and Cl·, caused a much larger decrease of 40.6%. This result indicates that RCS plays a more dominant role than ·OH in the oxidation step. Furthermore, NB, BA, CBZ, and DMOB were used as probe compounds to semi-quantitatively evaluate the apparent steady-state concentrations of ·OH, Cl·, ClO·, and $Cl_2^{\cdot-}$, respectively, as summarized in Table 1.[39] In the oxidation system without Cl^- and 2,4,6-TCP, no obvious RCS signal was detected. In contrast, in the Cl^--containing system, the apparent steady-state concentration of Cl· reached 6.37×10−13 M, which was markedly higher than that of ·OH, indicating the dominant role of Cl· in this system. The relative contribution analysis derived from probe kinetics (Fig.3h) further showed that Cl· accounted for the major fraction of the oxidation process, while ClO· made a secondary contribution, and the roles of $Cl_2^{\cdot-}$ and ·OH were comparatively minor. Taken together, these results demonstrate that the oxidation process in the tandem system was mainly driven by RCS, among which Cl· was the dominant reactive species.".

In lines 865-906, we added the paragraph: "Based on previous studies, probe-based kinetic experiments were carried out under oxidation-only conditions to semi-quantitatively evaluate the relative contributions of different reactive species during the oxidation step.[39] A Cl^--free blank system and a Cl^--containing target system were established using 50 mM Na_2SO_4 as the supporting electrolyte, with the Cl^- concentration in the target system set to that measured in the post-reaction electrolyte of the reduction-only system. All other reaction conditions were kept identical to those used in the corresponding oxidation experiments. Terephthalic acid (TA, 0.5 mM) was used as the probe for ·OH, while nitrobenzene (NB), benzoic acid (BA), 1,4-dimethoxybenzene (DMOB), and carbamazepine (CBZ) were employed as stepwise discriminative probes for ·OH、Cl·、ClO· and $Cl_2^{\cdot-}$, respectively, each at an initial concentration of 50 μM. Each probe was tested individually and was not co-present with the target pollutant. Their concentrations

were determined by liquid chromatography, and the specific analytical method was adopted with reference to previous studies.[60] Aliquots were collected at predetermined time intervals, filtered, and then subjected to quantitative analysis. The apparent first-order rate constant, k_{obs}, for each probe was obtained by linear fitting according to:

$$ln\left(\frac{C_t}{C_0}\right)=-k_{obs}t \quad (2)$$

t denoted the reaction time, C_t denoted the concentration at that time, and C_0 denoted the initial concentration of the probe.

In the Cl^--free blank system, the apparent steady-state concentration of ·OH was first estimated from the apparent rate constant of TA:

$$[\cdot OH]_{SS}=\frac{k_{TA}}{k_{TA,\cdot OH}} \quad (3)$$

where $k_{TA,\cdot OH}$ was the second-order reaction rate constant between TA and ·OH (4.4×10^9 M^{-1} s^{-1})[61] and k_{TA} was the measured rate constant for TA oxidation in the blank system. Subsequently, the decay rates of NB、BA、DMOB and CBZ measured in the blank system were used to subtract the non-radical loss term, $k_{non\text{-}radical}$, for each probe (Tables S4 and S5). In the Cl^--containing target system, the apparent rate constants of NB, BA, DMOB, and CBZ were further used in a stepwise subtraction manner to derive $[\cdot OH]_{SS}$, $[Cl\cdot]_{SS}$, $[ClO\cdot]_{SS}$ and$[ClO_2\cdot^-]_{SS}$. Specifically, NB was used to calculate $[\cdot OH]_{SS}$. BA was used to determine $[Cl\cdot]_{SS}$ after subtracting the contribution of ·OH. DMOB was used to calculate $[ClO\cdot]_{SS}$ after further subtracting the contributions of ·OH and Cl·, and CBZ was finally used to determine $[ClO_2\cdot^-]_{SS}$ after subtracting the contributions of the first three species.The results are summarized in Table S6. Since this method is based on apparent rate constants with background correction using the blank system, the obtained values are more appropriately interpreted as semi-quantitative descriptors of the relative roles of reactive species rather than strict absolute concentrations. The contributions of each free radical are calculated by the following formula:

$$Contribution_i = \frac{k_i}{\sum k_{radicals}}\times 100\% \quad (4)$$

i represented each free radical, and "contribution" indicated its relative contribution.”.

Comment 3-4: What is the energy consumption per mg TOC removed?

Response: Thank you for raising this question. Based on our experimental data, we calculated the energy consumption per mg of TOC removed. Under the experimental conditions, the current was 0.003 A, the voltage was 1.5 V, the experiment lasted for 2 hours, and the electrolyte volume was 100 mL. The calculation shows that the energy consumption per mg of TOC removed is 0.022 J/mg TOC. This result reflects the energy efficiency of our system and demonstrates the energy required in the process of TOC removal. The relevant discussion has been included in the manuscript.

In lines 322-325, the sentence "The energy consumption per mg of TOC removed in our system was calculated to be 0.022 J/mg TOC, this indicates that the system is significantly more energy-efficient than conventional electro-oxidation processes.[46]" was added.

In lines 792-797, the paragraph "To calculate the energy consumption per mg of TOC removed, the following step was performed:

$$Energy\ per\ mg\ TOC\ removed = \frac{V \times I \times t}{TOC\ removed} \quad (1)$$

Where E is the energy consumed (J), V is the applied voltage (V), I is the current displayed at this voltage (A), TOC removed is the difference between the initial TOC and the final TOC (mg) and t is the duration of the reaction (s)." was added.

Comment 3-5: How stable is PdCo/CP over multiple cycles? Any metal leaching?

Response: We are very grateful for your professional review of our article. We have supplemented the data on cyclic experiments and metal leaching. The results show that PdCo/CP exhibited good electrochemical stability, maintaining a degradation efficiency of approximately 85% even after ten replicate experiments, and ICP analysis results indicated that very little cobalt, palladium, and ruthenium leached out. We have made the following additions to the relevant sections of the manuscript and supplementary information.

In lines 266-270, the sentence "To further evaluate the stability of the electrodes, cycling experiments were performed using the same electrode. As shown in Supplementary Fig.9, after ten consecutive cycles, the degradation efficiency still remained at approximately 85%, while the leaching concentrations of Co, Pd, and Ru were only 0.02, 0.01, and 0.01 mg/L, respectively,

demonstrating that the system can operate stably." was added.

The Supplementary Fig. 9 was added in Supplementary Information.

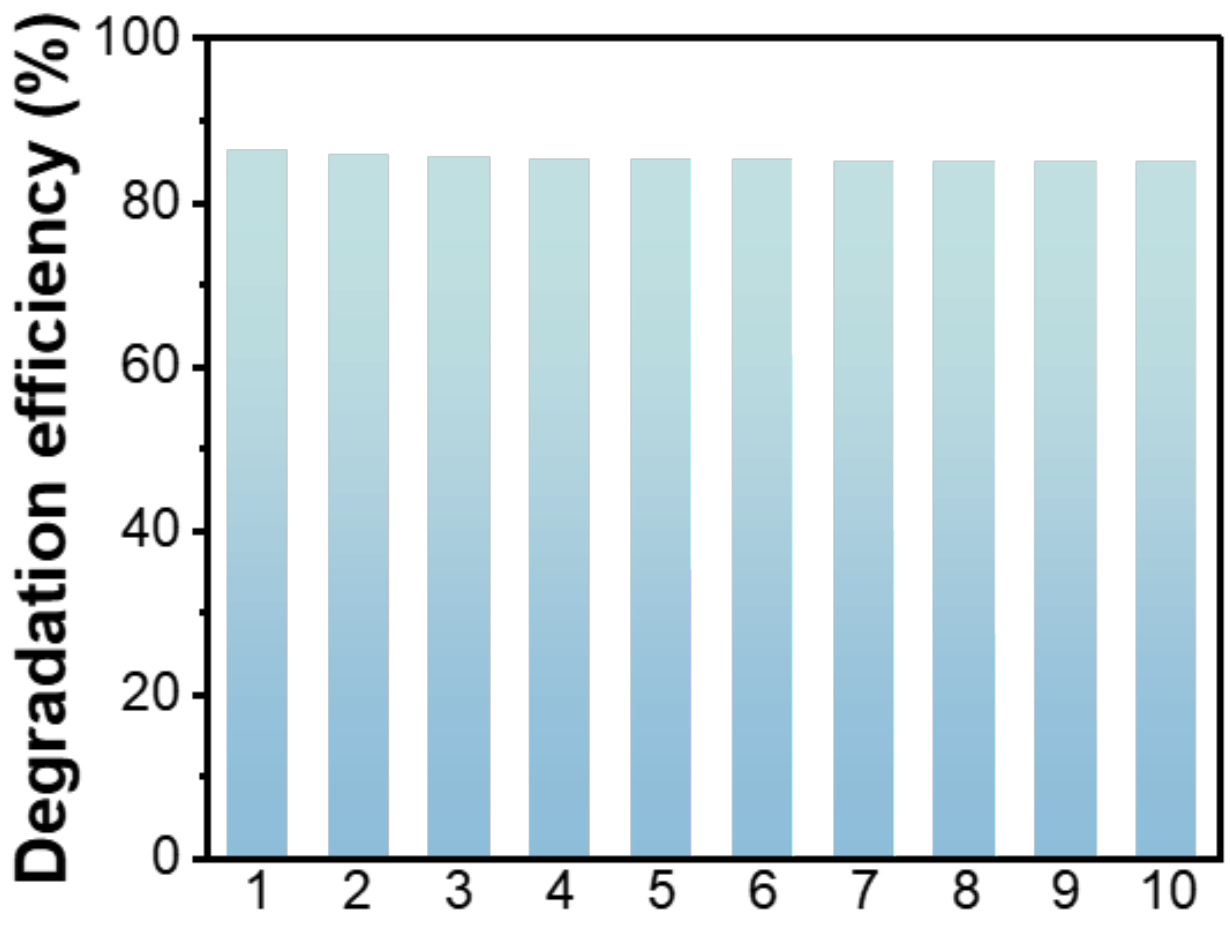

Fig.S9 The stability test with the cascade reactor.

Comment 3-6: How does performance change with initial chloride concentration?

Response: Thank you for this insightful question. The performance of our system is indeed influenced by the initial chloride concentration. As the Cl^- concentration increased from 0 to 8 mg/L, the TOC removal efficiency continuously increased and finally reached 92.44%. This trend further demonstrates a clear positive correlation between the amount of available Cl^- and the extent of mineralization. At low chloride concentrations, the generation of RCS, such as Cl· and ClO·, was limited, which resulted in slower degradation efficiency of the target pollutants like 2,4,6-TCP. As the initial chloride concentration increased, the generation of Cl· and ClO· at the anode improved, leading to a significant enhancement in degradation efficiency. This was because chloride recycling became more efficient with higher chloride availability. We have included these observations in the revised manuscript, along with data showing the relationship between chloride concentration and degradation efficiency, which we believe supports the role of chloride recycling in enhancing the performance of the system.

The Supplementary Fig.12 was added in supplementary information.

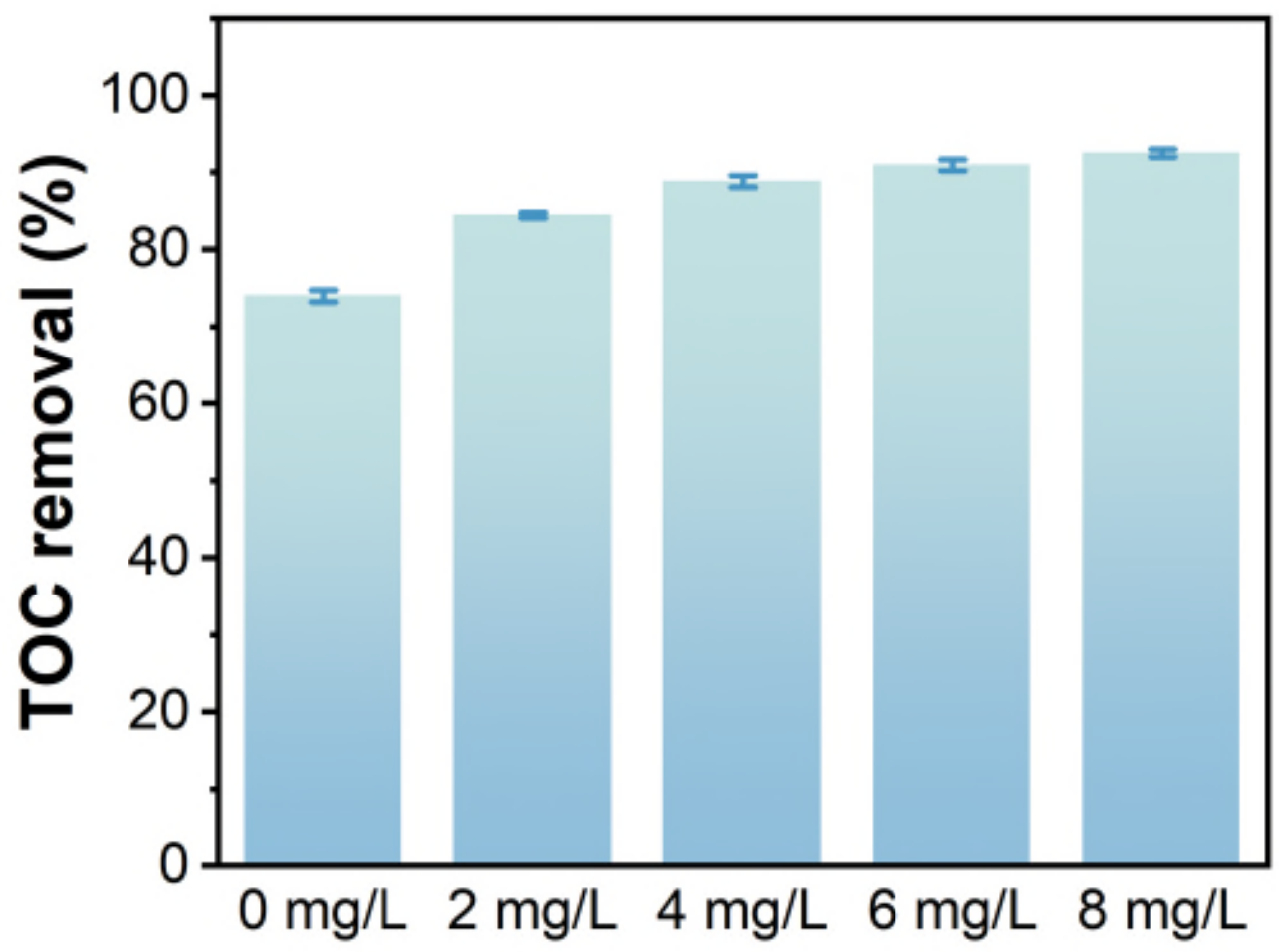

Fig.S12 The TOC removal of different additional Cl^- concentrations

In lines 453-458, the paragraph "With the addition of external Cl^- to the electrolyte, the TOC removal efficiency further increased from 73.97% to 92.45% as the chloride concentration increased (Supplementary Fig.12). This result suggests that a higher Cl^- concentration provides more available precursors for RCS generation, thereby promoting subsequent deep oxidation and enhancing the mineralization efficiency." was added.

Comment 3-7: Does the system work for non-phenolic COPs or aliphatic chlorinated compounds?

Response: Thank you very much for this constructive comment. In response to your suggestion, we have supplemented the degradation data for several representative COPs as well as aliphatic chlorinated compounds. The results show that the present system exhibits good degradation performance toward a variety of chlorinated organic compounds. At the same time, we observed that the TOC removal efficiency decreased as the chlorine content of the target pollutant decreased. We attribute this trend mainly to the reduced release of Cl^- during dechlorination, which lowers the concentration of reusable chloride ions in the system and consequently weakens the subsequent RCS-dominated oxidative mineralization process. The corresponding results and discussion have been added to the revised manuscript and the Supplementary

Information.

The Supplementary Fig.11 was added into Supplementary Information:

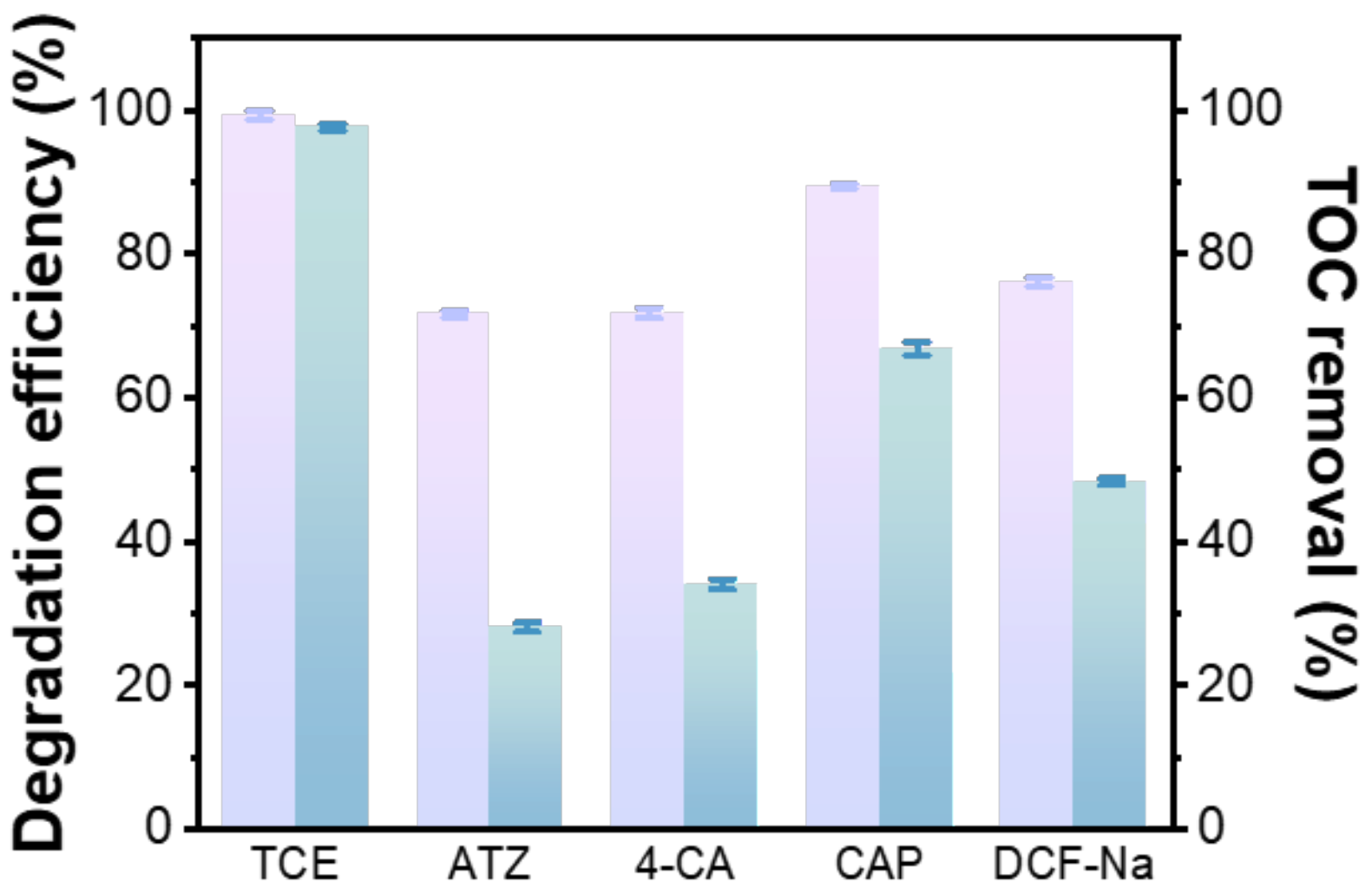

Fig.S11 The degradation efficiency and the TOC removal of non-phenolic COPs and aliphatic chlorinated compound.

In lines 416-431, the paragraph "To further evaluate the applicability of the tandem system, several representative non-phenolic chlorinated organic pollutants and an aliphatic chlorinated compound, including trichloroethylene (TCE), atrazine (ATZ), 4-chloroaniline (4-CA), chloramphenicol (CAP), and diclofenac sodium (DCF-Na), were selected for investigation. The results showed that the system maintained high degradation efficiencies toward these substrates, whereas the TOC removal efficiencies varied considerably (Supplementary Fig.11). This difference suggests that pollutant degradation and deep mineralization were not fully synchronous, and that mineralization depends not only on parent-compound conversion, but also on the amount of releasable chloride and the structural complexity of the target molecules. For substrates capable of releasing more Cl^- during cathodic dechlorination, more precursors could be supplied for subsequent anodic generation of RCS, thereby promoting deeper oxidation and mineralization. These results further support the tandem mechanism in which cathodic dechlorination-released Cl^- is reutilized at the anode to generate RCS for subsequent oxidative mineralization." was added.

In lines 712-716, the sentence was revised into "Potassium tetrachloropalladate (K_2PdCl_4),

sodium chloride (NaCl), sodium sulfate (Na_2SO_4), trichloroethylene (TCE), atrazine (ATZ), 4-chloroaniline (4-CA), chloramphenicol (CAP), diclofenac sodium (DCF-Na), sodium bicarbonate ($NaHCO_3$), acetate, acetone, sulfuric acid (H_2SO_4) and sodium hydroxide (NaOH) were purchased from Aladdin Reagent (Shanghai, China).".

In lines 762-776, the sentence was revised into "The specific detection conditions for 2,4,6-TCP, TCE, 4-CA, CAP, DFC-Na and ATZ are as follows.

2,4,6-TCP: The mobile phase consisted of methanol and ultrapure water with a ratio of 80:20, and a UV-vis detector at a wavelength of 245 nm.

TCE: The mobile phase consisted of acetonitrile and ultrapure water with a ratio of 50:50, and a UV-vis detector at a wavelength of 215 nm.

4-CA: The mobile phase consisted of methanol and ultrapure water with a ratio of 55:45, and a UV-vis detector at a wavelength of 240 nm.

CAP: The mobile phase consisted of sodium acetate and methanol with a ratio of 65:35, and a UV-vis detector at a wavelength of 445 nm.

DFC-Na: The mobile phase consisted of methanol and 0.1% acetic acid solution with a ratio of 60:40, and a UV-vis detector at a wavelength of 276 nm.

ATZ: The mobile phase consisted of methanol and ultrapure water with a ratio of 70:30, and a UV-vis detector at a wavelength of 221 nm.".

Comment 3-8: Why is free chlorine so low (~0.2 mg/L) if CER is significant?

Response: We appreciate your comment very much. We would like to clarify a fundamental aspect of our system. The process we describe is not simply a CER, but rather a chloride recycling process, where Cl^- released during cathodic hydrodechlorination is converted into RCS. RCS refers to reactive chlorine species, which include free radicals and free chlorine, all of which are involved in the oxidation and degradation of organic pollutants in our system. The low concentration of free chlorine (~0.2 mg/L) that we observe is not indicative of the inefficiency of CER, but rather reflects the rapid consumption of Cl· and ClO·, which are highly reactive and quickly consumed during the degradation of the target pollutant. Therefore, the presence of Cl· and ClO·, not free chlorine, is the key to the enhanced degradation performance in our system. We have focused on the degradation of organic pollutants using RCS rather than

just the chlorine evolution reaction. The low free chlorine concentration simply reflects that these reactive chlorine species are rapidly consumed in the process, while Cl· continue to play a dominant role in the overall degradation mechanism. We have now clarified this distinction in the revised manuscript, emphasizing that RCS generation and recycling are the key to the degradation process.

In lines 405-408, the sentence "Moreover, this low free chlorine concentration was due to the rapid consumption of Cl· and other RCS during the degradation process, where they were quickly involved in reactions with intermediates. It indicates that the chlorine species were continuously recycled and play a dominant role in the oxidation process." was added.

Comment 3-9: Could the observed enhancement simply be due to improved mass transfer, not Cl^- recycling?

Response: Thank you for raising this important question. Improved mass transfer does indeed affect the reaction rate. However, the observed enhancement in reaction performance is not solely attributed to improved mass transfer. To address this issue, we conducted a pure oxidation experiment using phenol, the dechlorination product, in a chloride-free electrolyte. The results showed that the reaction efficiency significantly improved only in the presence of Cl^-, and this enhancement cannot be solely explained by mass transfer improvement. Additionally, we have conducted radical quenching experiments and EPR observations, which strongly indicate that Cl· radicals play a dominant role in the reaction. These experiments confirm that the reactive chlorine species generated at the anode contribute significantly to the degradation process, supporting the hypothesis of Cl^- recycling rather than mass transfer improvement. We have added a discussion of these control experiments in the revised manuscript to further clarify that the observed performance enhancement is mainly due to Cl^- recycling and RCS generation, rather than mass transfer improvement.

The Fig.3 was revised into:

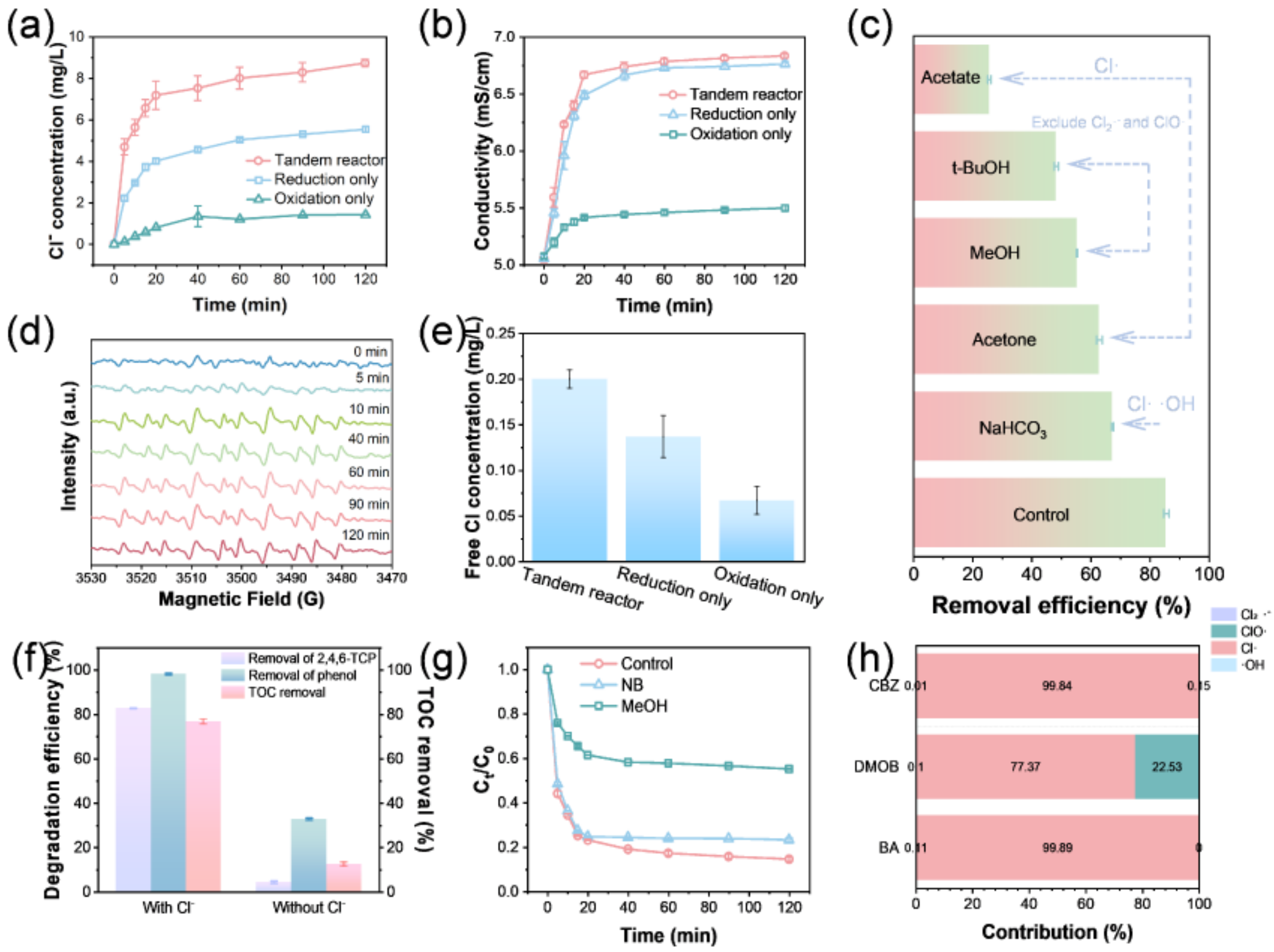

Fig.3 Evidence of the conversion of free chlorine ions to RCS. a,b, (a) Cl^- concentration and (b) Time-dependent variation in electrical conductivity in oxidation only, reduction only, and tandem reactor. **c,** 2,4,6-TCP quenching experiment in tandem reactor. **d,** EPR spectra of RCS trapped by PBN. **e,** Free Cl concentration in oxidation only, reduction only, and tandem reactor. **f,** The degradation efficiency and the TOC removal of simulated electrolyte after reduction-only reaction in the presence or absence of Cl^-. **g,** Quenching of ·OH: [MeOH] = [NB] = 5 mM. **h,** Relative radical contributions to the probe decay of BA, DMOB, and CBZ derived from probe-kinetic analysis.

In lines 432-453, the paragraph "To semi-quantitatively evaluate the contribution of the RCS-mediated oxidation process to the overall performance enhancement of the system, the composition of the electrolyte after the reduction-only reaction was first analyzed, which mainly contained Cl^-, 2,4,6-TCP, and its representative dechlorinated intermediate, phenol. Based on this composition, a simulated electrolyte was further prepared, and oxidation-only

experiments were conducted in the presence and absence of Cl^- to investigate the role of RCS-mediated oxidation in the overall system performance. As shown in Fig.3f, in the presence of Cl^-, the removal efficiencies of 2,4,6-TCP and phenol reached 82.8% and 98.3%, respectively, and the TOC removal efficiency reached 76.72%, which was comparable to the mineralization performance of the redox tandem system. In contrast, in the absence of Cl^-, the TOC removal efficiency was only 12.46%. This result indicates that the presence of Cl^- provided an additional 60.4 percentage points of TOC removal enhancement during the subsequent oxidation stage, strongly suggesting that Cl^--derived RCS play a dominant role in deep oxidation and mineralization. Combined with the reduction-only results, it can be inferred that sequential dechlorination mainly contributes to C–Cl bond cleavage, precursor transformation, and detoxification, whereas RCS-mediated oxidation is primarily responsible for the subsequent ring opening and deep mineralization. Therefore, the performance enhancement of the tandem system arises from the sequential synergy between these two processes, with RCS-mediated oxidation being the key contributor to TOC removal improvement." was added.
